# Aaptamine Derivatives with Antifungal and Anti-HIV-1 Activities from the South China Sea Sponge *Aaptos aaptos*

**DOI:** 10.3390/md12126003

**Published:** 2014-12-16

**Authors:** Hao-Bing Yu, Fan Yang, Fan Sun, Jing Li, Wei-Hua Jiao, Jian-Hong Gan, Wen-Zhen Hu, Hou-Wen Lin

**Affiliations:** 1 Laboratory of Marine Drugs, Department of Pharmacy, Changzheng Hospital, Second Military Medical University, Shanghai 200003, China; E-Mails: yuhaobing1986@126.com (H.-B.Y.); jhgan@shou.edu.cn (J.-H.G.); 2 Marine Drugs Research Center, Department of Pharmacy, State Key Laboratory of Oncogenes and Related Genes, Renji Hospital, School of Medicine, Shanghai Jiao Tong University, Shanghai 200127, China; E-Mails: bill1985@126.com (F.Y.); amelie.sunfan@gmail.com (F.S.); lijingjmu@163.com (J.L.); weihuajiao@hotmail.com (W.-H.J.); huwenzhen1006@126.com (W.-Z.H.)

**Keywords:** *Aaptos aaptos*, aaptamine, antifungal, anti-HIV-1 activity

## Abstract

Five new alkaloids of aaptamine family, compounds (**1**–**5**) and three known derivatives (**6**–**8**), have been isolated from the South China Sea sponge *Aaptos aaptos*. The structures of all compounds were unambiguously elucidated by spectroscopic analyses, as well as by comparison with the literature data. Compounds **1**–**2** are characterized with triazapyrene lactam skeleton, whereas compounds **4**–**5** share an imidazole-fused aaptamine moiety. These compounds were evaluated in antifungal and anti-HIV-1 assays. Compounds **3**, **7**, and **8** showed antifungal activity against six fungi, with MIC values in the range of 4 to 64 μg/mL. Compounds **7**–**8** exhibited anti-HIV-1 activity, with inhibitory rates of 88.0% and 72.3%, respectively, at a concentration of 10 μM.

## 1. Introduction

Marine invertebrates such as sponges are well-known sources of a wide variety of natural alkaloids [1]. The aaptamines are an intriguing group of biologically active marine alkaloids sharing a rare 1*H*-benzo[*de*]-1,6-naphthyridine skeleton [2]. Since the first and most prominent member of this family, aaptamine, was obtained from the marine sponge *Aaptos aaptos* by Nakamura *et al.* in 1982 [2], various aaptamine derivatives have been isolated from marine sponges belonging to the genera *Aaptos*, *Suberites*, *Luffariella*, *Hymeniacidon*, *Suberea*, and *Xestospongia* [3]. Because of their various biological activities, such as α-adrenoceptor blocking [4], anti-HIV-1 [5], antimicrobial [6], antiherpes [7], sortase A inhibitory [8], and cytotoxic activities [9,10], these alkaloids has become an interesting focus for synthesis, structure-activity relationship and bioactivity studies [3,11]. In particular, the genus *Aaptos* continues to be an abundant source of aaptamine alkaloids [12,13], which still spurs interest in finding new bioactive metabolites.

Our previous chemical investigation of the sponge *A. aaptos*, collected from the Xisha islands in the South China Sea, led to the isolation of a series of cytotoxic aaptamine derivatives [14]. As a continuation of our studies on aaptamine alkaloids, we carried out further chromatographic purification on the extracts of *A. aaptos*, and five new alkaloids **1**–**5** related to aaptamines, in addition to three known aaptamines **6**–**8** were isolated. Herein, we described the isolation, the structure determination of these compounds, as well as the evaluation of their antifungal and anti-HIV-1 activities.

## 2. Results and Discussion

**Figure 1:**
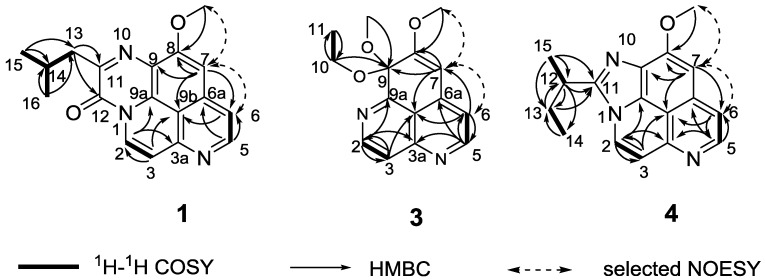
COSY, HMBC and selected NOESY correlations of compounds **1**, **3**, and **4**.

**Figure 2:**
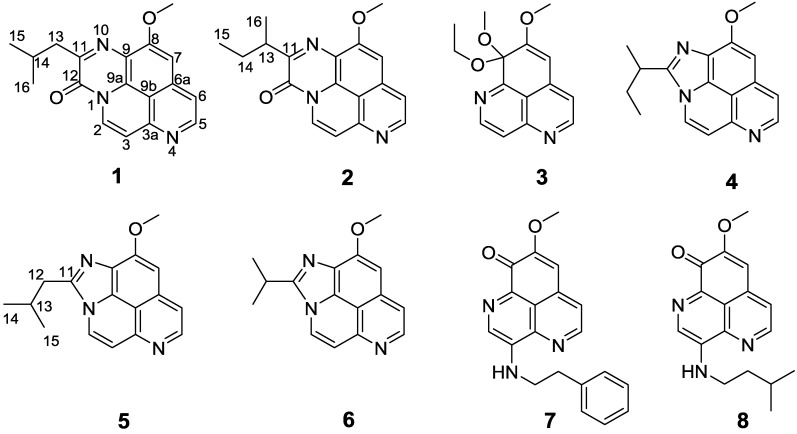
Structures of compounds **1**–**8**.

Compound **1** obtained as an amorphous yellow solid, revealed an [M + Na]^+^ peak at *m*/*z* 330.1220, suggesting a molecular formula of C_18_H_17_N_3_O_2_ and 12 degrees of unsaturation. The IR spectrum showed bands due to lactam carbonyl functions (1663 cm^−1^) [15], while the UV absorptions at 208, 224, 273, 282, 412, and 434 nm suggested the presence of triazapyrene lactam core in the molecule [13]. The ^1^H NMR spectrum displayed the characteristic signals for one isolated methoxy protons 8-OCH_3_ (δ_H_ 4.12, 3H, s), two sets of coupled protons at H-2 (δ_H_ 8.73, d, *J* = 8.0 Hz) and H-3 (δ_H_ 7.48, d, *J* = 8.0 Hz), and H-5 (δ_H_ 7.76, d, *J* = 5.5 Hz) and H-6 (δ_H_ 8.70, d, *J* = 5.5 Hz), and the isolated proton H-7 (δ_H_ 7.39, 1H, s), which can be found in most of aaptamine structures [13]. Unlike the other aaptamine analogs reported in the literature [2,14], the absence of proton 1-NH and methoxy protons 9-OCH_3_ in **1** indicated the substitutions of both N-1 and C-9. In addition, key HMBC correlations from H-2 to C-3a (δ_C_ 148.2) and C-9a (δ_C_ 126.7), from H-3 to C-2 (δ_C_ 126.5) and C-9b (δ_C_ 112.8), from H-5 to C-6 (δ_C_ 116.9) and C-6a (δ_C_ 137.1), from H-6 to C-5 (δ_C_ 147.4), C-7 (δ_C_ 99.2), and C-9b, from H-7 to C-6, C-8 (δ_C_ 157.3), C-9 (δ_C_ 119.5), and C-9b, and from 8-OCH_3_ to C-8 (Figure 1 and Supplementary Figure S5), confirmed that **1** was an aaptamine congener [13]. Moreover, the quaternary carbons at C-11 (δ_C_ 153.9) and C-12 (δ_C_ 150.7) were confirmed to be a sp^2^ quaternary carbon and a lactam carbonyl, respectively, by comparison the ^13^C NMR data with the reported aaptamine derivatives [13]. The quaternary carbon C-11 was further linked to C-9 via a nitrogen atom on consideration of its chemical shifts. Apart from these signals, one methine carbon (δ_C_ 26.7), one methylene carbon (δ_C_ 42.4), and two overlapped methyl carbons (δ_C_ 22.6, 22.6) remained unassigned. These signals were revealed to be an isobutyl group, supported by COSY correlations of H_2_-13 (δ_H_ 2.91)/H-14 (δ_H_ 2.34), H-14/H_3_-15 (δ_H_ 0.99), and H-14/H_3_-16 (δ_H_ 0.99). The isobutyl group was tethered to C-12 via C-11, based on the HMBC correlations from H_2_-13 to C-11 and C-12. Since all the above resonances accounted for 11 out of the 12 degrees of unsaturation, the remaining one degree of unsaturation was assumed to the presence of a tetracyclic system in **1**. Finally, a directly bridge between N-1 and C-12 could be rationalized the remaining one degree of unsaturation and the molecular formula. Therefore, the structure of compound **1** was unambiguously assigned as depicted in Figure 2.

Compound **2** was also obtained as a yellow solid. The molecular formula of C_18_H_17_N_3_O_2_ was deduced from its HRESIMS data (*m*/*z* 308.1400 [M + H]^+^) in combination with extensive NMR analyses. The NMR data for **2** indicated an overall structure similar to **1**, with the only difference at the side chain substituted to C-11 (Table 1 and Table 2). The ^1^H NMR data for **2** lacked two overlapped methyl doublets at δ_H_ 0.99, and instead one methyl doublet at δ_H_ 1.31 and one methyl triplet at δ_H_ 0.91 were evident. These data suggested that the isobutyl group in **1** was replaced by a *sec*-butyl group in **2**, which was supported by the COSY correlations of H_2_-14 (δ_H_ 1.65/1.94)/H_3_-15 (δ_H_ 0.91) and H-13 (δ_H_ 3.51)/H_3_-16 (δ_H_ 1.31), as well as the HMBC correlations from H_2_-14 and H_3_-15 to C-13 (δ_C_ 37.7), and from H-13, H_2_-14, and H_3_-16 to C-11 (δ_C_ 157.8).

**Table 1:** ^13^C NMR Data for Compounds **1**–**5**.

Carbon	1 ^a^	2 ^a^	3 ^b^	4 ^b^	5 ^b^
**2**	126.5	126.6	147.6	125.7	125.3
**3**	118.6	116.9	122.5	114.6	115.5
**3a**	148.2	148.3	149.6	145.6	146.2
**5**	147.4	147.5	156.1	144.8	144.4
**6**	116.9	116.9	117.8	116.2	116.2
**6a**	137.1	137.2	139.3	134.8	134.8
**7**	99.2	99.3	99.6	97.4	97.2
**8**	157.3	157.3	162.3	156.8	156.4
**9**	119.5	120.9	96.3	126.2	126.2
**9a**	126.7	126.5	155.7	129.4	129.4
**9b**	112.8	112.9	116.8	112.3	112.4
**8-OCH_3_**	56.3	56.4	56.1	56.6	56.5
**9-OCH_3_**			52.0		
**10**			60.0		
**11**	153.9	157.8	15.3	151.8	146.7
**12**	150.7	150.3		34.3	36.2
**13**	42.4	37.7		29.2	28.8
**14**	26.7	27.3		12.1	22.7
**15**	22.6	11.9		19.2	22.7
**16**	22.6	17.9			

^a^ Measured at 125 MHz in DMSO-*d*_6_; ^b^ Measured at 150 MHz in CDCl_3_.

Compound **3** was obtained as a yellow solid. The molecular formula was determined as C_15_H_16_N_2_O_3_ by an HRESIMS ion peak at *m*/*z* 295.1058 [M + Na]^+^. The ^1^H NMR spectrum revealed the resonances for two sets of coupled protons at δ_H_ 8.93 (d, *J* = 5.4 Hz) and 7.87 (d, *J* = 5.4 Hz), as well as 8.91 (d, *J* = 4.8 Hz) and 7.14 (d, *J* = 4.8 Hz), one singlet at δ_H_ 6.14, and two methoxy groups at δ_H_ 3.96 and 3.22, indicating the characteristic patterns for aaptamine congener [16]. The COSY and key HMBC correlations gave the postulated structure (Figure 1, Supplementary Figures S25 and S26). HMBC correlation from the methoxy (δ_H_ 3.22) to C-9 (δ_C_ 96.3) indicated that the methoxy was tethered to C-9. In addition, the remaining unassigned signals, one oxymethylene (δ_H_/δ_C_ 3.23 and 3.47/60.0), and one methyl (δ_H_/δ_C_ 1.13/15.3) were determined as an ethoxy group by COSY correlations of H-10a (δ_H_ 3.23)/H_3_-11 (δ_H_ 1.13) and H-10b (δ_H_ 3.47)/H_3_-11, which was placed at C-9 by the HMBC correlations from H-10a and H-10b to C-9. Based on these data, the structure of compound **3** can be unambiguously assigned as a methylethylketal derivative of demethyl(oxy)aaptamine as shown in Figure 2. Compound **3**, however, could possibly be an artifact from the extraction process with EtOH as a solvent.

**Table 2:** ^1^H NMR Data for Compounds **1**–**5** (*J* in Hz).

Position	1 ^a^	2 ^a^	3 ^b^	4 ^b^	5 ^b^
**2**	8.73, d (8.0)	8.76, d (8.0)	8.93, d (5.4)	8.27, d (7.2)	8.18, d (7.2)
**3**	7.48, d (8.0)	7.49, d (8.0)	7.87, d (5.4)	7.57, d (7.2)	7.43, d (7.2)
**5**	8.70, d (5.5)	8.71, d (5.5)	8.91, d (4.8)	8.65, d (6.0)	8.68, d (6.0)
**6**	7.76, d (5.5)	7.68, d (5.5)	7.14, d (4.8)	7.72, d (6.0)	7.43, d (6.0)
**7**	7.39, s	7.40, s	6.14, s	7.13, s	7.01, s
**8-OCH_3_**	4.12, s	4.13, s	3.96, s	4.29, s	4.26, s
**9-OCH_3_**			3.22, s		
**10a**			3.23, m		
**10b**			3.47, m		
**11**			1.13, t, (7.2)		
**12**				3.45, m	3.16, d (7.2)
**13a**	2.91, d (7.0)	3.51, m		1.96, m	2.44, m
**13b**				2.16, m	
**14a**	2.34, m	1.65, m		0.98, t (7.2)	1.08, d (7.2)
**14b**		1.94, m			
**15**	0.99, d (7.0)	0.91, t (7.0)		1.62, d (6.6)	1.08, d (7.2)
**16**	0.99, d (7.0)	1.31, d (7.0)			

^a^ Measured at 500 MHz in DMSO-*d*_6_; ^b^ Measured at 600 MHz in CDCl_3_.

Compound **4** was isolated as a yellow solid. The molecular formula was established as C_17_H_17_N_3_O on the basis of its HRESIMS spectrum, indicating the presence of 11 degrees of unsaturation. All 17 carbons were well resolved in the ^13^C NMR spectrum, and were classified by chemical shifts, DEPT and HSQC spectra as seven quaternary carbons (δ_C_ 156.8, 151.8, 145.6, 134.8, 129.4, 126.2, and 112.3), five olefinic methines (δ_C_ 144.8, 125.7, 116.2, 114.6, and 97.4), one methoxy (δ_C_ 56.6), one methine (δ_C_ 34.3), one methylene (δ_C_ 29.2), and two methyl groups (δ_C_ 19.2, 12.1). In the ^1^H NMR spectrum, the presence of two sets of coupled protons at H-2 (δ_H_ 8.27, d, *J* = 7.2 Hz) and H-3 (δ_H_ 7.57, d, *J* = 7.2 Hz), and H-5 (δ_H_ 8.65, d, *J* = 6.0 Hz) and H-6 (δ_H_ 7.72, d, *J* = 6.0 Hz), one isolated singlet at H-7 (δ_H_ 7.13, s), and one aromatic methoxy proton signal at 8-OCH_3_ (δ_H_ 4.29, s), indicated that compound **4** was an aaptamine congener, with an 1,9-disubstituted-8-methoxybenzo[*de*][1,6]naphthyridine skeleton [16]. Key HMBC correlations shown in Figure 1 confirmed this proposal structure (Supplementary Figure S35). Apart from these signals, two methyls (δ_H_/δ_C_ 0.98/12.1, and 1.62/19.2), one methylene (δ_H_/δ_C_ 1.96, 2.16/29.2), one methine (δ_H_/δ_C_ 3.45/34.3), and one quaternary sp^2^ carbon (δ_C_ 151.8) remained unassigned. Furthermore, COSY correlations of H_3_-14 (δ_H_ 0.98)/H-13a (δ_H_ 1.96), H_3_-14/H-13b (δ_H_ 2.16), and of H_3_-15 (δ_H_ 1.62)/H-12 (δ_H_ 3.45), together with the key HMBC correlations from H-13a and H-13b to C-12 (δ_C_ 34.3), from H_3_-14 to C-12, and from H-12 to C-13 (δ_C_ 29.2) established the presence of a *sec*-butyl group. Significant HMBC correlations from H-12, H-13a, H-13b, and H-15 to C-11 (δ_C_ 151.8) suggested the *sec*-butyl group connected to the quaternary sp^2^ carbon C-11 (Figure 1). On the basis of one still unassigned degree of unsaturation, the deshielded resonance of C-12, as well as the comparison of the ^13^C NMR data of the C-11 with the literature data of known alkaloids possess triaza-cyclopenta[*cd*]phenalene skeleton isolated by Laurent Calcul and co-workers [16], C-11 was directly tethered to N-1 and N-10. Therefore, the structure of compound **4** was unambiguously assigned. However, the absolute configuration of C-12 could not be definitively determined on the basis of spectral data.

Compound **5** was also isolated as a yellow solid, assigned a molecular formula of C_17_H_17_N_3_O, implying 11 degrees of unsaturation, as deduced from the HRESIMS spectrum. The ^13^C NMR and DEPT spectra exhibited 17 carbon resonances corresponding to two methyl, one methoxy, one methylene, six methine, and seven quaternary carbons. Moreover, the overall appearance of the NMR spectrum geared to compound **5** showed close structural similarity between **4** and **5**, indicating the same triaza-cyclopenta[*cd*]phenalene skeleton, except for that C-11 was substituted by an isobutyl group in **5**. This was also confirmed by the COSY correlations of H_3_-14 (δ_H_ 1.08)/H-13 (δ_H_ 2.44), H-13/H_3_-15 (δ_H_ 1.08), and H-13/H_2_-12 (δ_H_ 3.16), and the HMBC correlations from H_2_-12 and H-13 to C-11 (δ_C_ 146.7). Thus, compound **5** was identified as shown in Figure 2.

Besides the five new compounds **1**–**5**, the known compounds 2-isopropyl-10-methoxyimidazo[4,5,1-ij]pyrido[2,3,4-de]quinoline (**6**) [16], 3-(phenethylamino)demethyl(oxy)aaptamine (**7**) [10], and 3-(isopentylamino)demethyl(oxy)aaptamine (**8**) [10] were also obtained and elucidated by comparison of their physical and spectroscopic features with the data reported in the literature (Supplementary Table S1 and Figures S51–S56).

All the isolated compounds, except **1**–**2**, were tested for antifungal activity against six fungi, including *Candida parapsilosis*, *C. glabrata*, *C. albicans*, *Cryptococcus neoformans*, *Trichophyton rubrum*, and *Microsporum gypseum*, using fluconazole as a positive control (Table 3). Compound **7** showed antifungal activity against *C. albicans*, *C. parapsilosis*, *C. neoformans*, *T. rubrum*, and *M. gypseum* with MIC values of 32, 64, 32, 4, and 16 μg/mL, respectively. Compound **8** displayed potential antifungal activity against fungi *C. neoformans*, *T. rubrum*, and *M. gypseum* with MIC values of 64, 8, and 32 μg/mL, respectively. The rest compounds tested exhibited weak activity against the above fungi. In addition, these compounds, except **1**–**2**, were also assessed for their inhibitory activity against HIV-1 replication, compounds **6**–**8** inhibited 27.7%, 88.0%, and 72.3%, respectively, while the other isolates were inactive at the same concentration.

**Table 3:** Antifungal and Anti-HIV-1 Activities of Compounds **3**–**8**.

Compound	Antifungal Activity						Anti-HIV-1 Activity (10 μM)
	*C. albicans*	*C. parapsilosis*	*C. neoformans*	*C. glabrata*	*T. rubrum*	*M. gypseum*	
**3**	>64	32	>64	>64	> 64	> 64	NA
**4**	>64	> 64	>64	>64	> 64	64	NA
**5**	>64	> 64	>64	>64	> 64	64	NA
**6**	>64	> 64	>64	>64	> 64	64	27.7%
**7**	32	64	32	>64	4	16	88.0%
**8**	>64	>64	64	>64	8	32	72.3%
**fluconazole**	0.25	1.00	0.25	2	4	16	NT

NT, Not tested; NA, Not active.

## 3. Experimental Section

### 3.1. General Experimental Procedures

Optical rotation data were carried out on a Perkin-Elmer model 341 polarimeter (Perkin-Elmer Inc., Waltham, MA, USA) with a 1 dm cell. UV and IR (KBr) spectra were performed on a Hitachi U-3010 spectrophotometer (Hitachi Inc., Tokyo, Japan) and Jasco FTIR-400 spectrometer (Jasco Inc., Tokyo, Japan), respectively. ^1^H, ^13^C, DEPT135, COSY, HSQC, HMBC, and NOESY NMR spectra were recorded at room temperature (rt) on Bruker AVANCE-600 and Bruker AMX-500 instruments (Bruker Biospin Corp., Billerica, MA, USA). HRESIMS and ESIMS data were obtained on a Waters Q-Tof micro YA019 mass spectrometer (Waters Corp., Milford, MA, USA). Reversed-phase HPLC was performed on YMC-Pack Pro C18 RS (5 μm) columns (YMC Co. Ltd., Kyoto, Japan) with a Waters 1525 separation module (Waters Corp., Milford, MA, USA) equipped with a Waters 2998 Photodiode Array (PDA) detector (Waters Corp., Milford, MA, USA). Silica gel (200-300 mesh, Qingdao Ocean Chemical Co., Qingdao, China), Sephadex LH-20 (18-110 μm, Pharmacia Co., Piscataway, NJ, USA), and ODS (50 μm, YMC Co. Ltd., Kyoto, Japan) were used for column chromatography. TLC was carried out using HSGF 254 plates (Qingdao Ocean Chemical Co., Qingdao, China) and visualized by spraying with anisaldehyde-H_2_SO_4_ reagent.

### 3.2. Animal Material

The sponge samples were collected off Woody (Yongxing, Hainan, China) Island and Seven Connected Islets in the South China Sea in June 2007, and identified as *A. aaptos* by Jin-He Li (Institute of Oceanology, Chinese Academy of Sciences, Qingdao, China), A voucher sample (No. OLS) was deposited in the Laboratory of Marine Drugs, Department of Pharmacy, Changzheng Hospital, Second Military Medical University, Shanghai, China.

### 3.3. Extraction and Isolation

The air-dried sponge (2.3 kg, dry weight) was powdered, and extracted with 95% aqueous EtOH at rt. The combined extracts were concentrated under reduced pressure to yield the crude extract (109.7 g), which was suspended in H_2_O and extracted with CH_2_Cl_2_ and *n*-BuOH to afford the CH_2_Cl_2_- and *n*-BuOH-soluble extracts. The CH_2_Cl_2_-soluble extract (84.4 g) was partitioned between 90% aqueous MeOH and *n*-hexane, and *n*-hexane layer was collected and concentrated under reduced pressure to afford the *n*-hexane-soluble extract (52.0 g). The 90% aqueous MeOH phase was diluted to 60% aqueous MeOH with H_2_O, which was extracted with CH_2_Cl_2_ to afford the CH_2_Cl_2_-soluble extract (14.2 g). The CH_2_Cl_2_-soluble extract was subjected to vacuum liquid chromatography (VLC) on silica gel by gradient elution using CH_2_Cl_2_/MeOH (100:1, 80:1, 60:1, 50:1, 30:1, 20:1, 10:1, 8:1, 5:1, 1:1, 0:1, v/v) as solvents to give seven fractions (A–G). Fraction B (1.8 g) was subjected to column chromatography (CC) on Sephadex LH-20 with CH_2_Cl_2_/MeOH (1:1) as the eluting solvent to afford six subfractions (B1–B6). Subfraction B3 was separated by column chromatography (CC) on ODS (50 μm) eluting with MeOH-H_2_O (2:3–1:0) to give six fractions (Fr.B31–B36). Compound **3** (13.0 mg) was obtained by preparative TLC from Fr.B33. Fraction D (1.8 g) was separated by CC on silica gel eluting in a stepwise manner with CH_2_Cl_2_/MeOH (60:1, 50:1, 30:1, 15:1, 10:1, 5:1, 0:1, v/v) to afford six fractions (D1–D6). Fraction D4 was purified by reversed-phase HPLC with an elution of 50% MeCN detected at the wavelength of 249 nm to give metabolites **7** (2.0 mL/min, *t*_R_ = 11.5 min, 18.6 mg), and **8** (2.0 mL/min, *t*_R_ = 12.8 min, 5.8 mg). Fraction D5 were isolated by reversed-phase HPLC eluting with 50% MeCN detected at 254 nm to afford **6** (2.0 mL/min, *t*_R_ = 28.5 min, 1.5 mg), **4** (2.0 mL/min, *t*_R_ = 30.3 min, 2.5 mg), and **5** (2.0 mL/min, *t*_R_ = 31.5 min, 1.5 mg), whereas the purification of D6 by reversed-phase HPLC eluting with 80% MeCN detected at 244 nm resulted in the isolation of **1** (2.0 mL/min, *t*_R_ = 15.0 min, 2.3 mg), **2** (2.0 mL/min, *t*_R_ = 16.3 min, 2.0 mg).

2-Isobutyl-11-methoxy-3*H*-[1,6]naphthyridino[6,5,4-*def*]quinoxalin-3-one (**1**): yellow solid; UV (MeOH) (log ε) λ_max_ 208 (4.31), 224 (4.36), 273 (3.96), 282 (4.22), 412 (4.18), 434 (4.12); IR (KBr) ν_max_ 3404, 2955, 2926, 2856, 1730, 1663, 1638, 1597, 1527, 1472, 1387, 1320, 1256, 1174, 1098, 1010, 849 cm^−1^; ^1^H and ^13^C NMR data, see Table 1 and Table 2; HRESIMS *m*/*z* 330.1220 [M + Na]^+^ (calcd for C_18_H_17_N_3_O_2_Na, 330.1218).

2-(*Sec*-butyl)-11-methoxy-3*H*-[1,6]naphthyridino[6,5,4-*def*]quinoxalin-3-one (**2**): yellow solid; ${\left[\text{α}\right]}_{\text{D}}^{25}$ 3.10 (*c* 0.10, MeOH); UV (MeOH) (log ε) λ_max_ 204 (3.82), 224 (3.78), 273 (3.56), 282 (3.59), 391 (3.35), 410 (3.53), 432 (3.49); IR (KBr) ν_max_ 3371, 2960, 2927, 2855, 1728, 1665, 1637, 1597, 1527, 1469, 1383, 1320, 1257, 1176, 1100, 1032, 846 cm^−1^; ^1^H and ^13^C NMR data, see Table 1 and Table 2; HRESIMS *m*/*z* 308.1400 [M + H]^+^ (calcd for C_18_H_18_N_3_O_2_, 308.1399).

9-Ethoxy-8,9-dimethoxy-9*H*-benzo[*de*][1,6]naphthyridine (**3**): yellow solid; ${\left[\text{α}\right]}_{\text{D}}^{25}$ −7.16 (*c* 0.15, MeOH); UV (MeOH) (log ε) λ_max_ 202 (3.59), 229 (3.67), 352 (3.23); IR (KBr) ν_max_ 3064, 2928, 2855, 1716, 1673, 1641, 1570, 1490, 1458, 1413, 1371, 1349, 1305, 1276, 1223, 1201, 1176, 1075, 694, 917, 869, 656, 575 cm^−1^; ^1^H and ^13^C NMR data, see Table 1 and Table 2; HRESIMS *m*/*z* 295.1058 [M + Na]^+^ (calcd for C_15_H_16_N_2_O_3_Na, 295.1059). 

2-(*Sec*-butyl)-10-methoxyimidazo[4,5,1-*ij*]pyrido[2,3,4-*de*]quinolone (**4**): yellow solid; ${\left[\text{α}\right]}_{\text{D}}^{25}$ −15.98 (*c* 0.12, MeOH); UV (MeOH) (log ε) λ_max_ 210 (4.00), 225 (3.98), 239 (4.03), 272 (3.71), 360 (3.55); IR (KBr) ν_max_ 3315, 3065, 2960, 2926, 2854, 1731, 1688, 1607, 1573, 1515, 1465, 1419, 1366, 1307, 1279, 1167, 1020, 840, 799 cm^−1^; ^1^H and ^13^C NMR data, see Table 1 and Table 2; HRESIMS *m*/*z* 280.1452 [M + H]^+^ (calcd for C_17_H_18_N_3_O, 280.1450).

2-Isobutyl-10-methoxyimidazo[4,5,1-*ij*]pyrido[2,3,4-*de*]quinolone (**5**): yellow solid; UV (MeOH) (log ε) λ_max_ 211 (4.10), 225 (4.09), 239 (4.14), 272 (3.82), 361 (3.66); IR (KBr) ν_max_ 3342, 3050, 2957, 2925, 2869, 2853, 1729, 1688, 1607, 1572, 1542, 1515, 1465, 1420, 1348, 1324, 1307, 1279, 1194, 1167, 1020, 840 cm^−1^; ^1^H and ^13^C NMR data, see Table 1 and Table 2; HRESIMS *m*/*z* 280.1448 [M + H]^+^ (calcd for C_17_H_18_N_3_O, 280.1450).

### 3.4. Antifungal Evaluation

Concentration (MIC) values of compounds **3**–**8** were determined against six indicators (*C. parapsilosis*, *C. glabrata*, *C. albicans*, *C. neoformans*, *T. rubrum*, and *M. gypseum*), following the National Center for Clinical Laboratory Standards (NCCLS) methods [17,18]. Fluconazole was used as the positive control. Briefly, samples (dissolved in DMSO) were serially diluted in 20% DMSO/saline and transferred (10 μL) in duplicate to 96 well flat bottom microplates. Bacterial strains were grown aerobically at 0 °C in SDA for 16–20 h. A set of different concentrations of compounds **3**–**8** prepared in RPMI 1640 were next inoculated with the microorganisms and incubated 24 h for *C. parapsilosis*, and *C. glabrata* at 35 °C, 46 h for *C. albicans* at 35 °C, 72 h for *C. neoformans* at 35 °C, and 4–7 days for *T. rubrum* and *M. gypseum* at 30 °C. The MIC values were evaluated in triplicate for each compound. 

### 3.5. Anti-HIV-1 Activity Assay

The antiviral activity against HIV-1 was evaluated by using a cell-based VSVG/HIV-1 pseudotyping system as described [19,20]. For the infection assay, briefly, 293T cells were plated on 24-well plates at the density of 6 × 10^4^ cells per well one day prior to infection. Compounds with the concentrations of 10 μM were incubated with target cells for 15 min prior to adding VSVG/HIV-1. The same amount of solvent alone was used as blank control. After postinfection for 48 h, cells were lysed in 50 μL Cell Lysis Reagent (Promega Corp., Beijing, China). Luciferase activity of the cell lysate was measured by a FB15 luminometer (Berthold Detection Systems, Titertek-Berthold, Pforzheim, Germany).

## 4. Conclusions

Chemical investigation of the sponge *A. aaptos* from the South China Sea led to the isolation and structure elucidation of five new aaptamine derivatives (**1**–**5**), together with three related known aaptamines (**6**–**8**), demonstrating an excellent example of chemical diversity. Structurally, compounds **1**–**2** possesses an uncommon triazapyrene lactam fused to aaptamine moiety, compounds **4**–**5** share an imidazole-fused aaptamine skeleton. Unfortunately, due to the lack of compounds **1** and **2**, the bioactivity of **1**–**2** could not be evaluated. These compounds displayed different levels of *in vitro* antifungal and anti-HIV-1 activities. Especially, according to our research, compounds **7** and **8** exhibited better antifungal and anti-HIV-1 activities than the rest compounds.

## Supplementary Files

Supplementary File 1
