# Supplementary material for: Aaptamine Derivatives with Antifungal and Anti-HIV-1 Activities from the South China Sea Sponge Aaptos aaptos
Source: Mar Drugs. 2014 Dec 16;12(12):6003–13. doi: 10.3390/md12126003 (PMC4278215; doi:10.3390/md12126003)
Supplement: Supplementary File 1 [file marinedrugs-12-06003-s001.pdf]

Supplementary Information

Figure S1 ^{1}H NMR spectrum of compound **1** in DMSO-*d*$_6$
Figure S2 ^{13}C NMR spectrum of compound **1** in DMSO-*d*$_6$
Figure S3 DEPT135 Spectrum of compound **1** in DMSO-*d*$_6$
Figure S4 HSQC spectrum of compound **1** in DMSO-*d*$_6$
Figure S5 HMBC spectrum of compound **1** in DMSO-*d*$_6$
Figure S6 ^{1}H-^{1}H COSY spectrum of compound **1** in DMSO-*d*$_6$
Figure S7 NOESY spectrum of compound **1** in DMSO-*d*$_6$
Figure S8 IR spectrum of compound **1**
Figure S9 HRESIMS of compound **1**
Figure S10 UV spectrum of compound **1**
Figure S11 ^{1}H NMR spectrum of compound **2** in DMSO-*d*$_6$
Figure S12 ^{13}C NMR spectrum of compound **2** in DMSO-*d*$_6$
Figure S13 DEPT135 Spectrum of compound **2** in DMSO-*d*$_6$
Figure S14 HSQC spectrum of compound **2** in DMSO-*d*$_6$
Figure S15 HMBC spectrum of compound **2** in DMSO-*d*$_6$
Figure S16 ^{1}H-^{1}H COSY spectrum of compound **2** in DMSO-*d*$_6$
Figure S17 NOESY spectrum of compound **2** in DMSO-*d*$_6$
Figure S18 IR spectrum of compound **2**
Figure S19 HRESIMS of compound **2**
Figure S20 UV spectrum of compound **2**
Figure S21 ^{1}H NMR spectrum of compound **3** in $CDCl_3$
Figure S22 ^{13}C NMR spectrum of compound **3** in $CDCl_3$
Figure S23 DEPT135 Spectrum of compound **3** in $CDCl_3$
Figure S24 HSQC spectrum of compound **3** in $CDCl_3$
Figure S25 HMBC spectrum of compound **3** in $CDCl_3$
Figure S26 ^{1}H-^{1}H COSY spectrum of compound **3** in $CDCl_3$
Figure S27 NOESY spectrum of compound **3** in $CDCl_3$
Figure S28 IR spectrum of compound **3**
Figure S29 HRESIMS of compound **3**
Figure S30 UV spectrum of compound **3**
Figure S31 ^{1}H NMR spectrum of compound **4** in $CDCl_3$
Figure S32 ^{13}C NMR spectrum of compound **4** in $CDCl_3$
Figure S33 DEPT135 Spectrum of compound **4** in $CDCl_3$
Figure S34 HSQC spectrum of compound **4** in $CDCl_3$
Figure S35 HMBC spectrum of compound **4** in $CDCl_3$
Figure S36 ^{1}H-^{1}H COSY spectrum of compound **4** in $CDCl_3$
Figure S37 NOESY spectrum of compound **4** in $CDCl_3$
Figure S38 IR spectrum of compound **4**
Figure S39 HRESIMS of compound **4**
Figure S40 UV spectrum of compound **4**
Figure S41 ^{1}H NMR spectrum of compound **5** in $CDCl_3$

Figure S42 ^{13}C NMR spectrum of compound **5** in $CDCl_3$

Figure S43 DEPT135 Spectrum of compound **5** in $CDCl_3$

Figure S44 HSQC spectrum of compound **5** in $CDCl_3$

Figure S45 HMBC spectrum of compound **5** in $CDCl_3$

Figure S46 ^{1}H-^{1}H COSY spectrum of compound **5** in $CDCl_3$

Figure S47 NOESY spectrum of compound **5** in $CDCl_3$

Figure S48 IR spectrum of compound **5**

Figure S49 HRESIMS of compound **5**

Figure S50 UV spectrum of compound **5**

Figure S51 ^{1}H NMR spectrum of compound **6** in $CDCl_3$

Figure S52 ^{13}C NMR spectrum of compound **6** in $CDCl_3$

Figure S53 ^{1}H NMR spectrum of compound **7** in $CDCl_3$

Figure S54 ^{13}C NMR spectrum of compound **7** in $CDCl_3$

Figure S55 ^{1}H NMR spectrum of compound **8** in $CDCl_3$

Figure S56 ^{13}C NMR spectrum of compound **8** in $CDCl_3$

Table S1 NMR spectroscopic data for compounds **6**–**8** in $CDCl_3$

Figure S1. ^{1}H NMR spectrum of compound **1** in DMSO-*d*6.

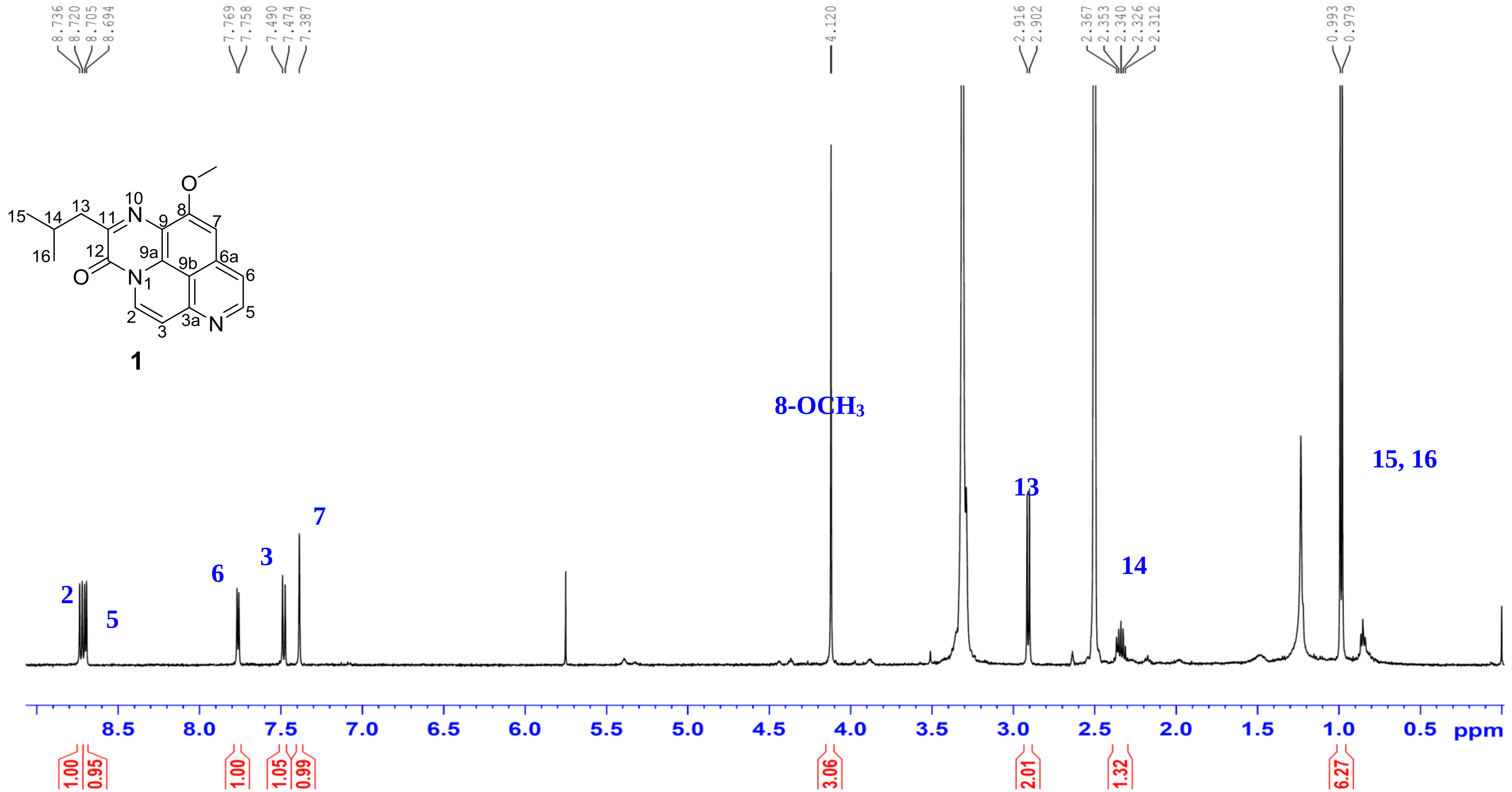

Figure S2. ^{13}C NMR spectrum of compound **1** in DMSO-d_6.

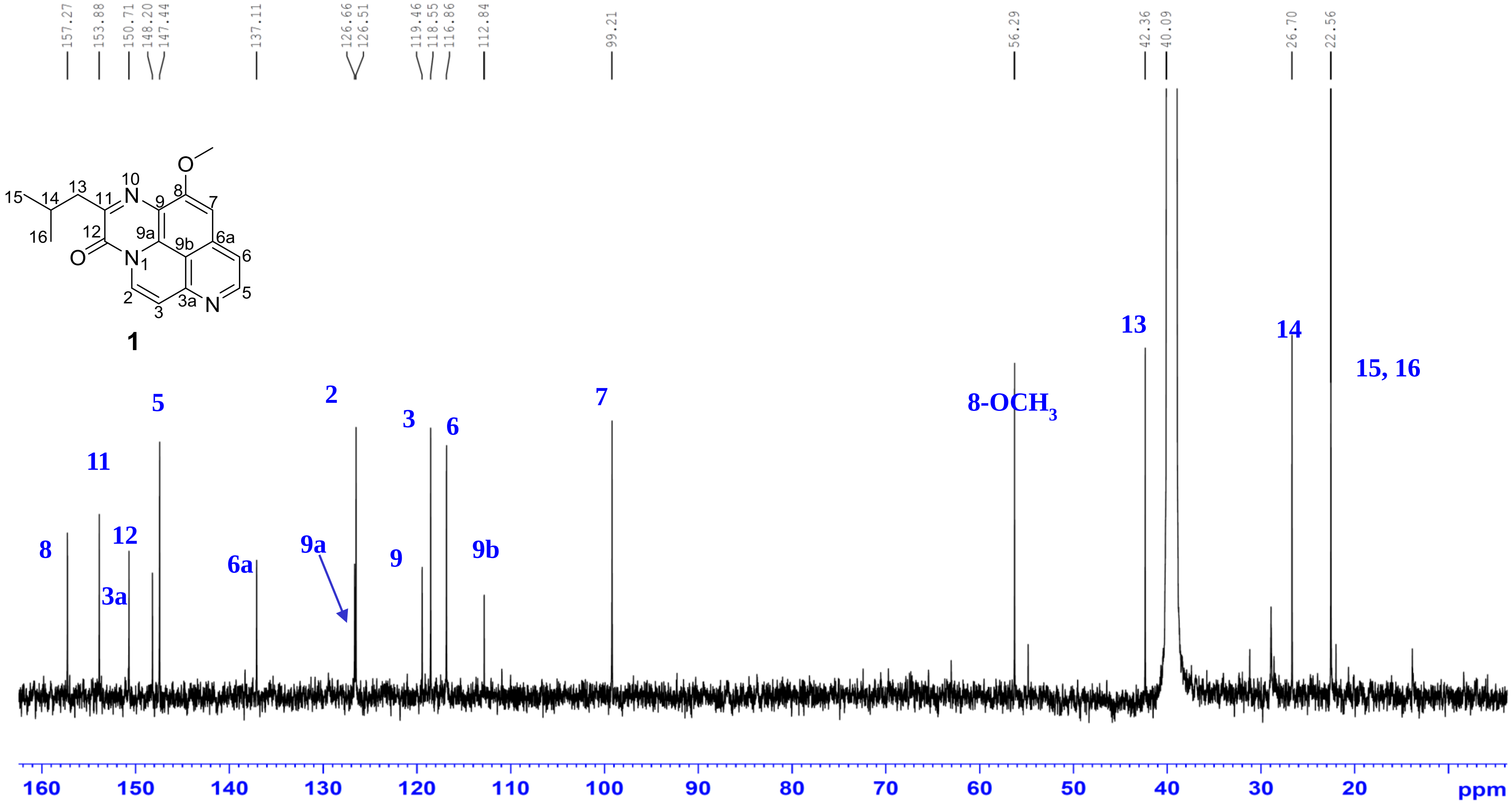

Figure S3. DEPT135 Spectrum of compound **1** in DMSO-d_6.

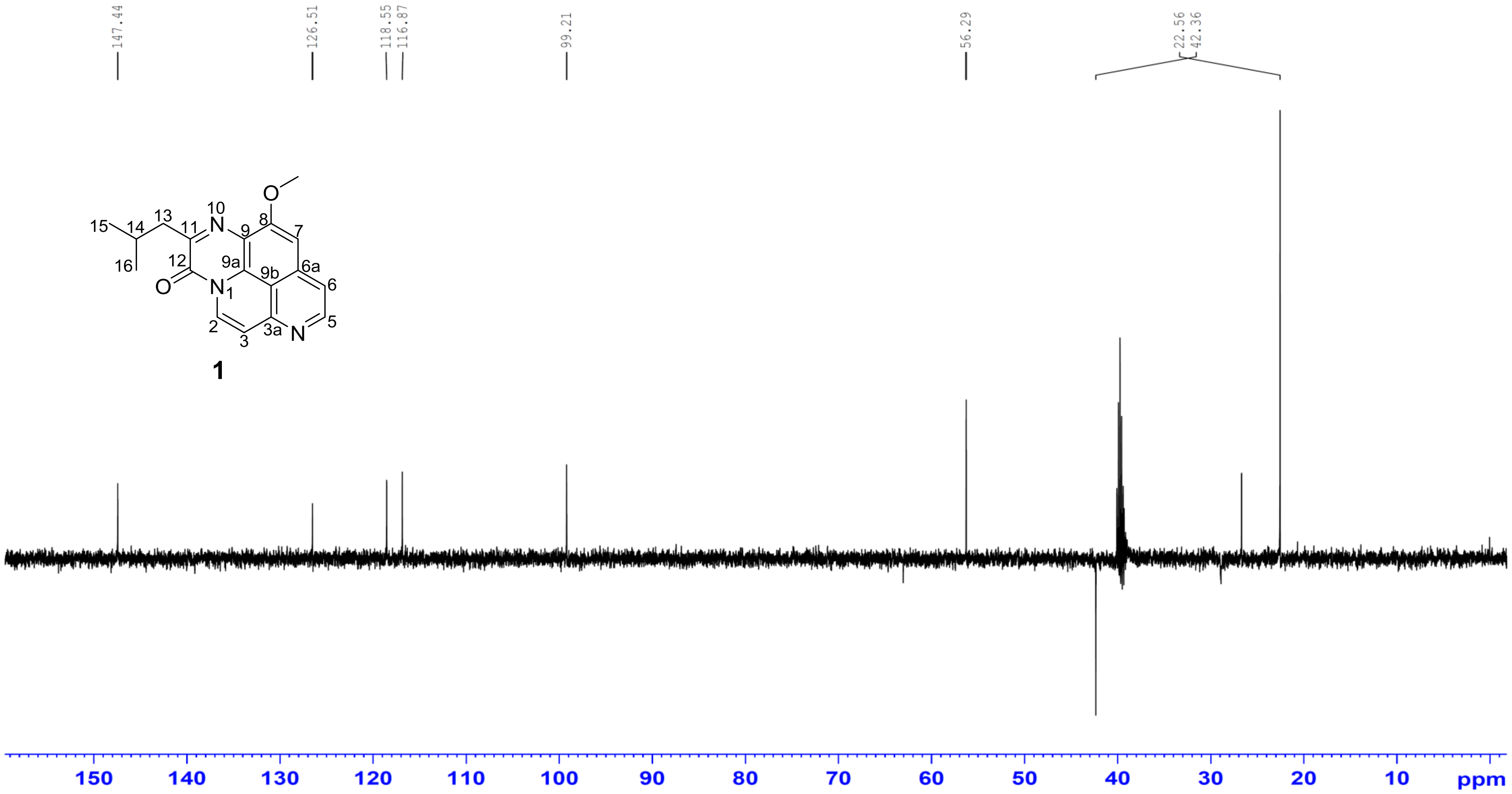

Figure S4. HSQC spectrum of compound **1** in DMSO-d_6.

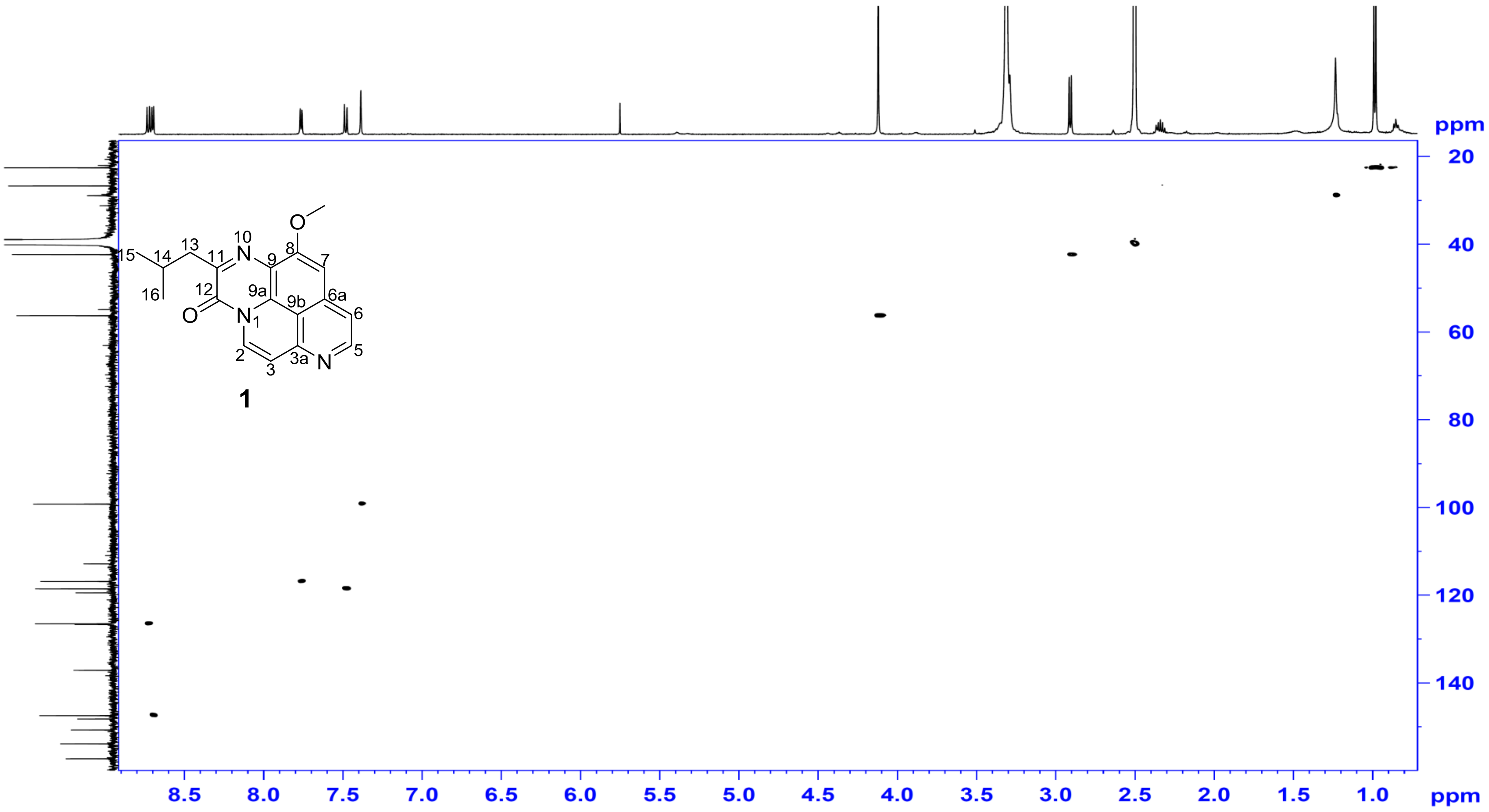

Figure S5. HMBC spectrum of compound **1** in DMSO-d_6.

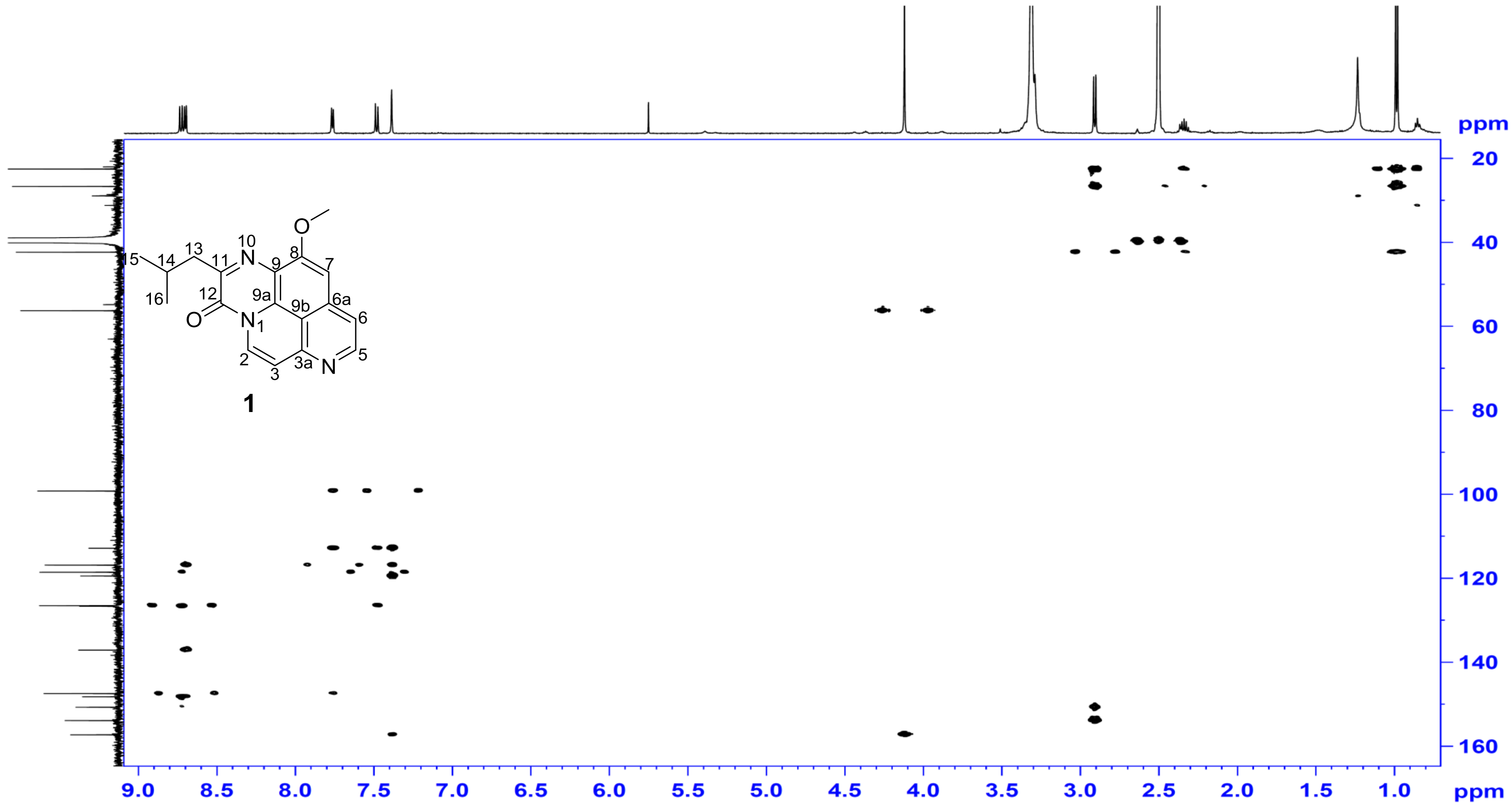

Figure S6. ¹H-¹H COSY spectrum of compound **1** in DMSO-d_6.

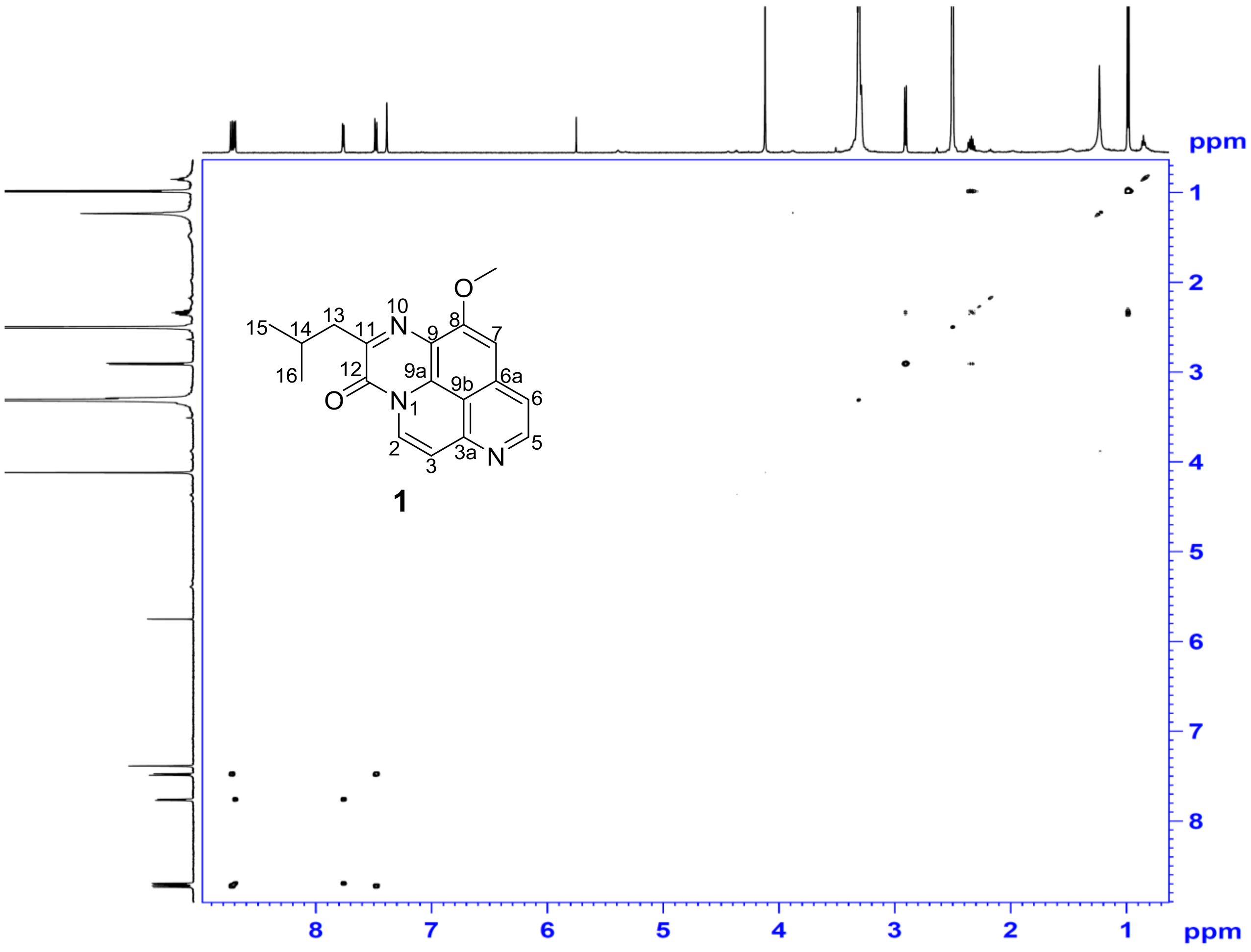

Figure S7. NOESY spectrum of compound **1** in DMSO-d_6.

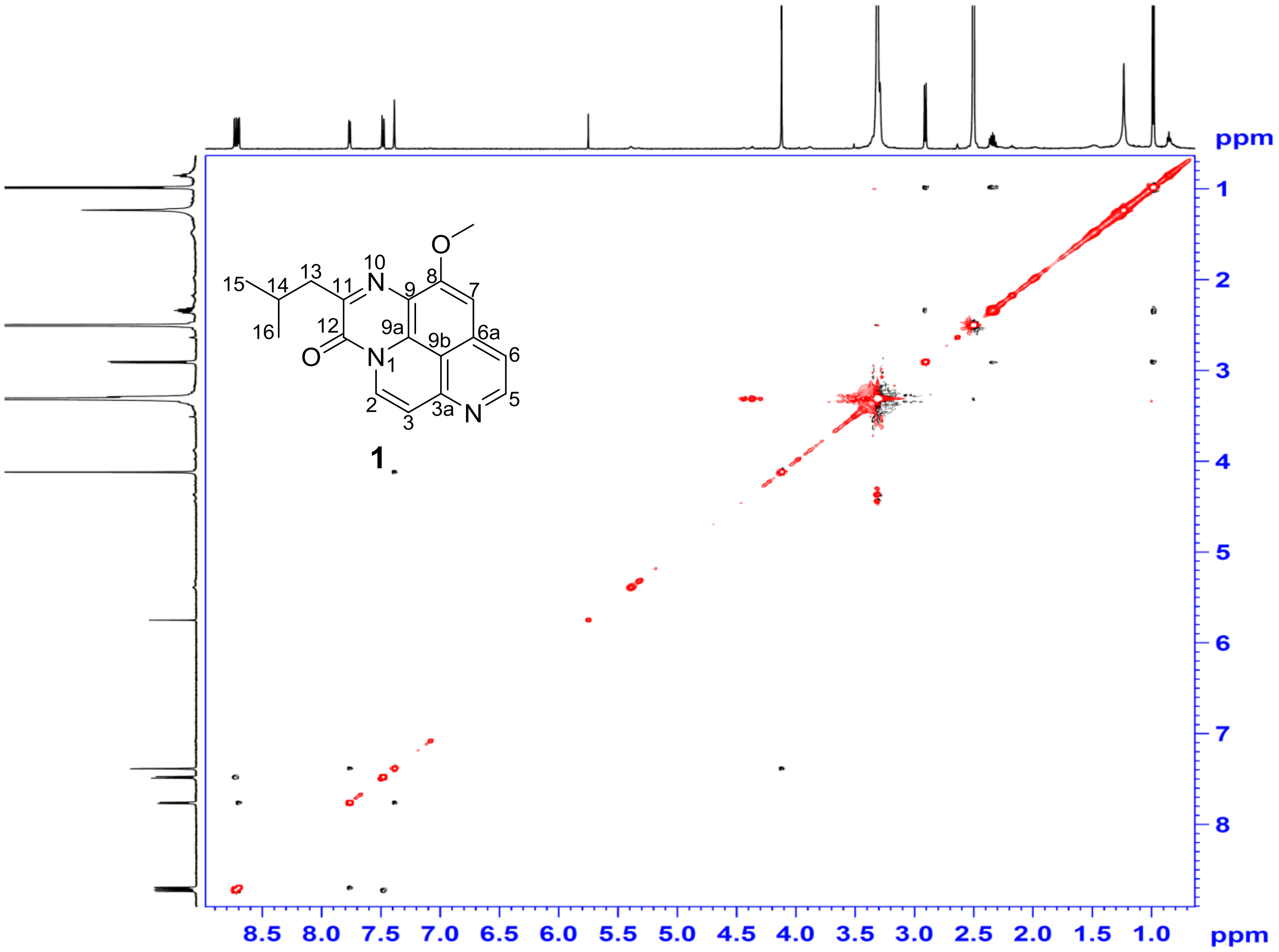

Figure S8. IR spectrum of compound **1**.

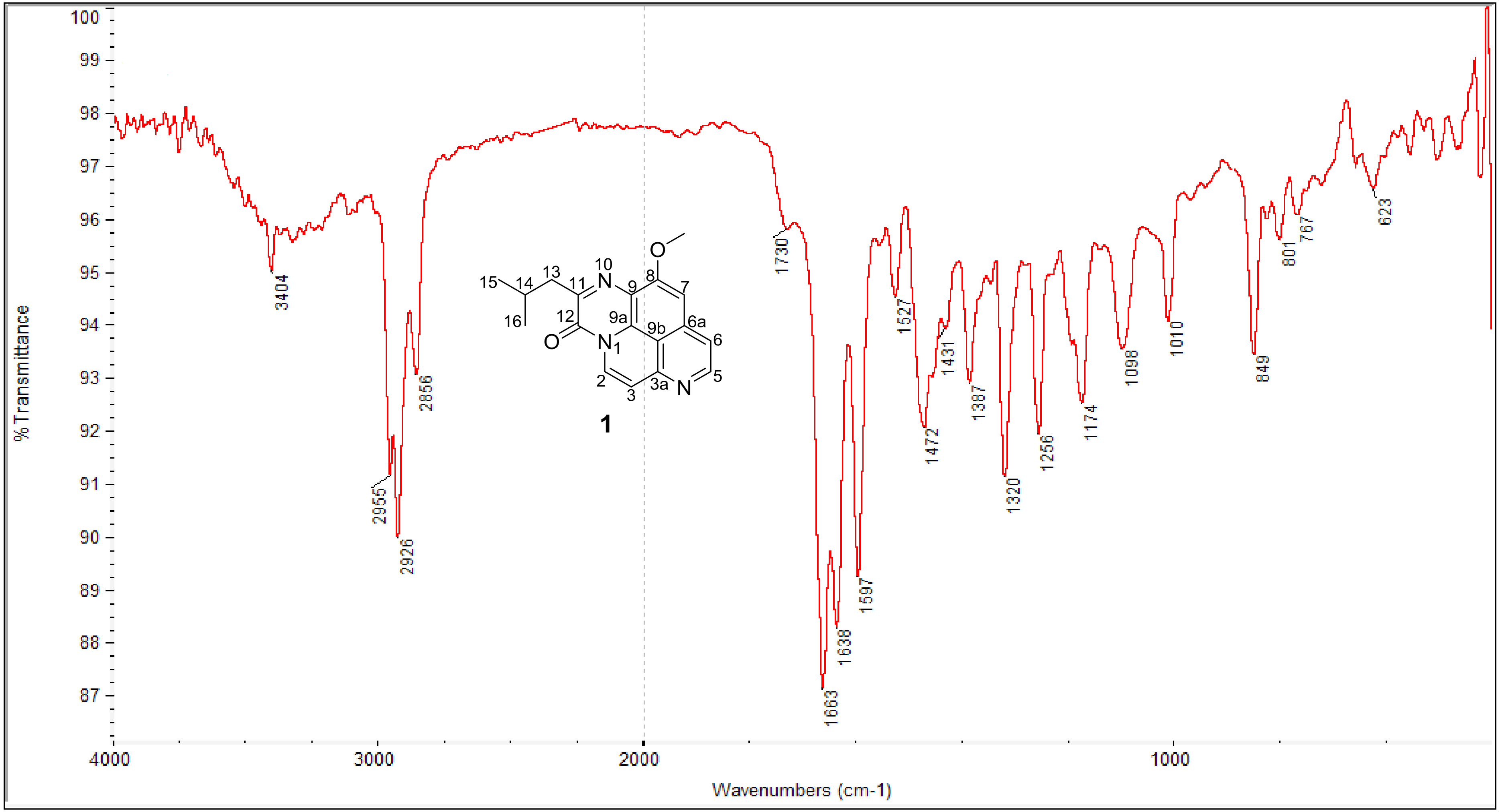

Figure S9. HRESIMS of compound **1**.

Elemental Composition Report

Page 1

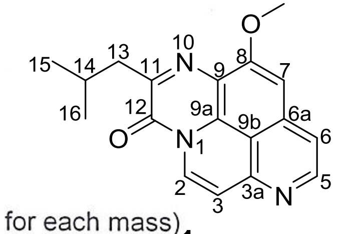

1

Tolerance = 50.0 PPM / DBE: min = -1.5, max = 50.0
Selected filters: None

Monoisotopic Mass, Even Electron Ions
8 formula(e) evaluated with 1 results within limits (up to 50 closest results for each mass)
Elements Used:
C: 5-20 H: 5-25 N: 1-3 O: 1-2 Na: 1-1

SIPI
M.W=307
Q-Tof micro
YA019
22-Jul-2014,15:21:17
WQ14-219H2 15 (0.517) AM (Cen,4, 80.00, Ar,5000.0,335.13,0.70); Sm (SG, 2x3.00); Cm (10:19)
TOF MS ES+
1.02e3

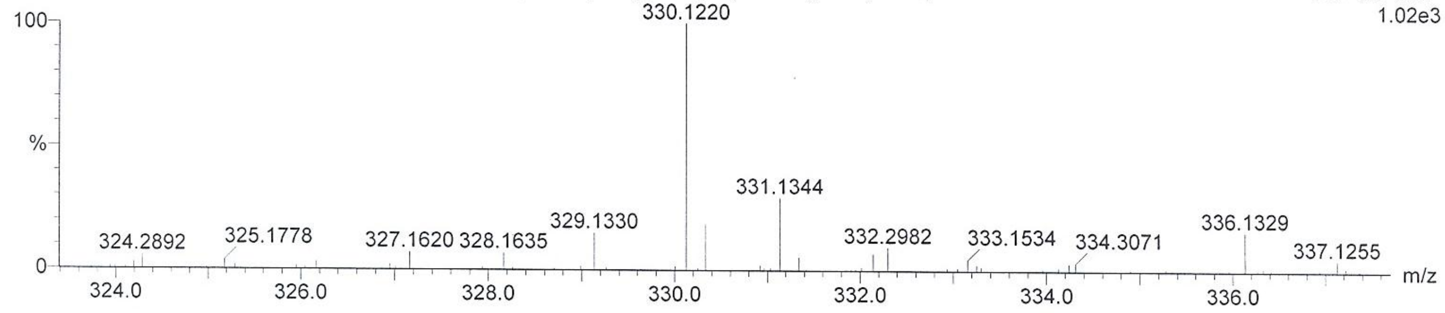

Minimum:	60.00				-1.5		
Maximum:	100.00		5.0	50.0	50.0		
Mass	RA	Calc. Mass	mDa	PPM	DBE	i-FIT	Formula
330.1220	100.00	330.1218	0.2	0.6	11.5	22.0	C18 H17 N3 O2 Na

Figure S10. UV spectrum of compound **1**.

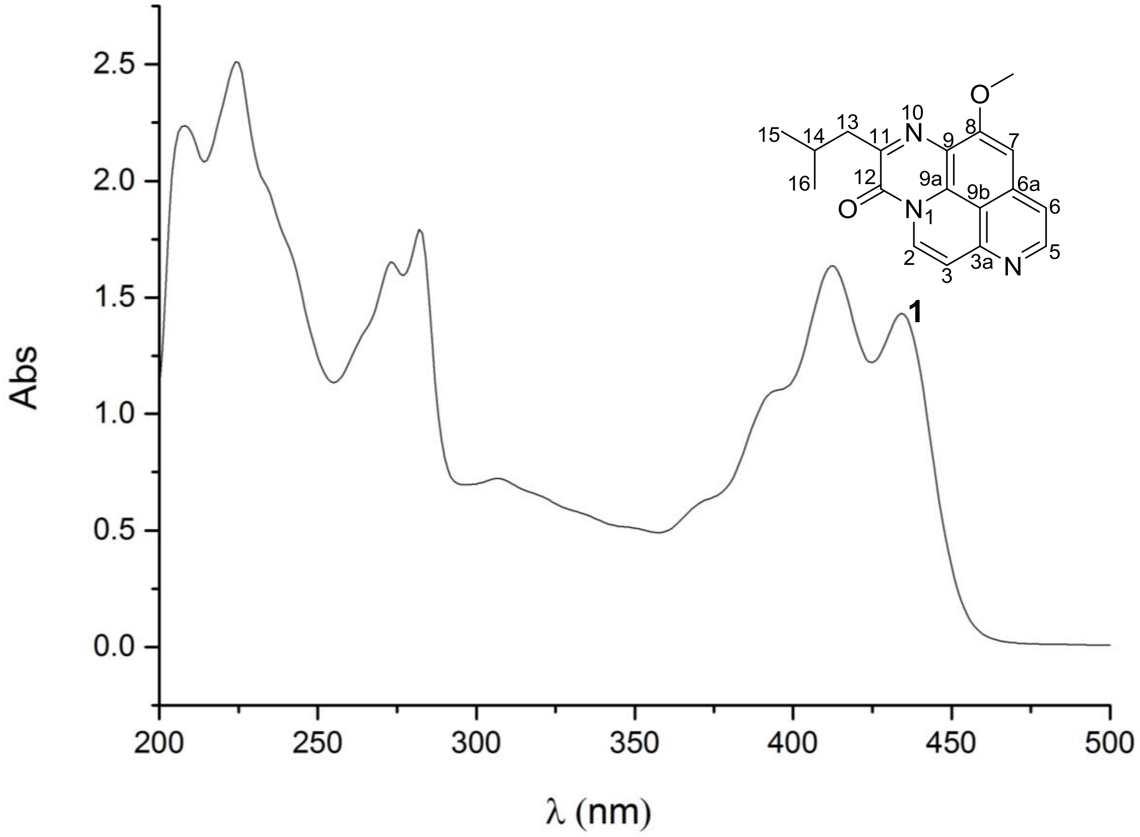

Figure S11. ^{1}H NMR spectrum of compound **2** in DMSO-d_6.

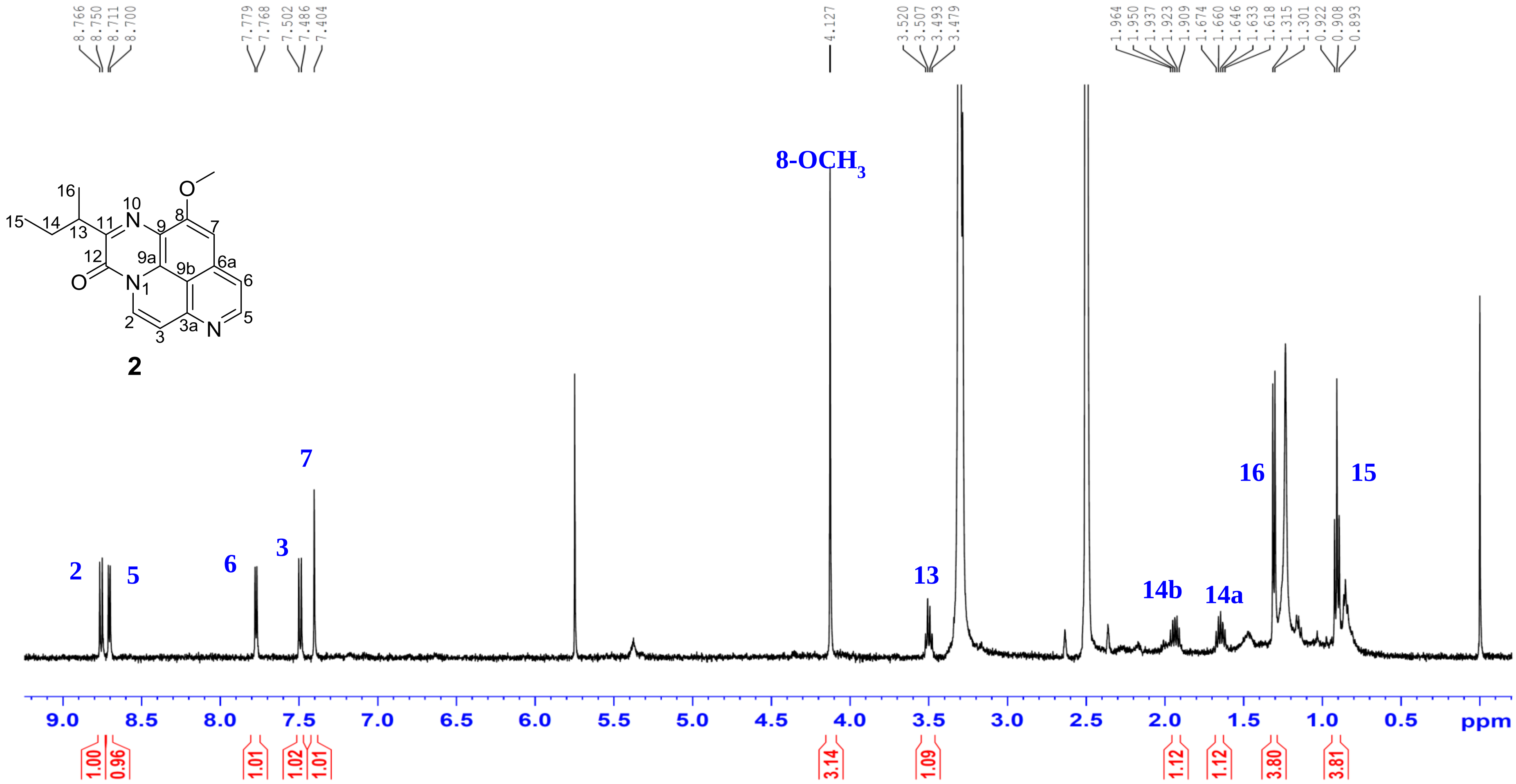

Figure S12. ^{13}C NMR spectrum of compound **2** in DMSO-*d*6.

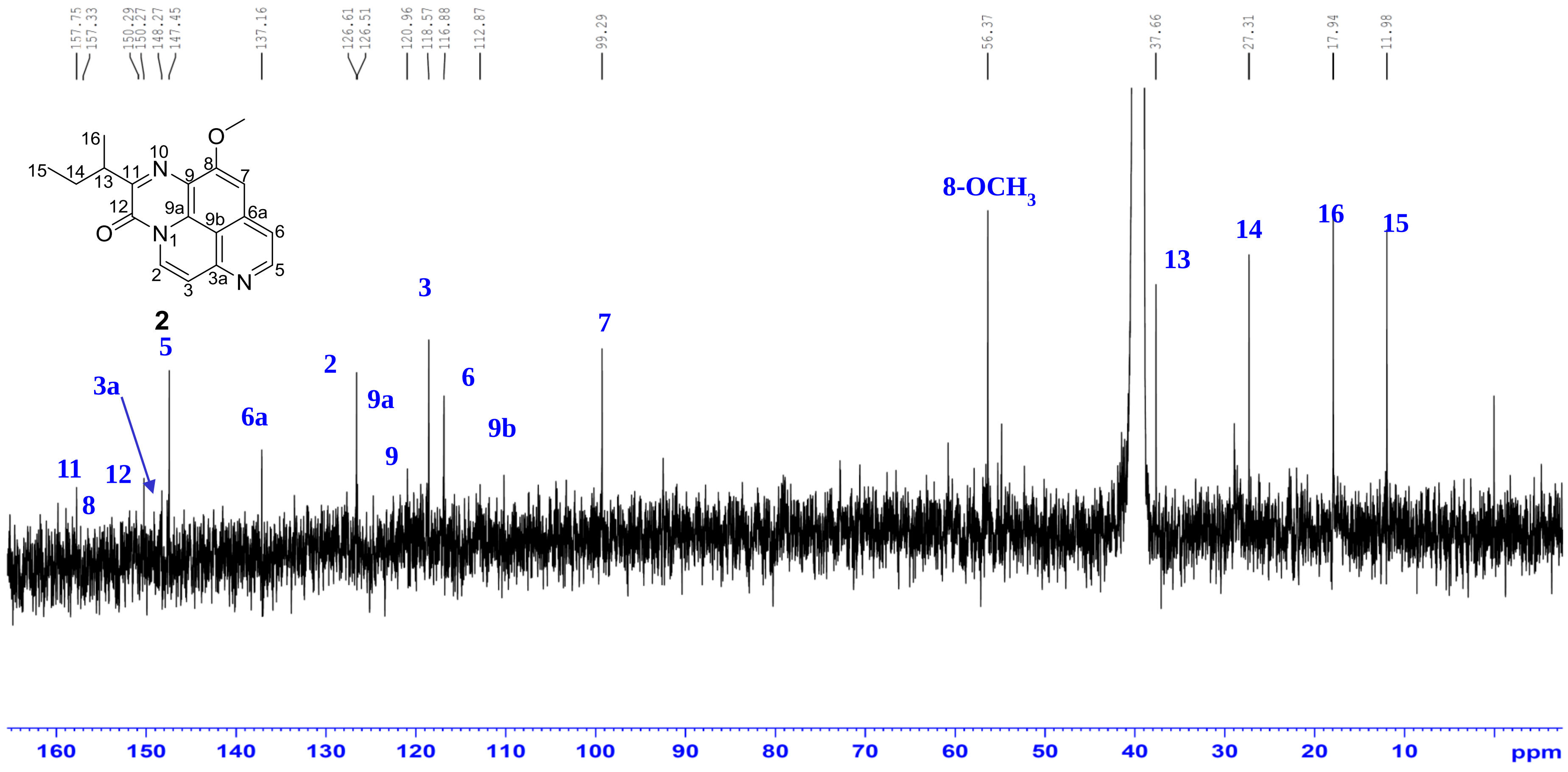

Figure S13. DEPT135 Spectrum of compound **2** in DMSO-d_6.

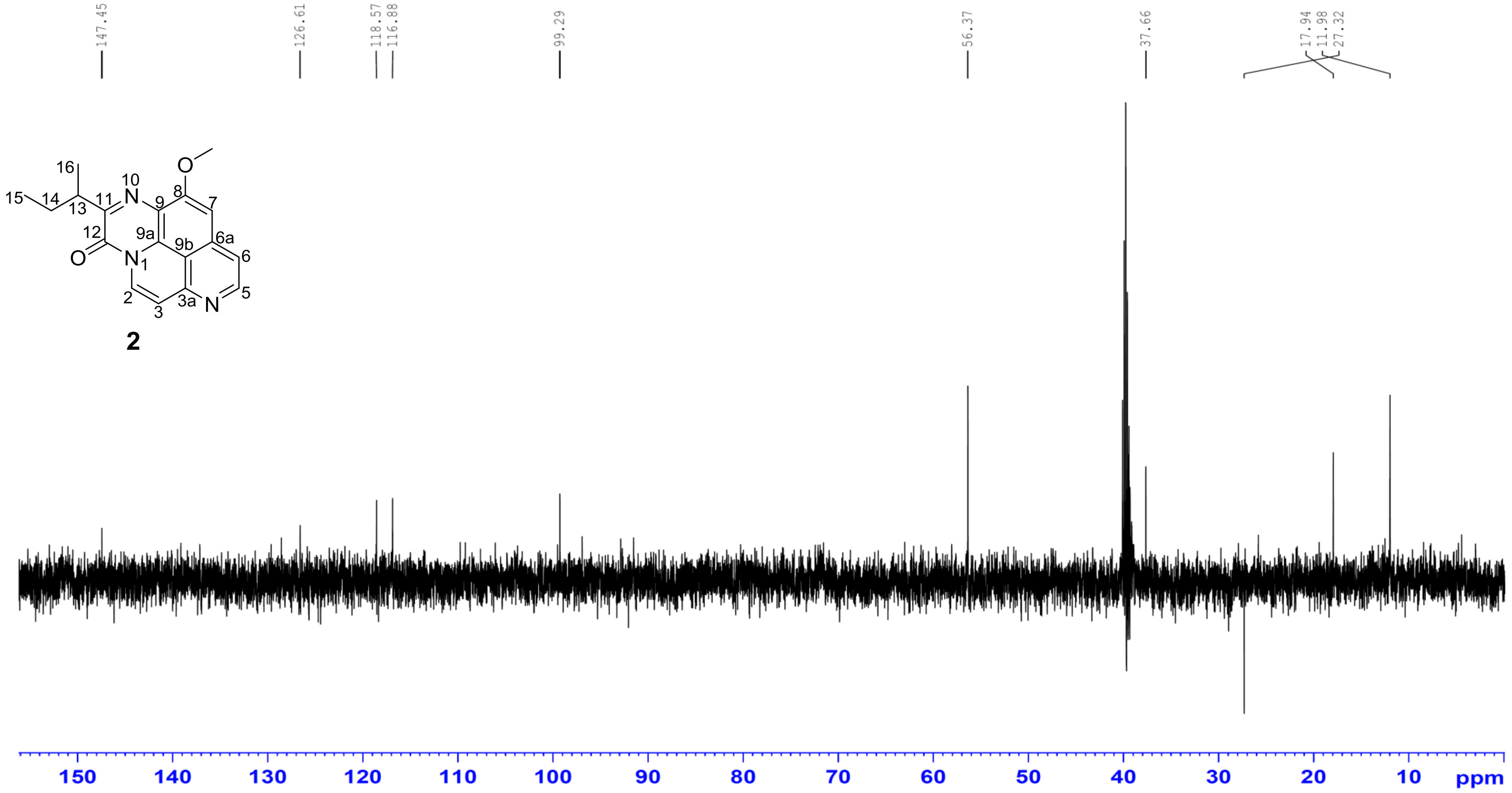

Figure S14. HSQC spectrum of compound **2** in DMSO-d_6.

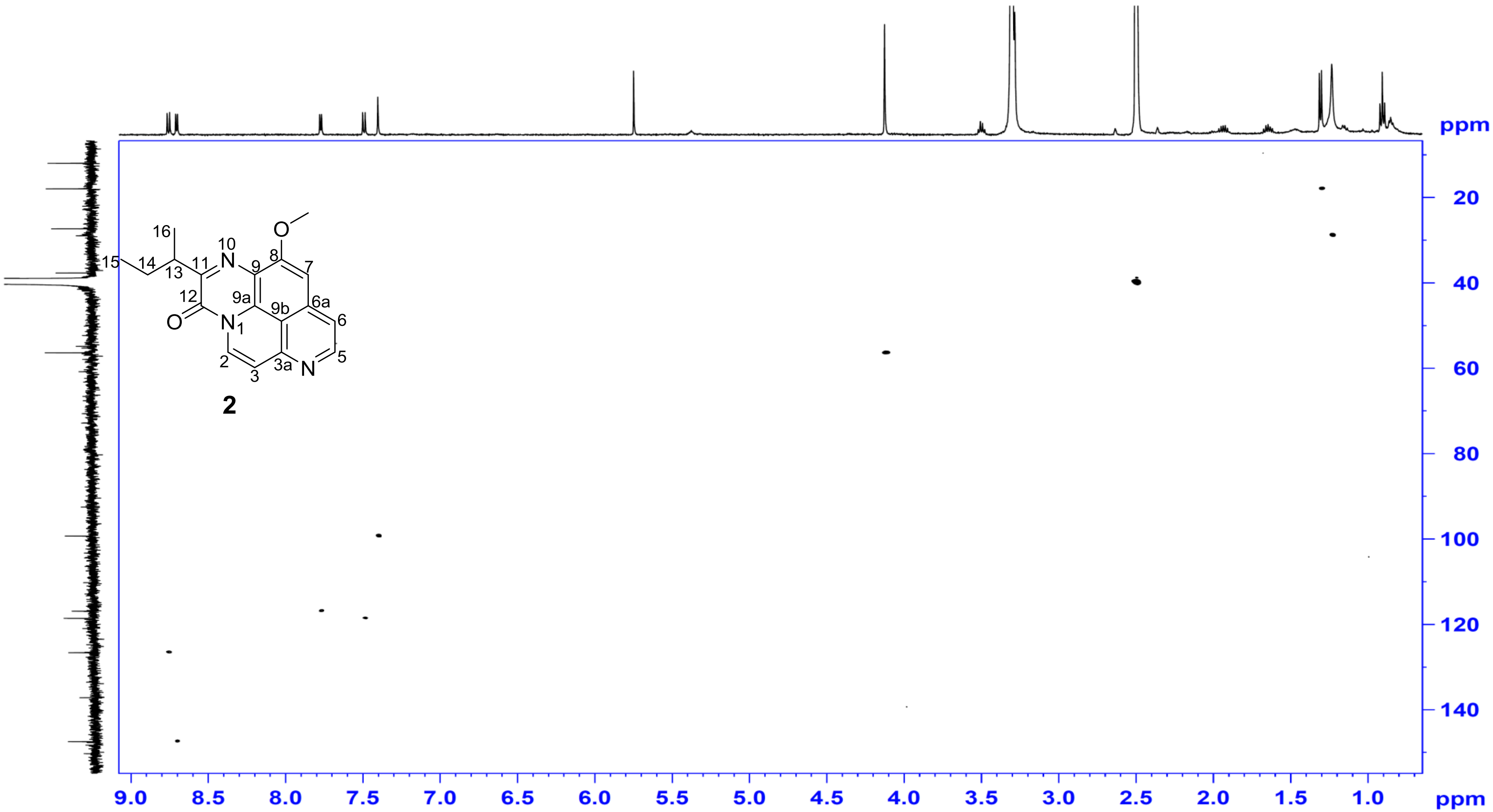

Figure S15. HMBC spectrum of compound **2** in DMSO-d_6.

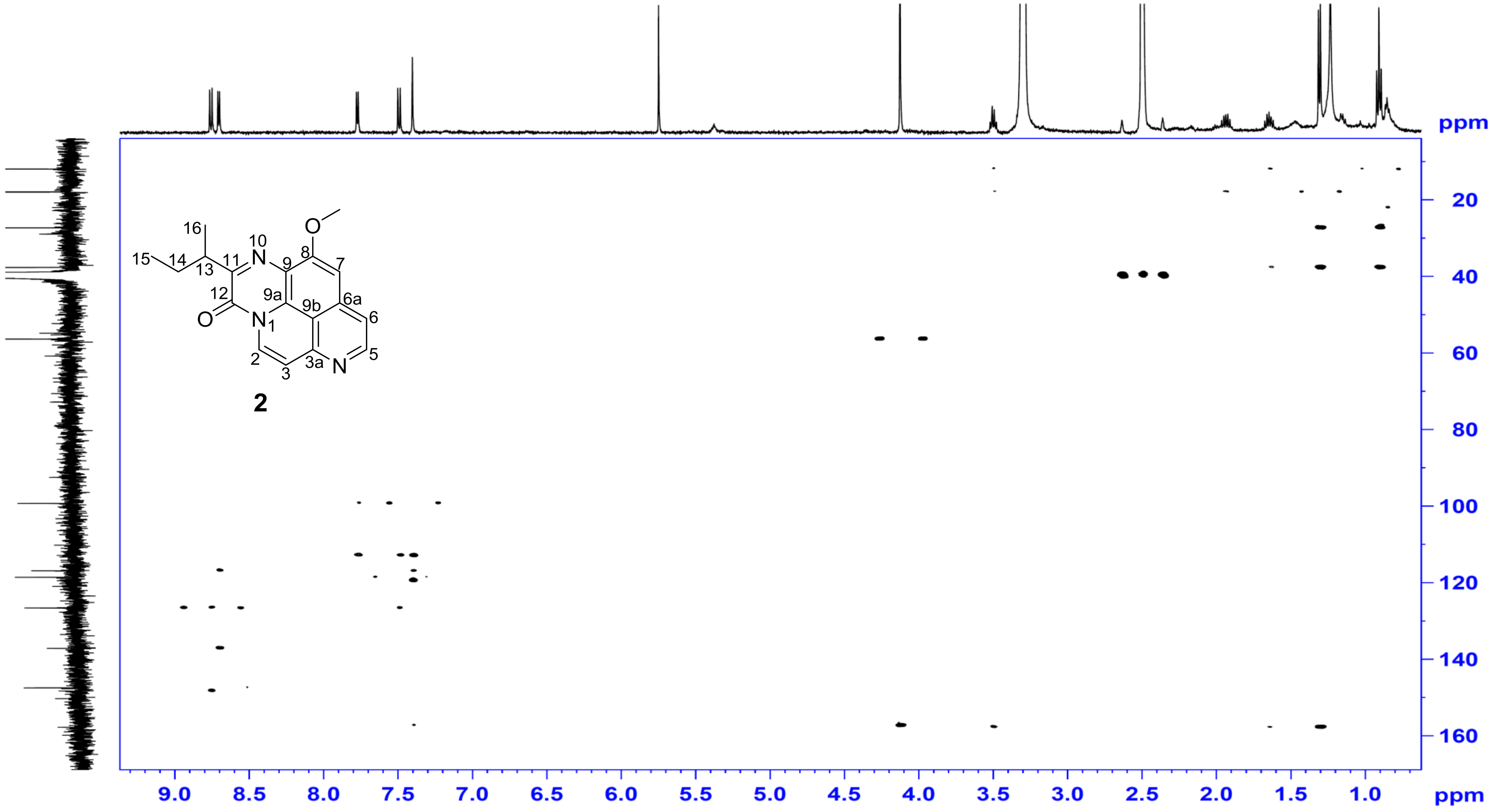

Figure S16. ^{1}H-^{1}H COSY spectrum of compound **2** in DMSO-*d*6.

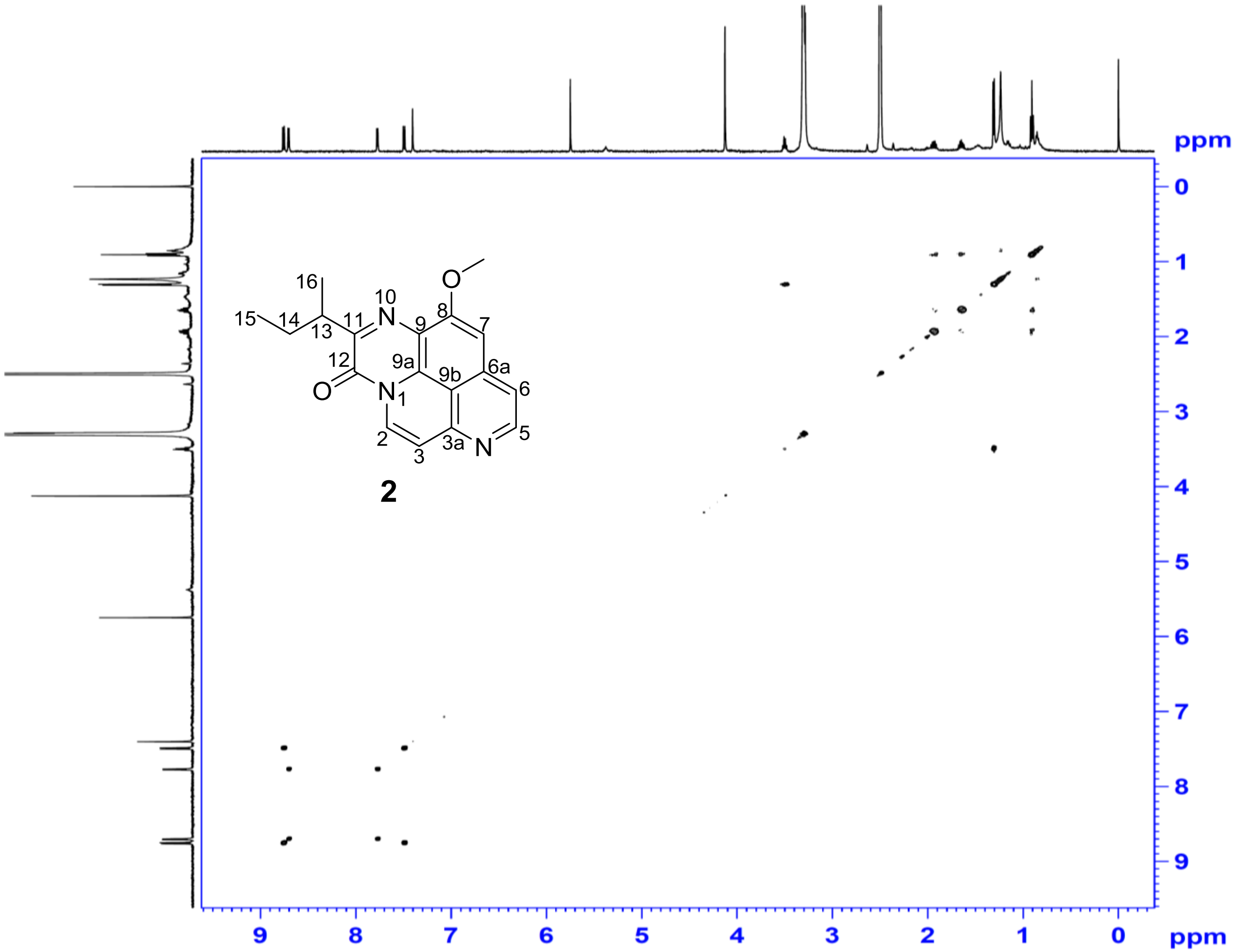

Figure S17. NOESY spectrum of compound **2** in DMSO-d_6.

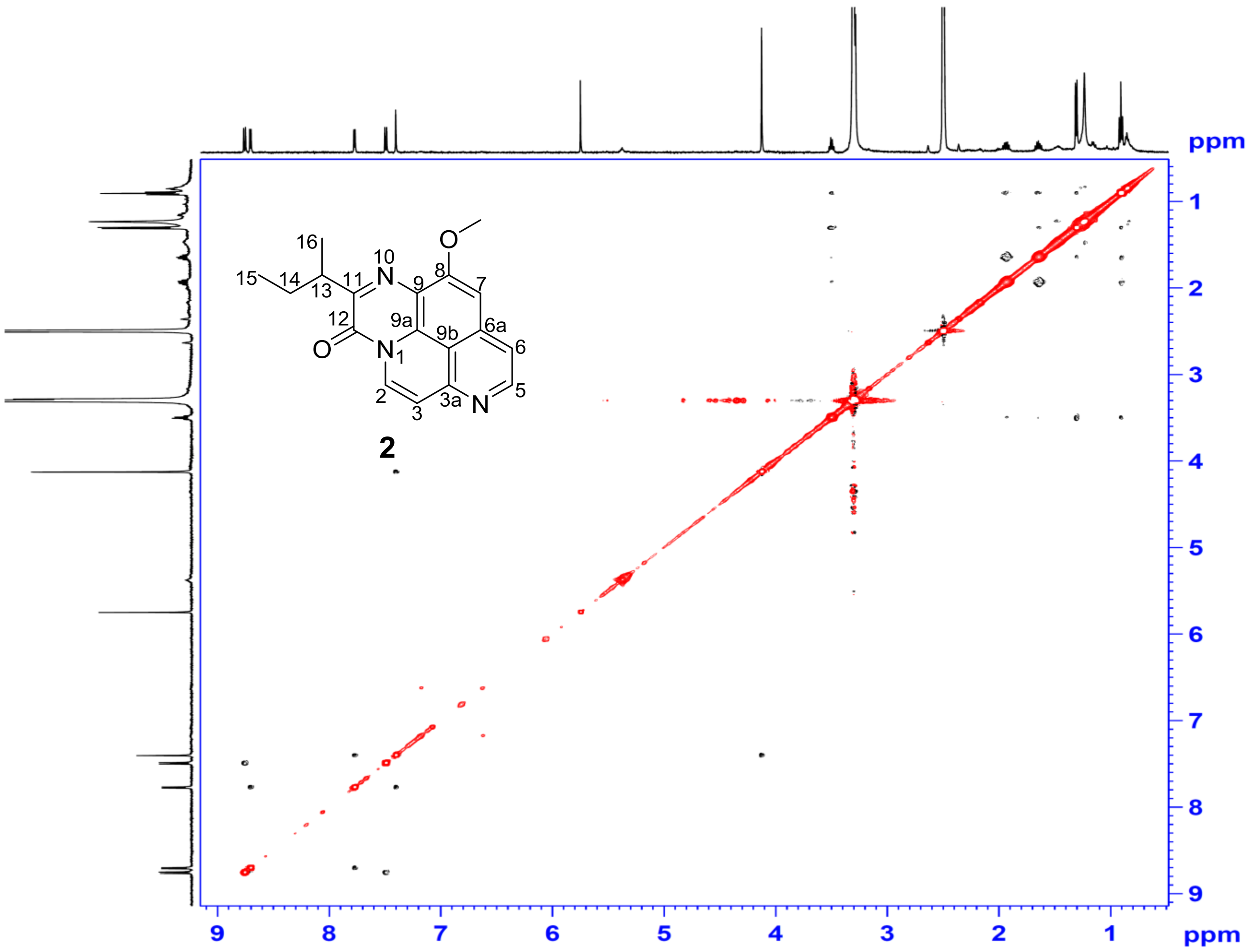

Figure S18. IR spectrum of compound **2**.

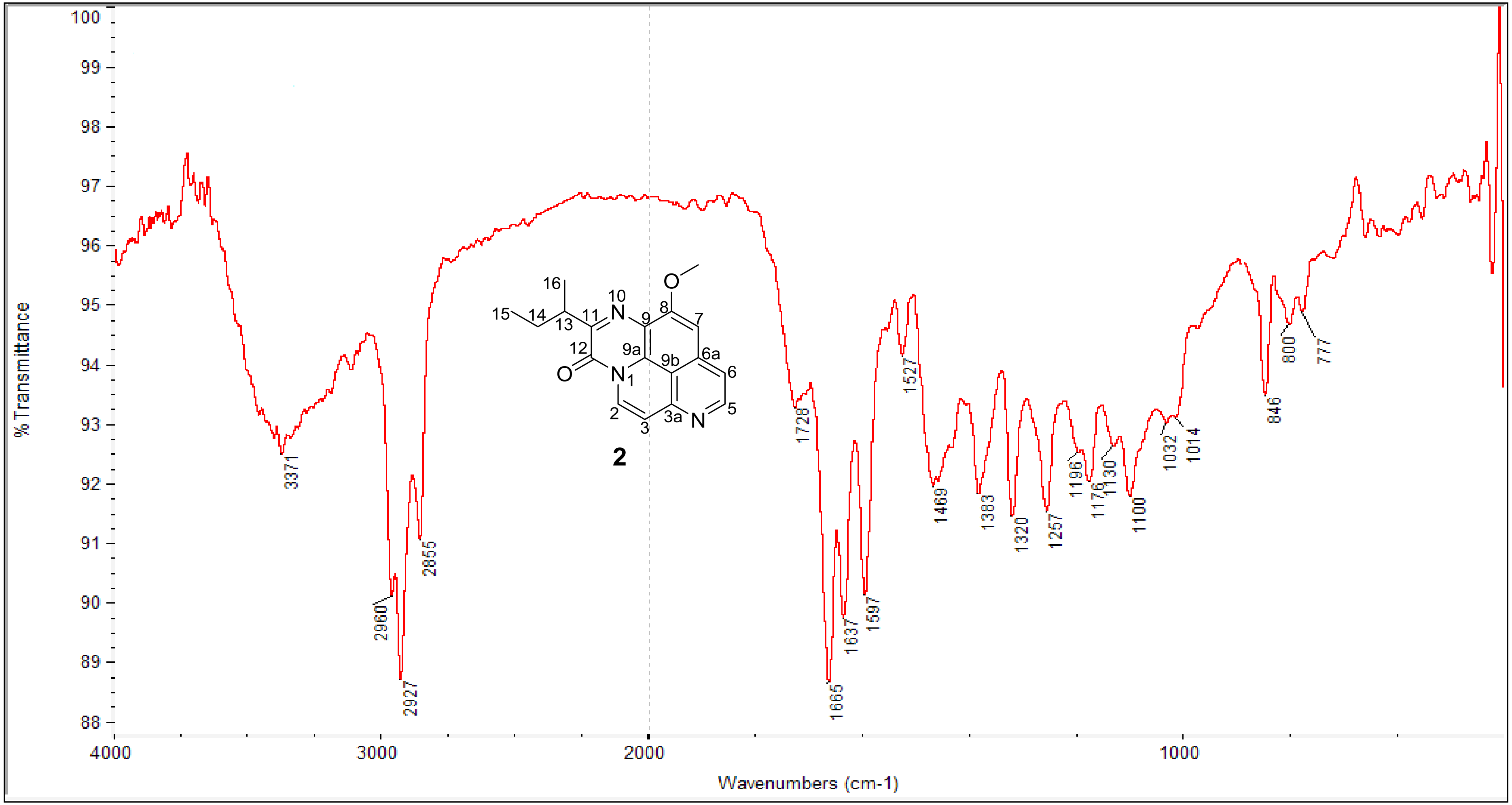

Figure S19. HRESIMS of compound **2**.

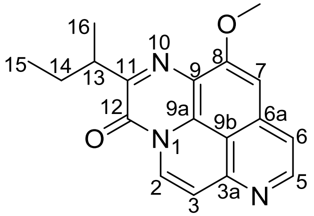

2

Elemental Composition Report

Page 1

Tolerance = 50.0 PPM / DBE: min = -1.5, max = 50.0
Selected filters: None

Monoisotopic Mass, Even Electron Ions
8 formula(e) evaluated with 1 results within limits (up to 50 closest results for each mass)
Elements Used:
C: 5-20 H: 5-25 N: 1-3 O: 1-2

SIPI Q-Tof micro 22-Jul-2014,15:27:52
M.W=307 YA019
WQ14-220H 27 (0.934) AM (Cen,4, 80.00, Ar,5000.0,313.15,0.70); Sm (SG, 2x3.00); Cm (21:29) TOF MS ES+
4.03e3

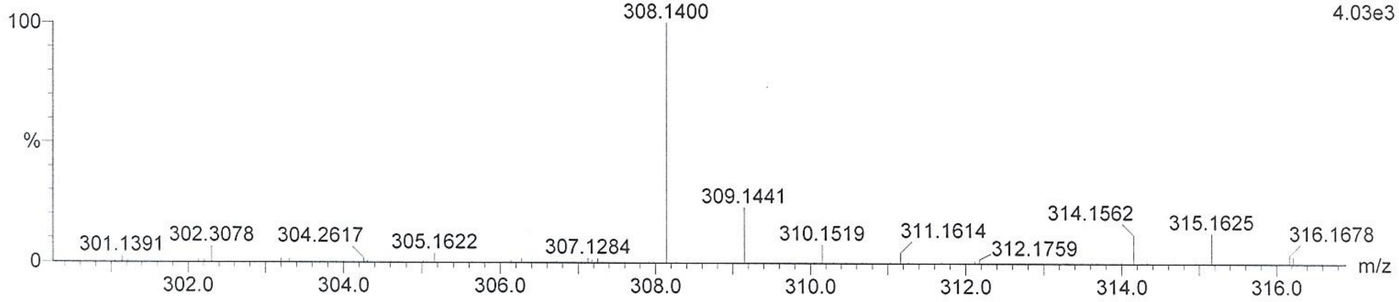

Minimum:	60.00				-1.5		
Maximum:	100.00		5.0	50.0	50.0		
Mass	RA	Calc. Mass	mDa	PPM	DBE	i-FIT	Formula
308.1400	100.00	308.1399	0.1	0.3	11.5	64.9	C18 H18 N3 O2

Figure S20. UV spectrum of compound **2**.

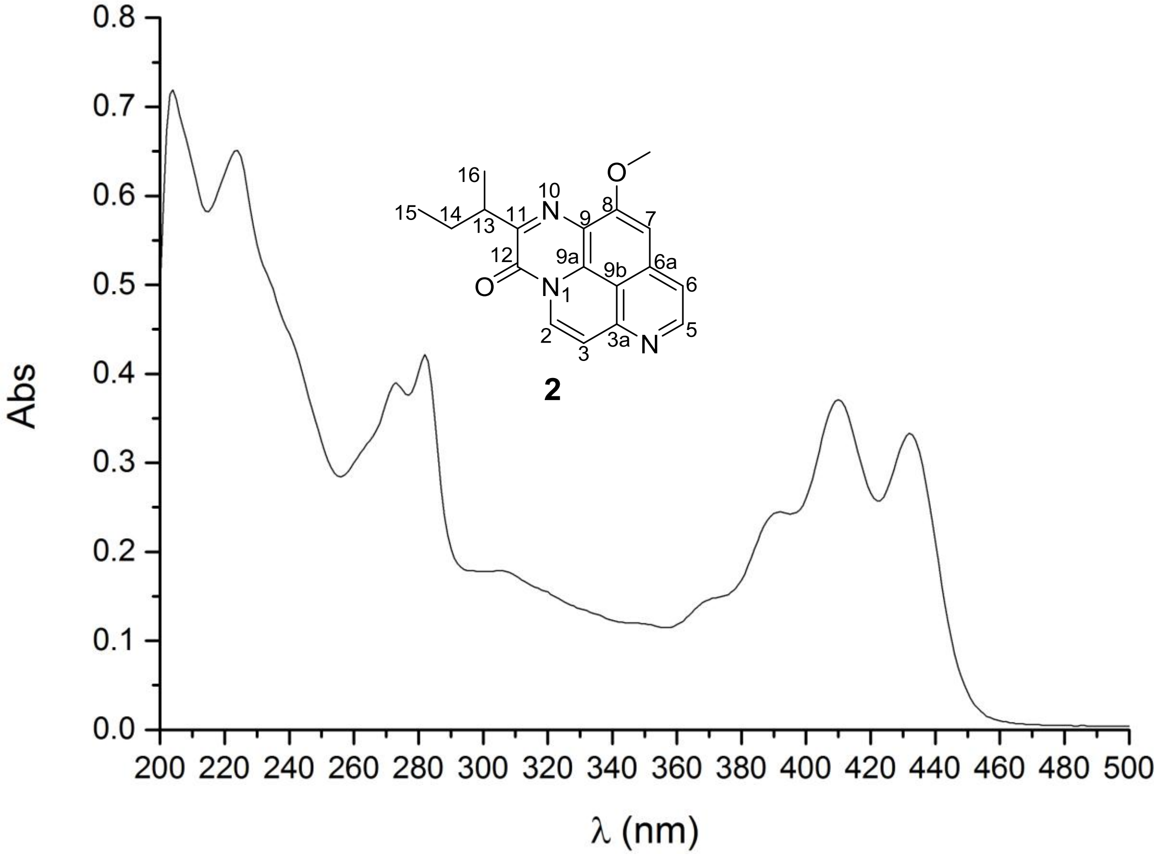

Figure S21. ^{1}H NMR spectrum of compound **3** in $CDCl_3$.

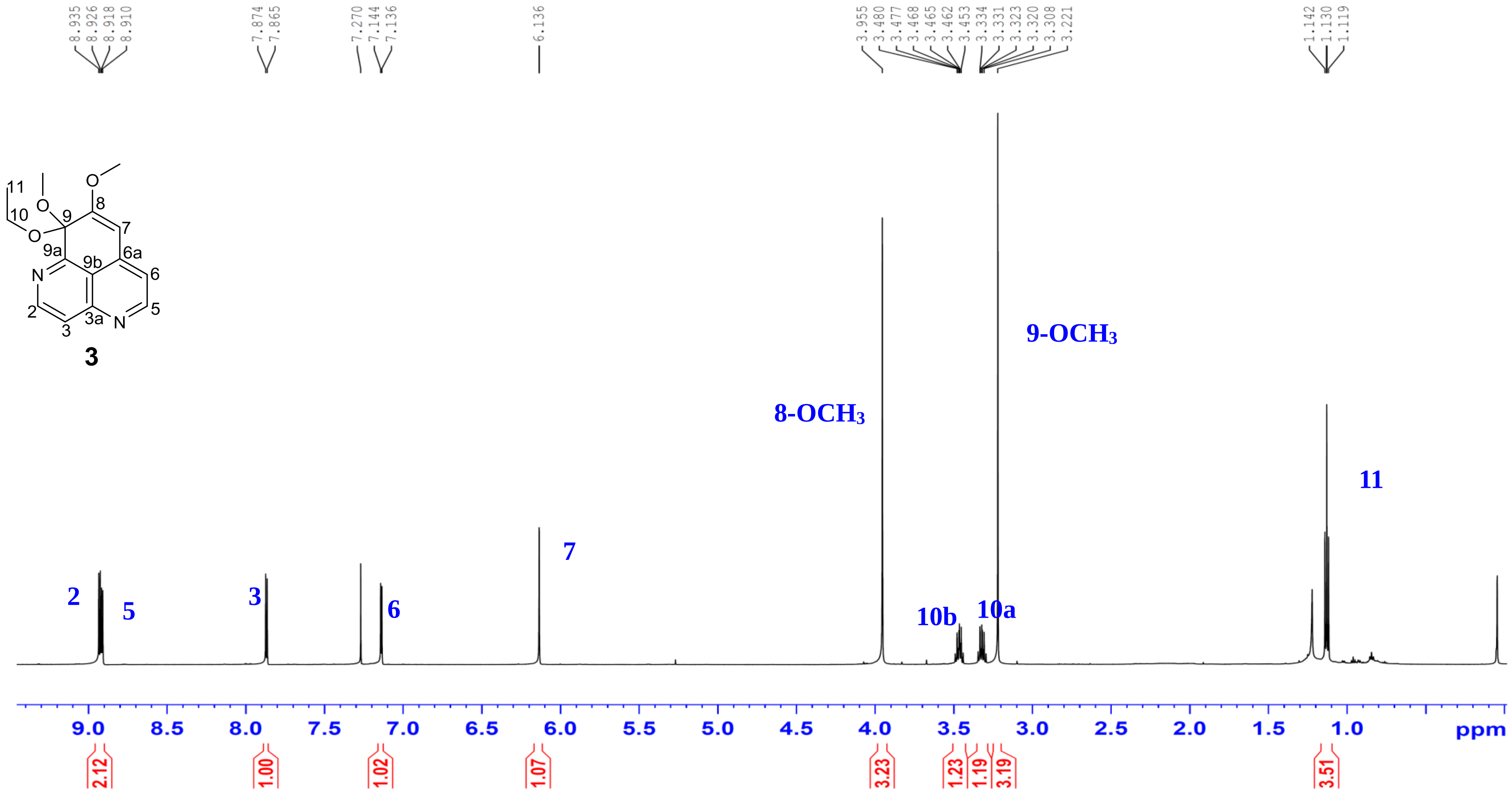

Figure S22. ^{13}C NMR spectrum of compound **3** in $CDCl_3$.

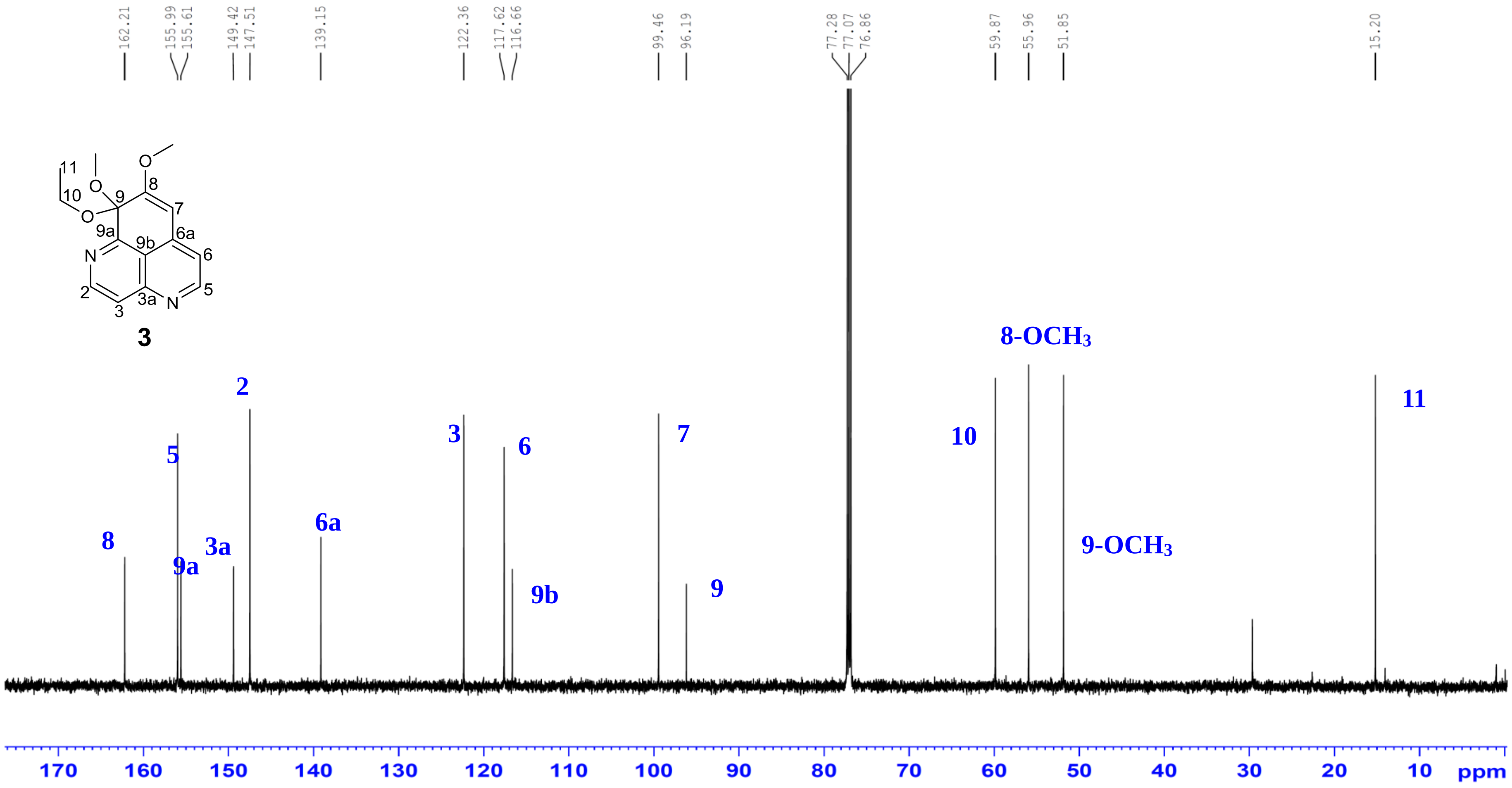

Figure S23. DEPT135 Spectrum of compound **3** in $CDCl_3$.

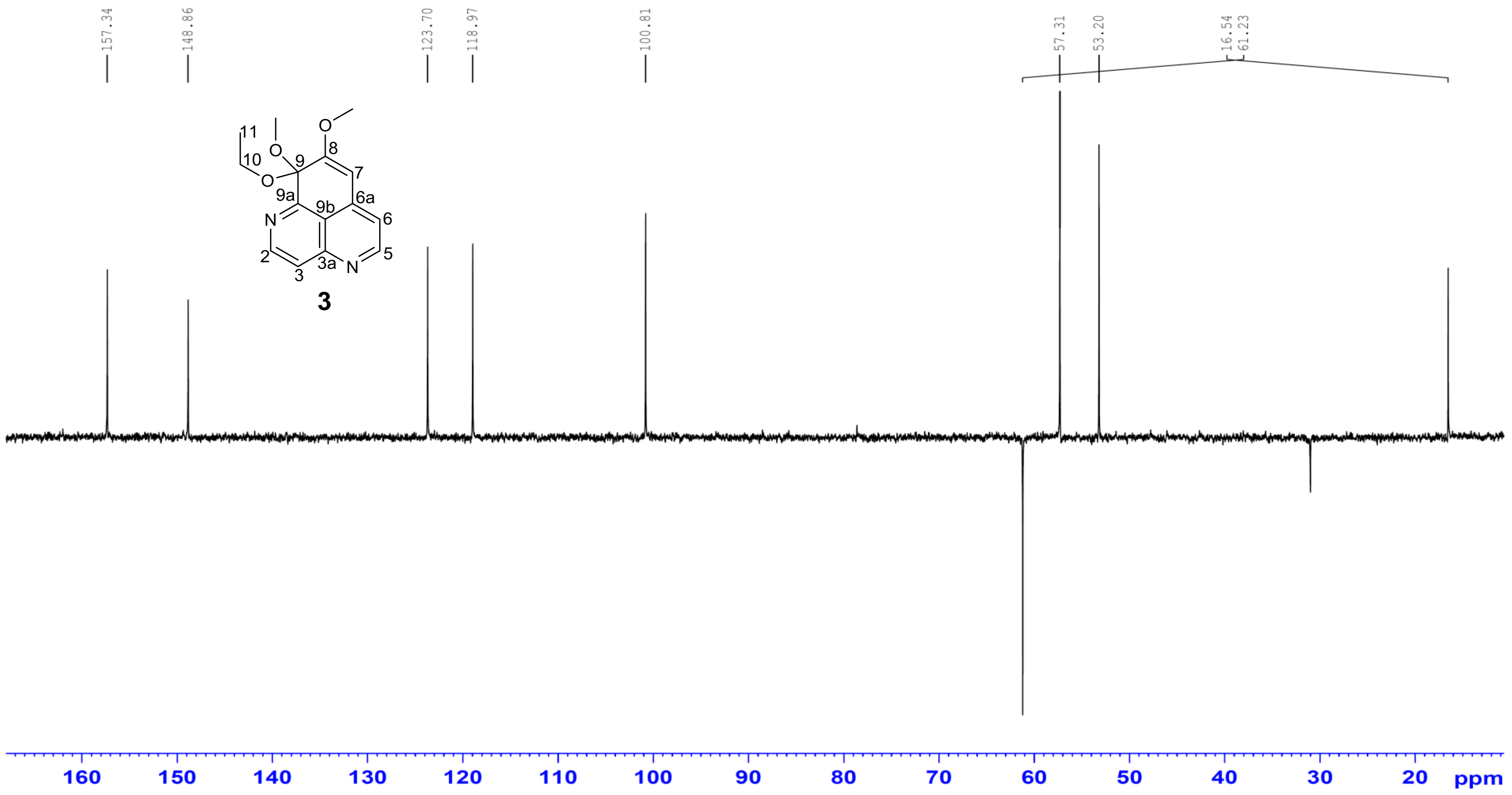

Figure S24. HSQC spectrum of compound **3** in CDCl3.

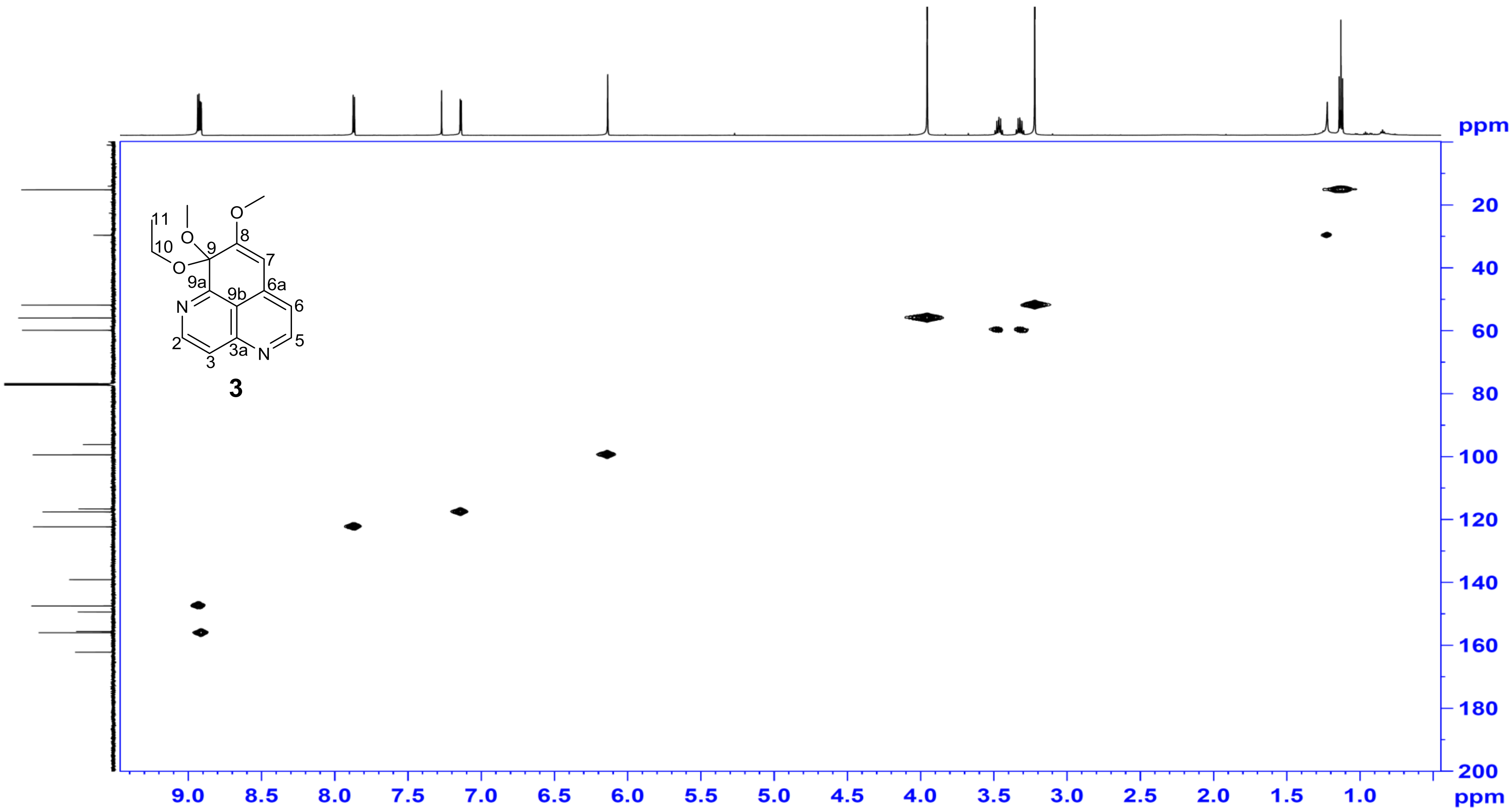

Figure S25. HMBC spectrum of compound **3** in $CDCl_3$.

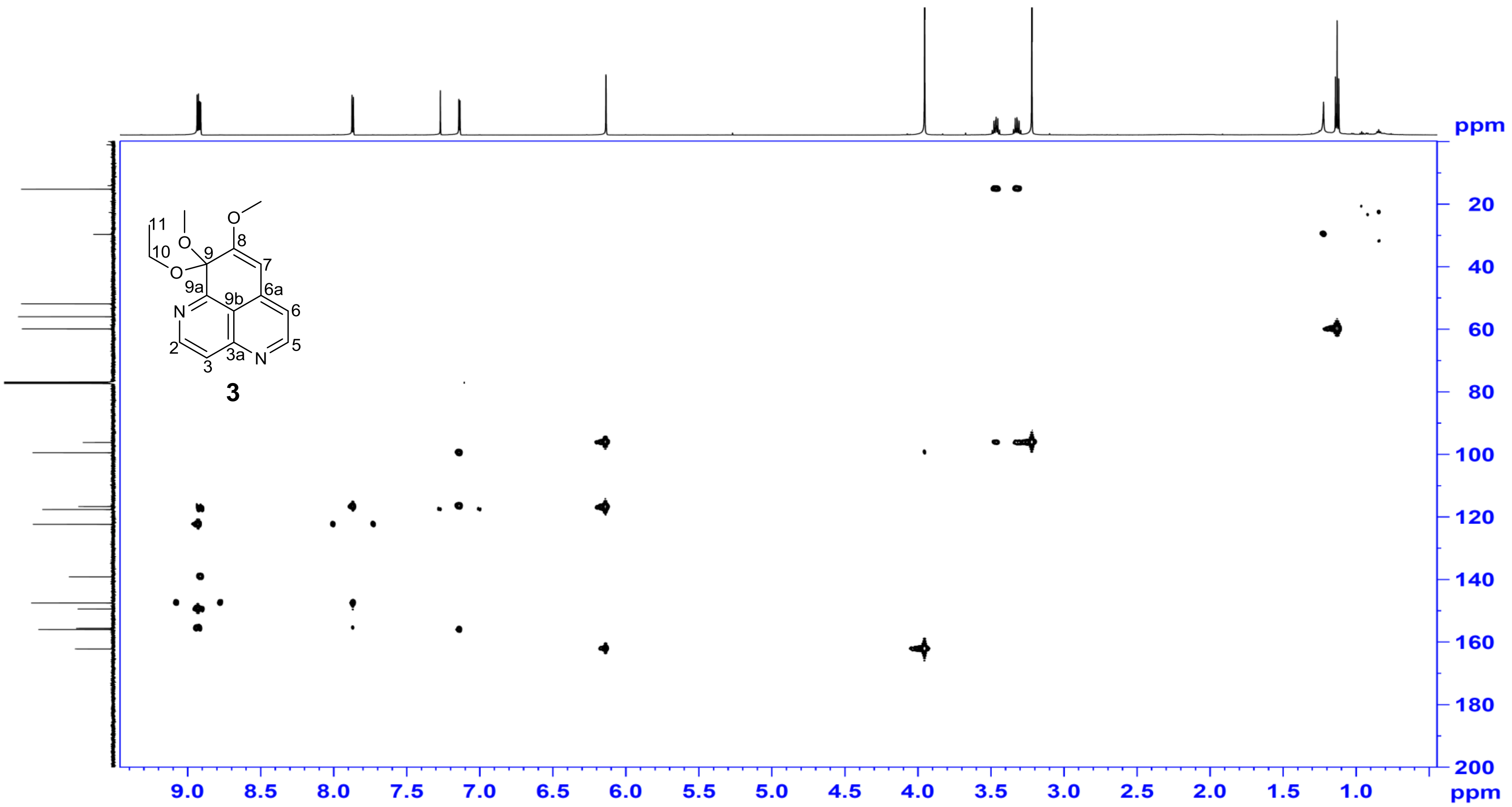

Figure S26. ^{1}H-^{1}H COSY spectrum of compound **3** in $CDCl_3$.

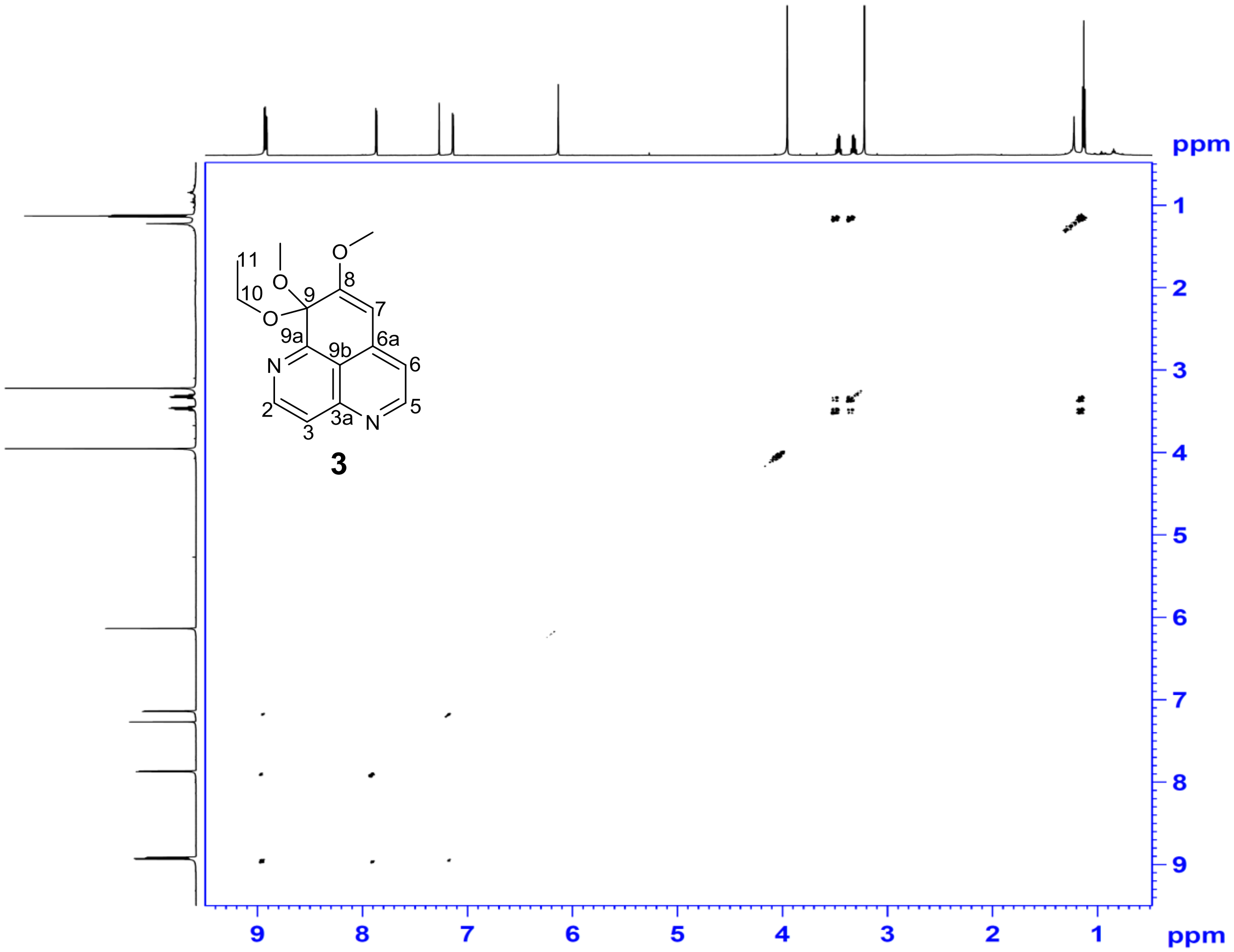

Figure S27. NOESY spectrum of compound **3** in $CDCl_3$.

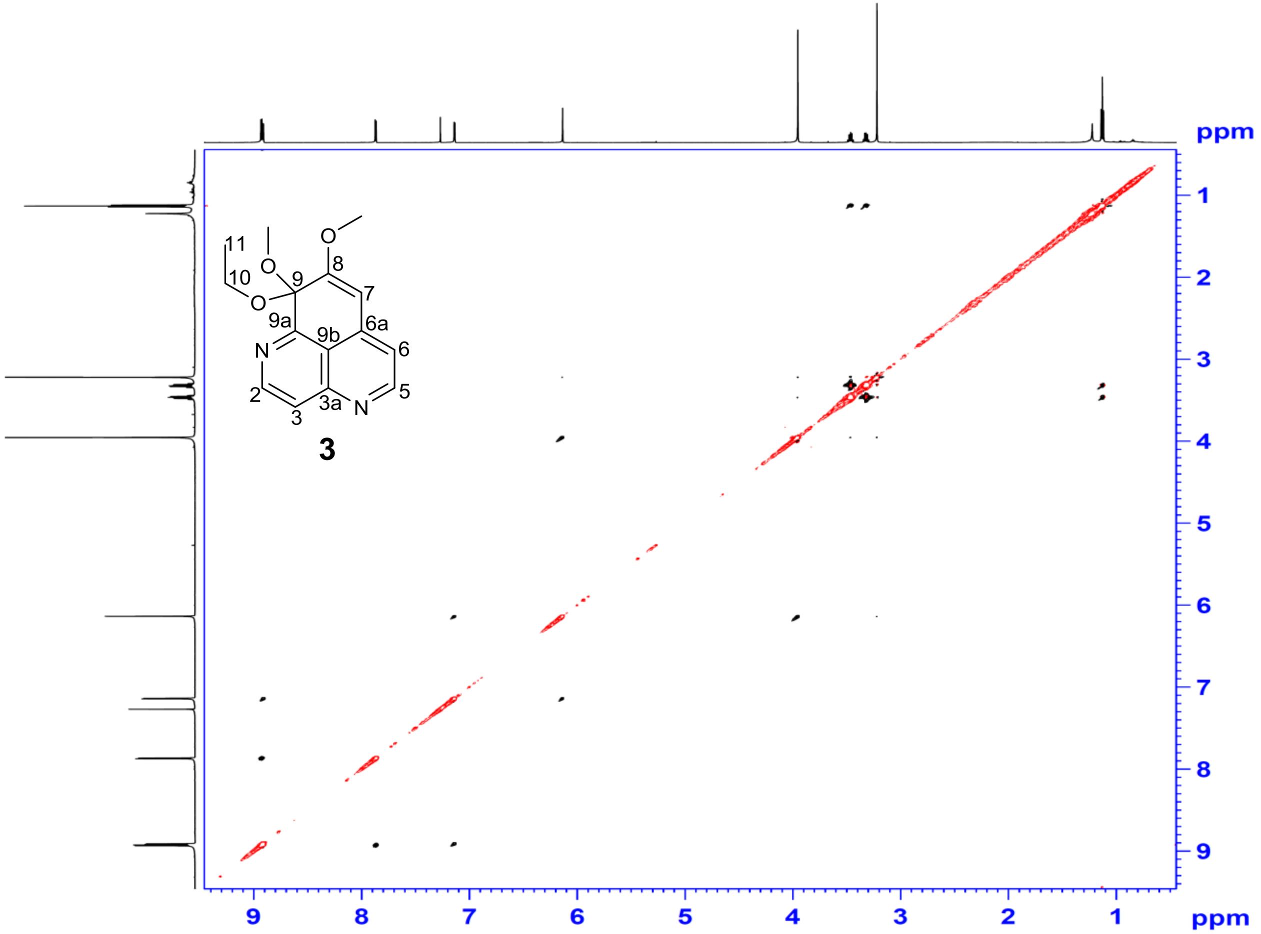

Figure S28. IR spectrum of compound **3**.

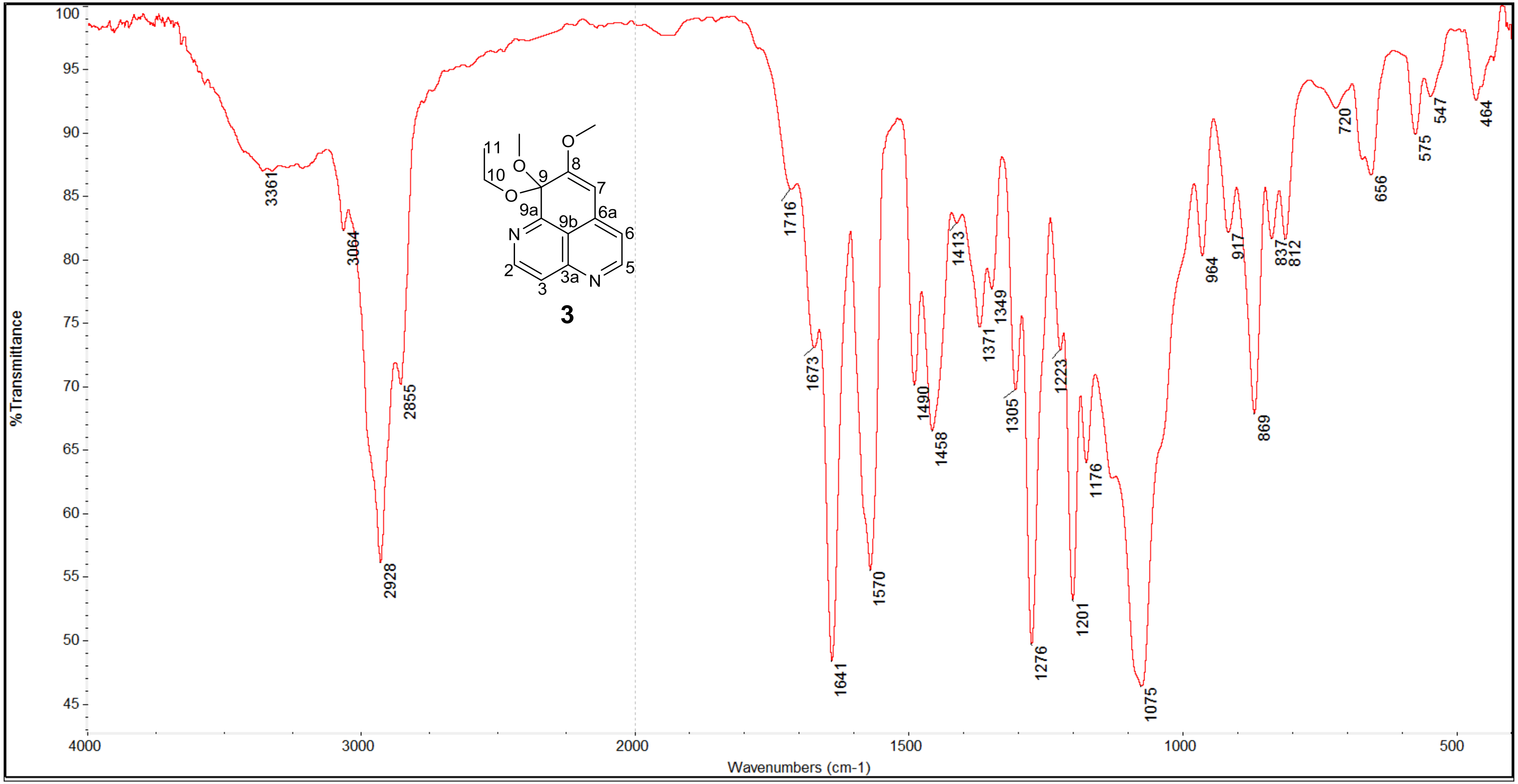

Figure S29. HRESIMS of compound **3**.

Elemental Composition Report **Page 1**

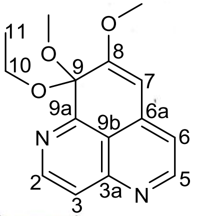

3

Multiple Mass Analysis: 2 mass(es) processed
Tolerance = 10.0 PPM / DBE: min = -1.5, max = 50.0
Selected filters: None

Monoisotopic Mass, Even Electron Ions
28 formula(e) evaluated with 1 results within limits (up to 50 closest results for each mass)
Elements Used:
C: 5-15 H: 10-25 N: 0-4 O: 0-5 Na: 1-1

SIPI
M.W=272
Q-Tof micro
YA019
22-Mar-2012,14:30:10
WQ12-149H 45 (1.553) AM (Cen,4, 80.00, Ar,5000.0,298.13,0.70); Sm (SG, 2x1.00); Cm (33:45)
TOF MS ES+
1.23e3

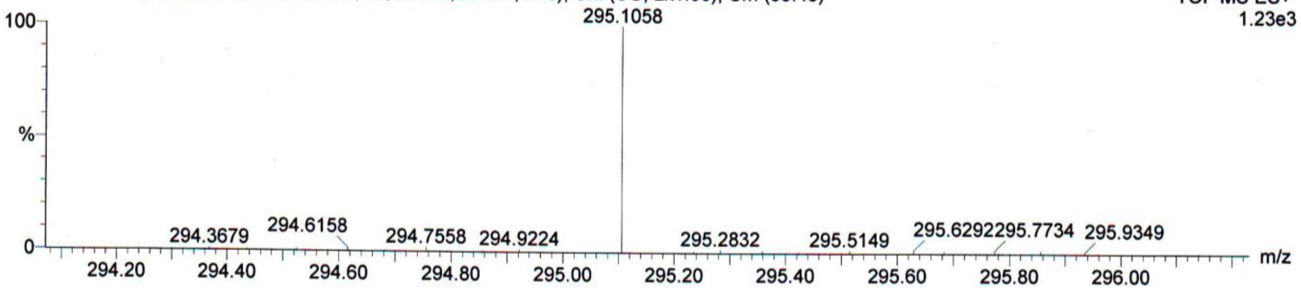

Minimum:	85.00				-1.5		
Maximum:	100.00		5.0	10.0	50.0		
Mass	RA	Calc. Mass	mDa	PPM	DBE	i-FIT	Formula
295.1058	100.00	295.1059	-0.1	-0.3	8.5	5546620.0	C15 H16 N2 O3 Na

Figure S30. UV spectrum of compound **3**.

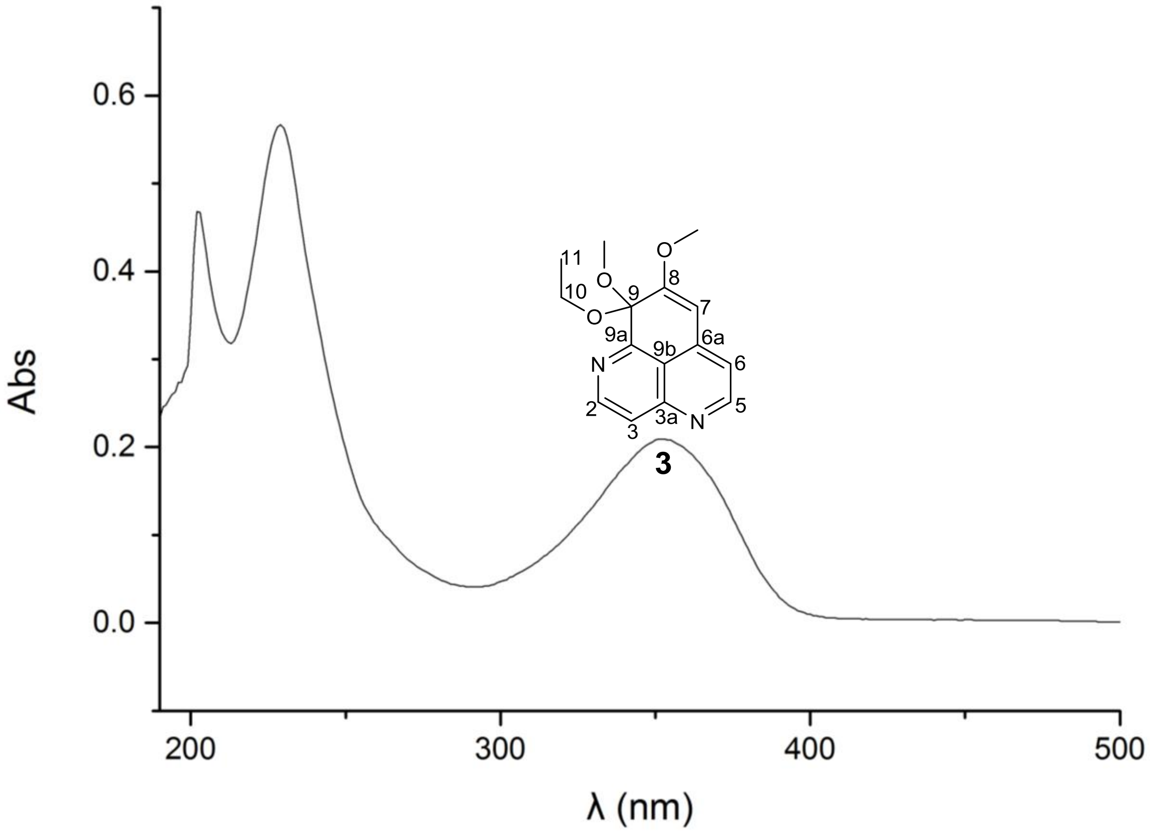

Figure S31. ^{1}H NMR spectrum of compound **4** in $CDCl_3$.

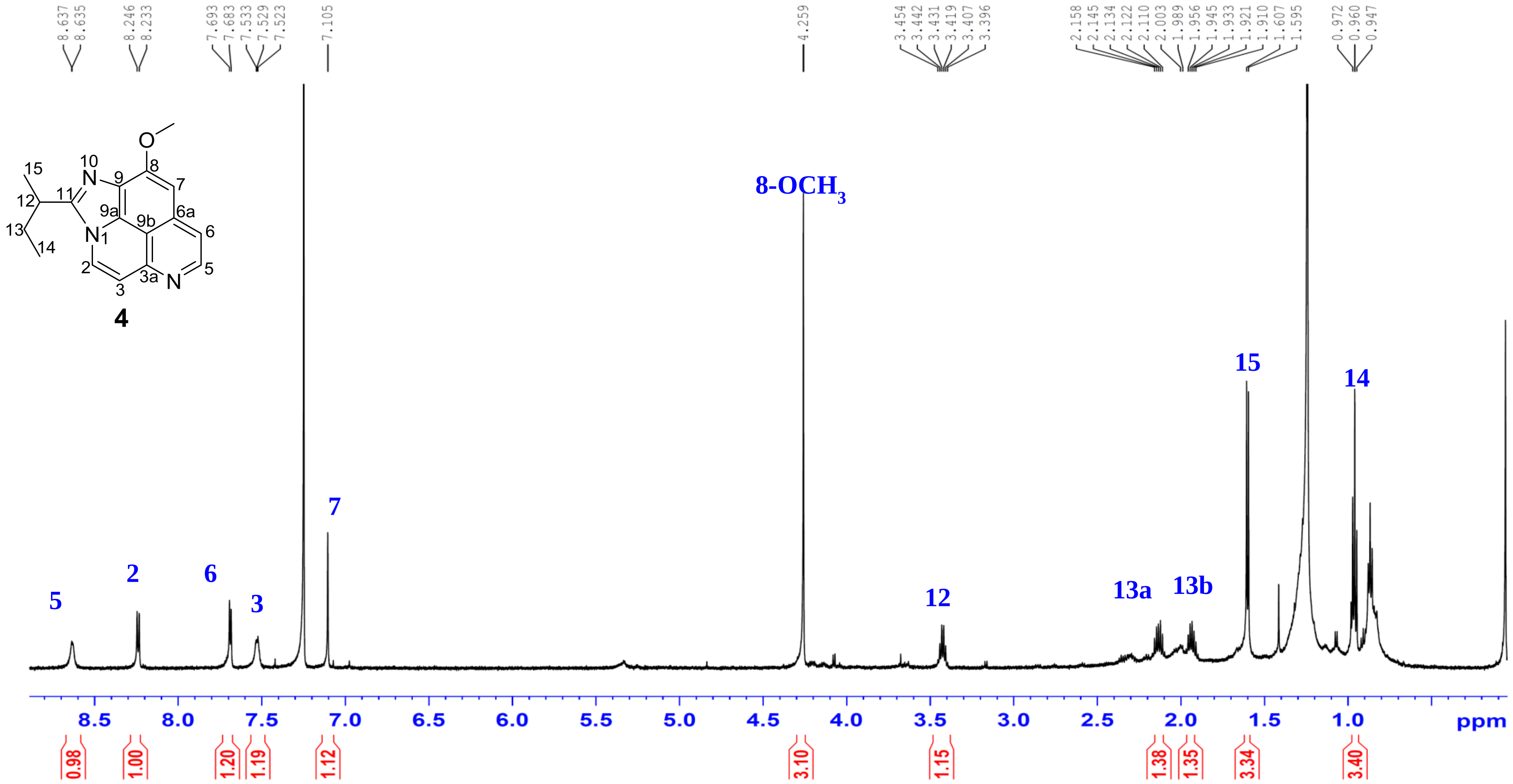

Figure S32. ^{13}C NMR spectrum of compound **4** in $CDCl_3$.

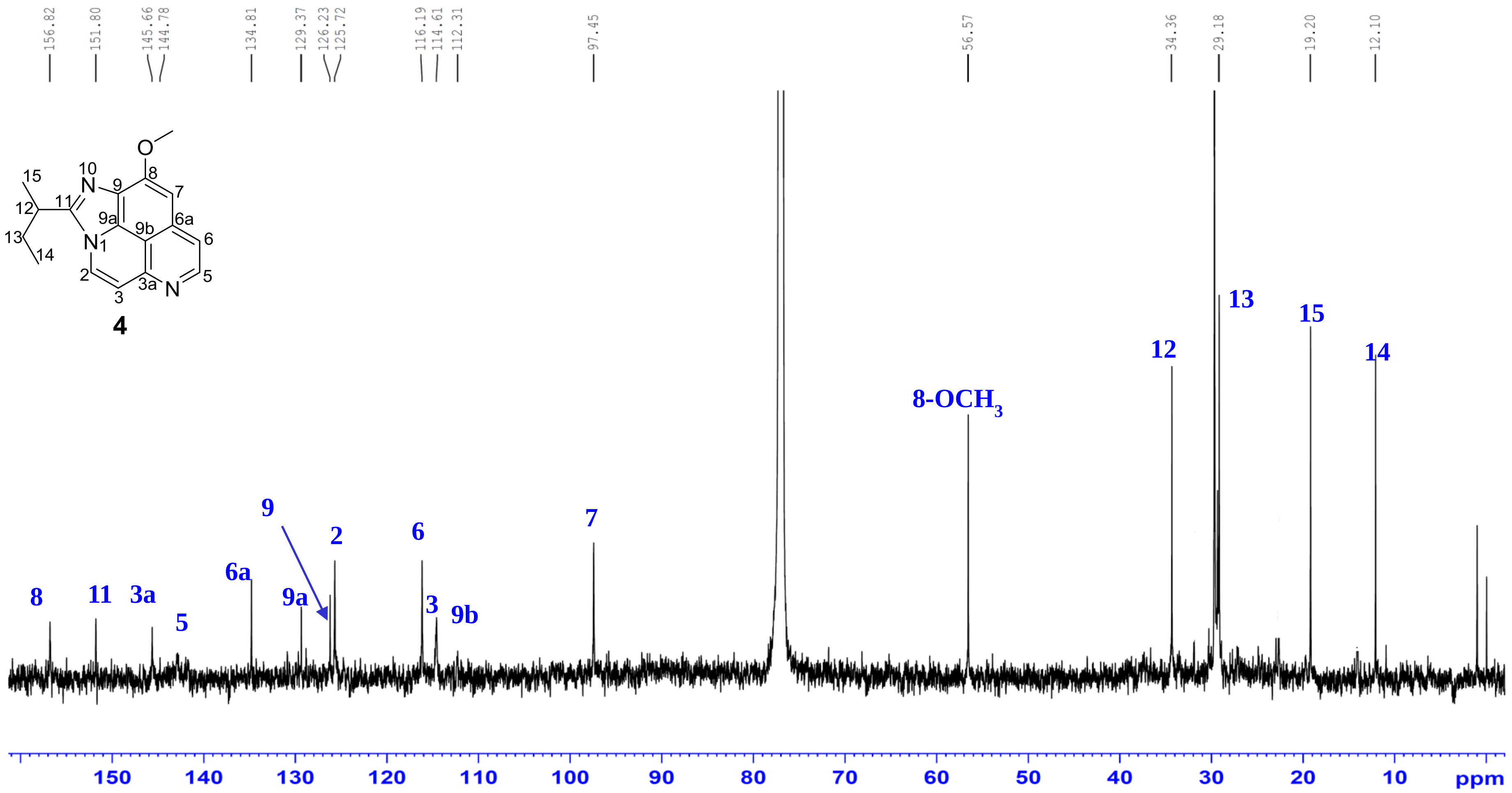

Figure S33. DEPT135 Spectrum of compound **4** in $CDCl_3$.

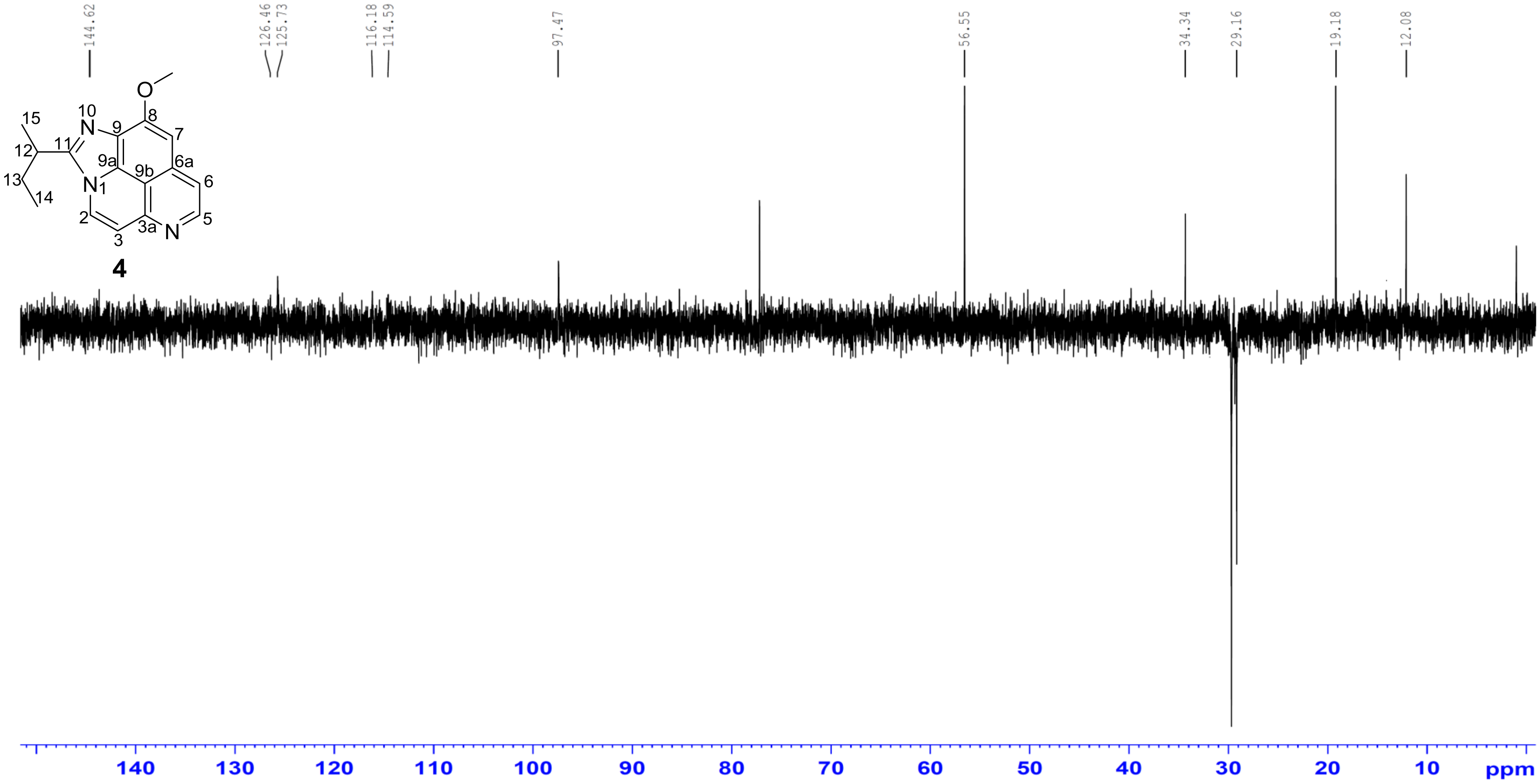

Figure S34. HSQC spectrum of compound **4** in $CDCl_3$.

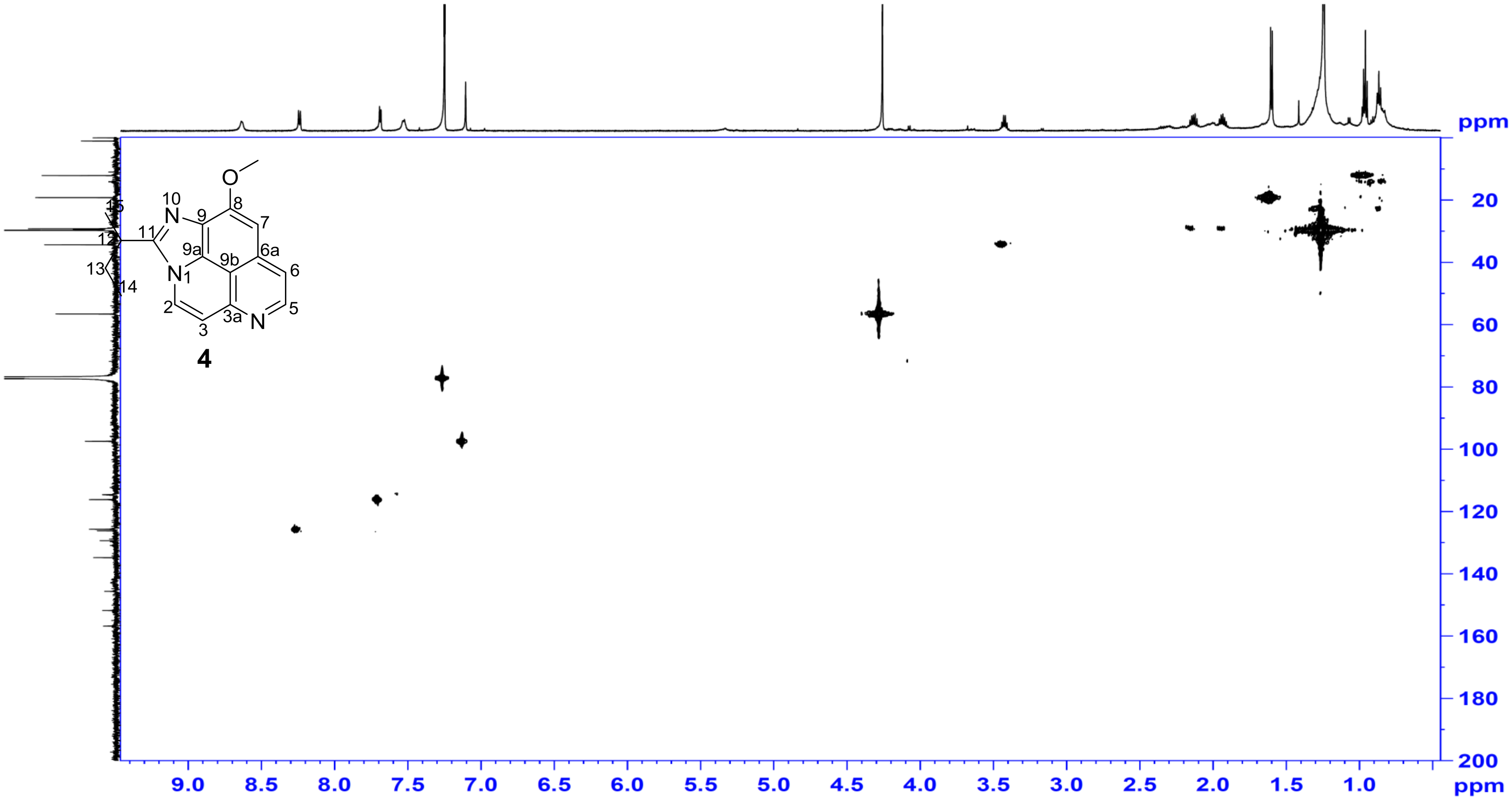

Figure S35. HMBC spectrum of compound **4** in CDCl3.

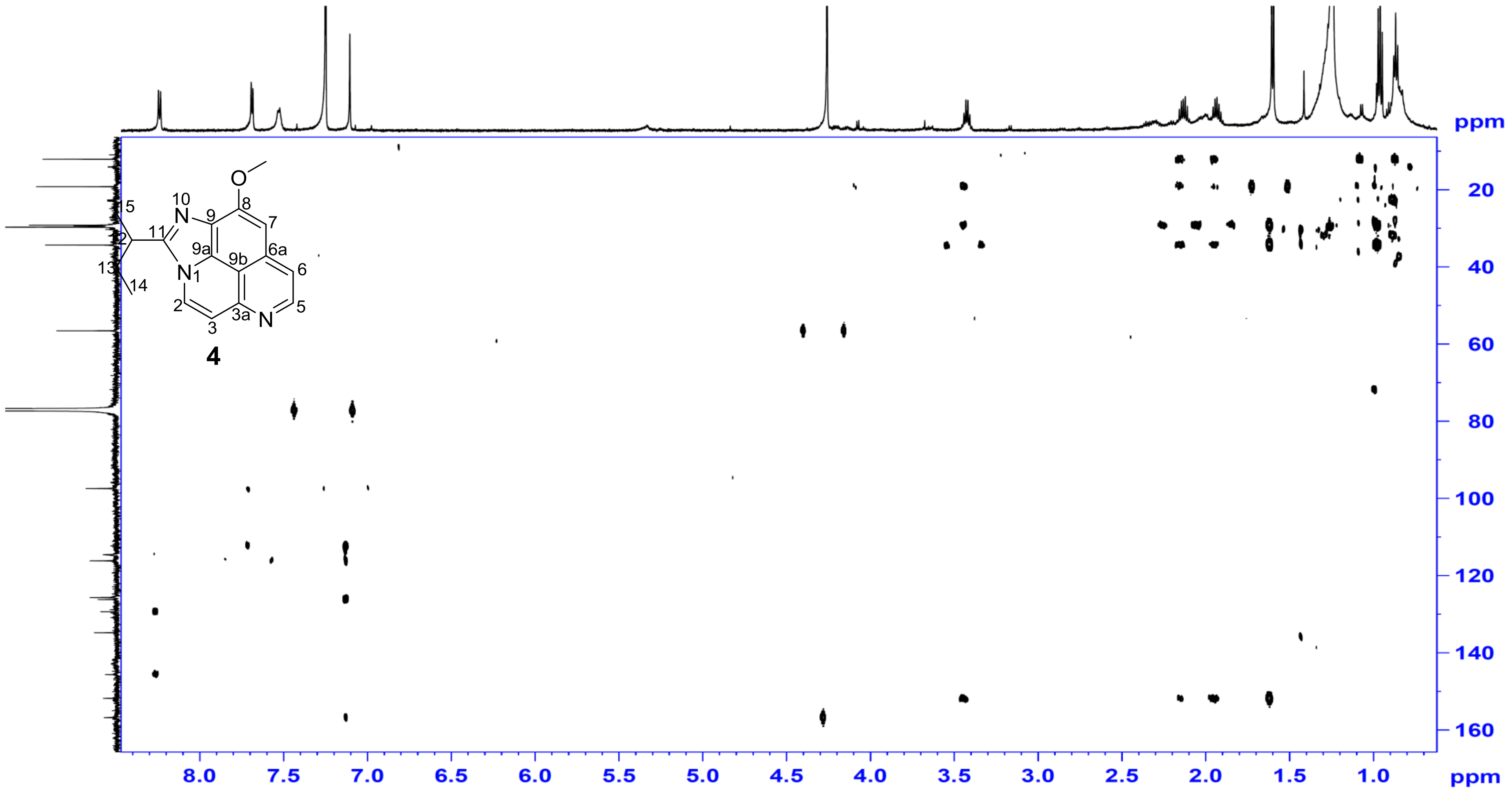

Figure S36. ^{1}H-^{1}H COSY spectrum of compound **4** in $CDCl_3$.

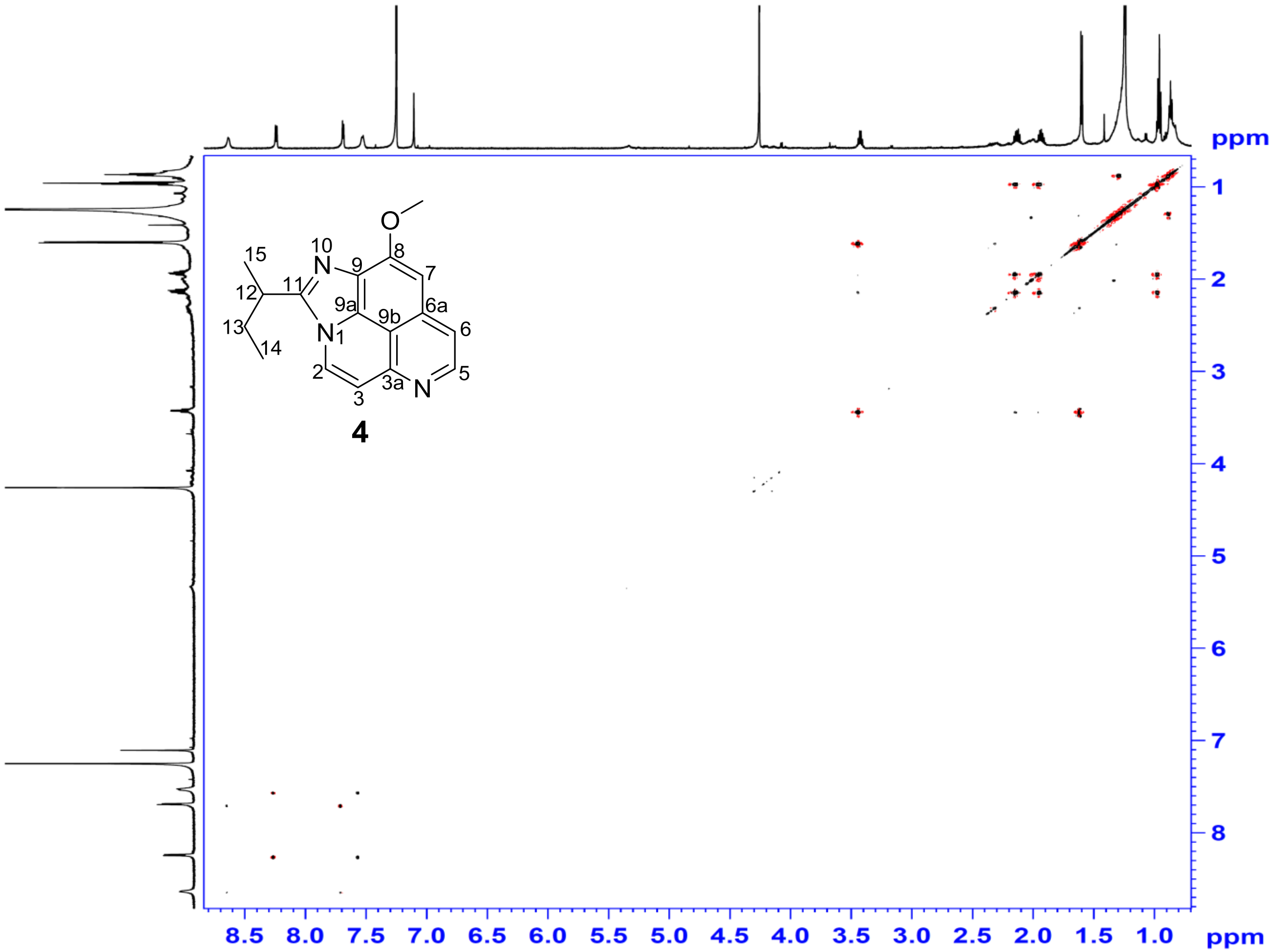

Figure S37. NOESY spectrum of compound **4** in $CDCl_3$.

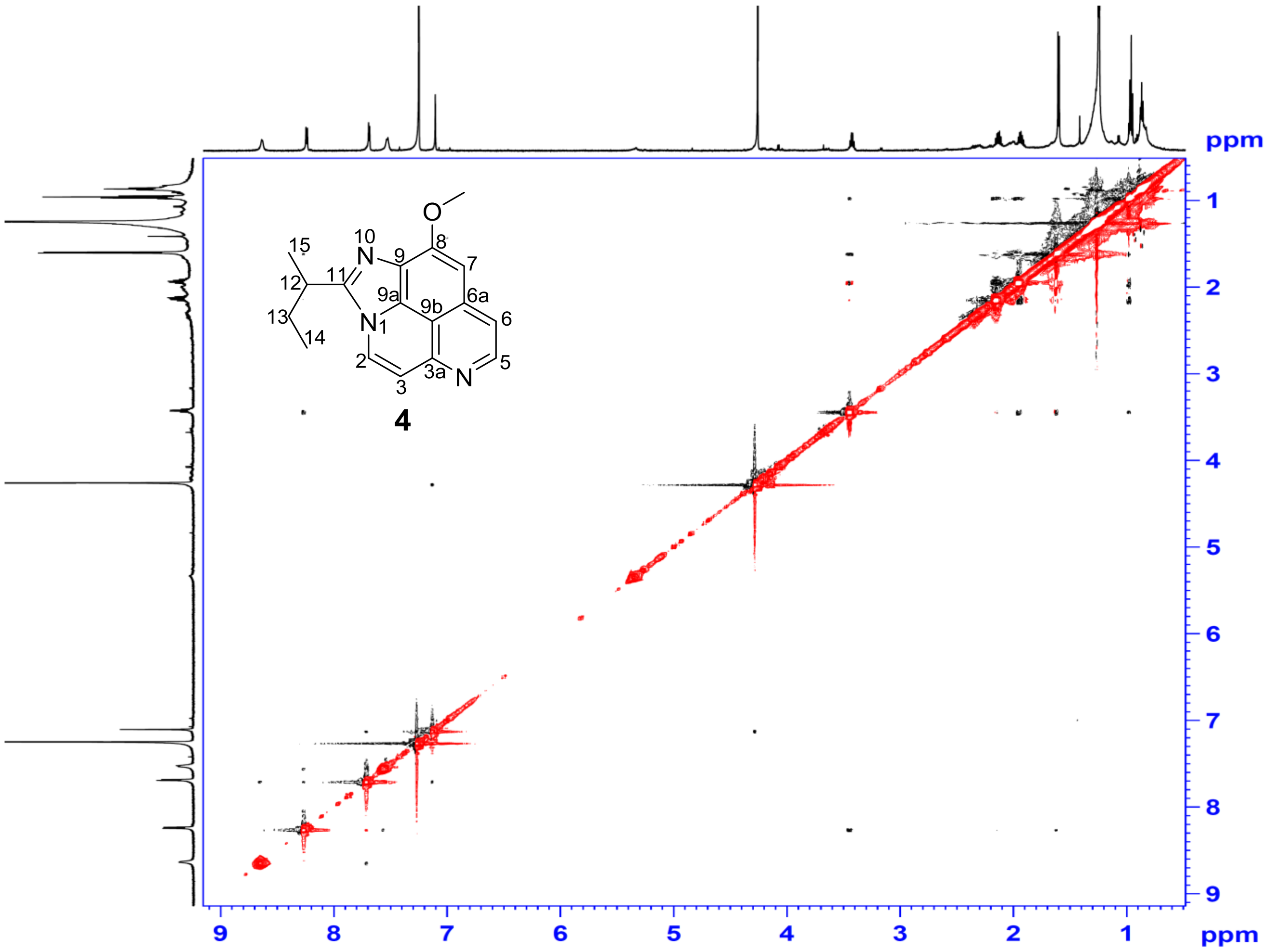

Figure S38. IR spectrum of compound **4**.

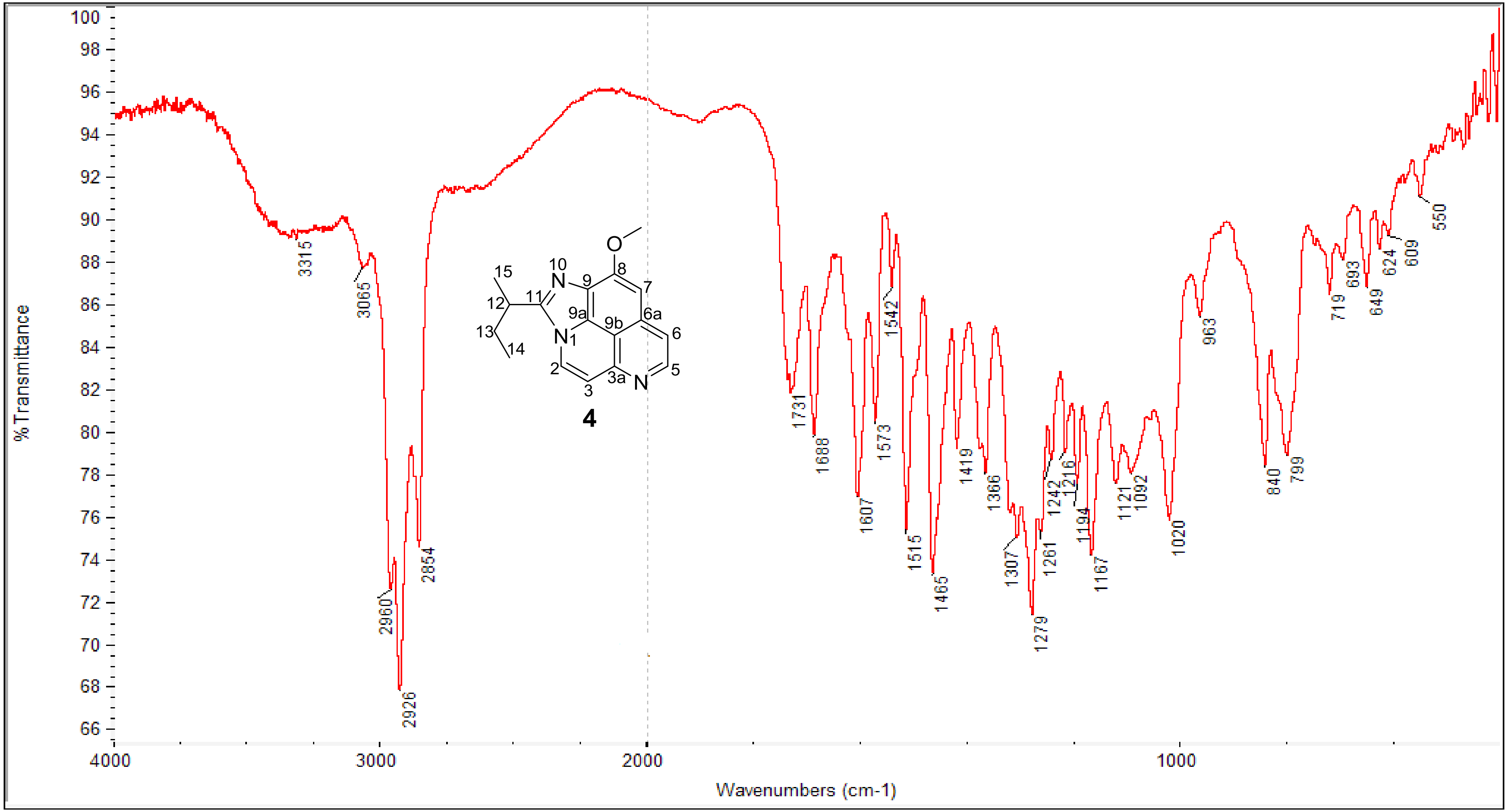

Figure S39. HRESIMS of compound **4**.

Elemental Composition Report

Page 1

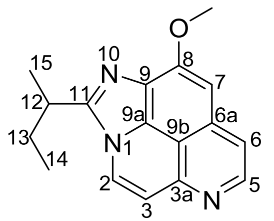

4

Tolerance = 5.0 PPM / DBE: min = -1.5, max = 50.0
Selected filters: None

Monoisotopic Mass, Even Electron Ions
12 formula(e) evaluated with 1 results within limits (up to 50 closest results for each mass)
Elements Used:
C: 5-20 H: 5-20 N: 1-3 O: 1-3

SIPI
M.W=279
Q-Tof micro
YA019
31-Oct-2013,15:52:17
WQ13-259H1 55 (1.894) AM (Cen,4, 80.00, Ar,5000.0,268.14,0.70); Sm (Mn, 2x3.00); Cm (49:66)
TOF MS ES+
2.42e3

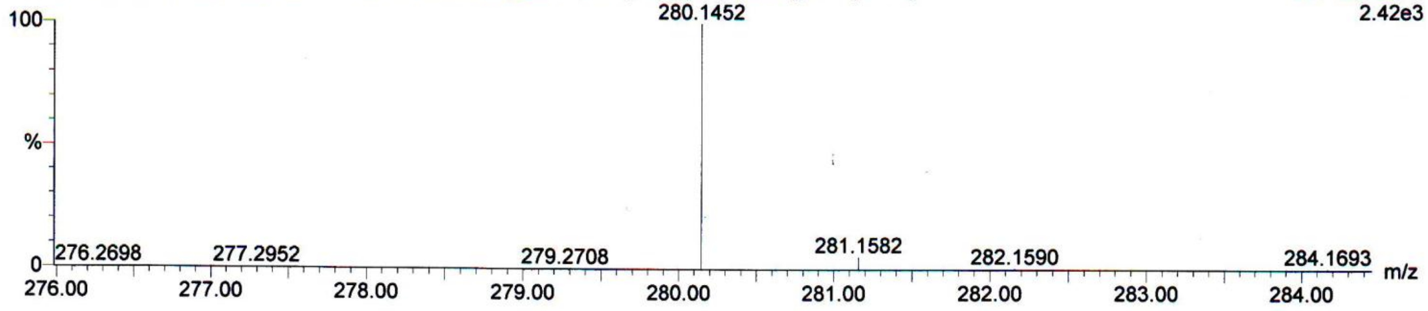

Minimum:	75.00				-1.5		
Maximum:	100.00		5.0	5.0	50.0		
Mass	RA	Calc. Mass	mDa	PPM	DBE	i-FIT	Formula
280.1452	100.00	280.1450	0.2	0.7	10.5	366.1	C17 H18 N3 O

Figure S40. UV spectrum of compound **4**.

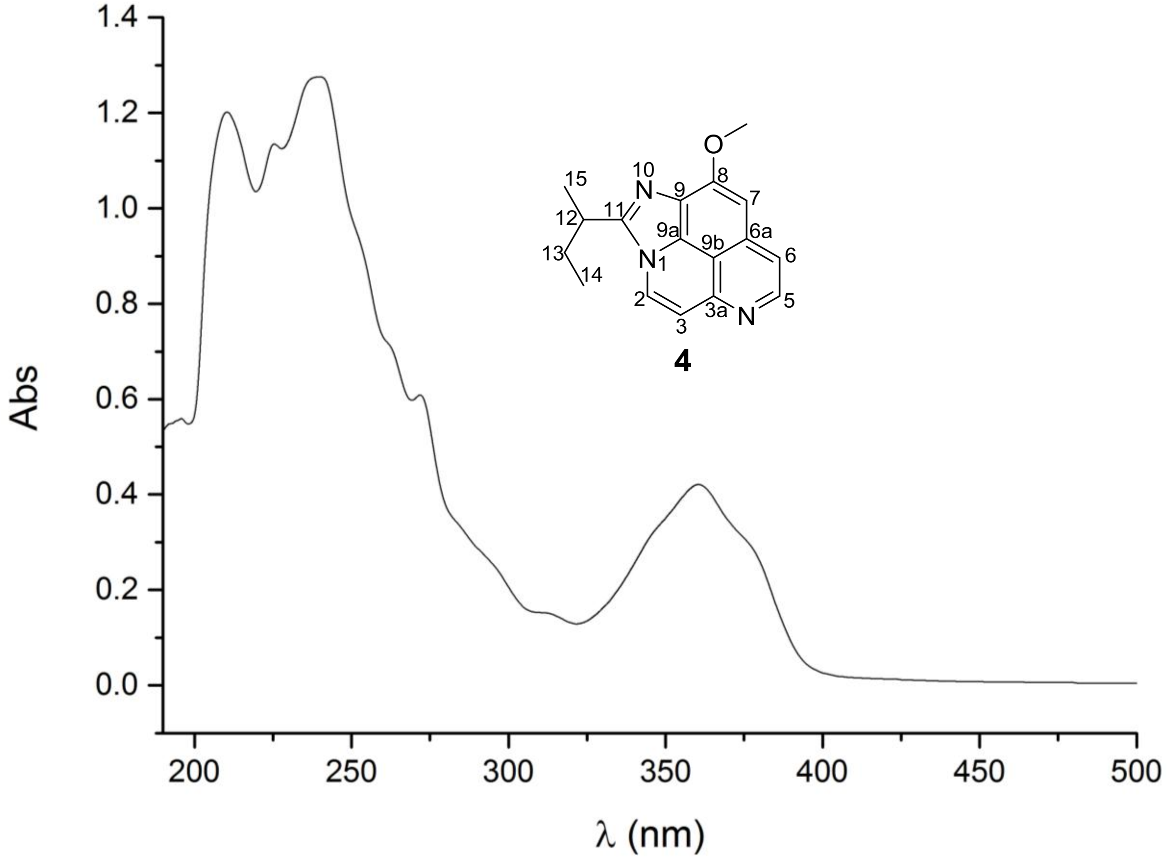

Figure S41. ^{1}H NMR spectrum of compound **5** in $CDCl_3$.

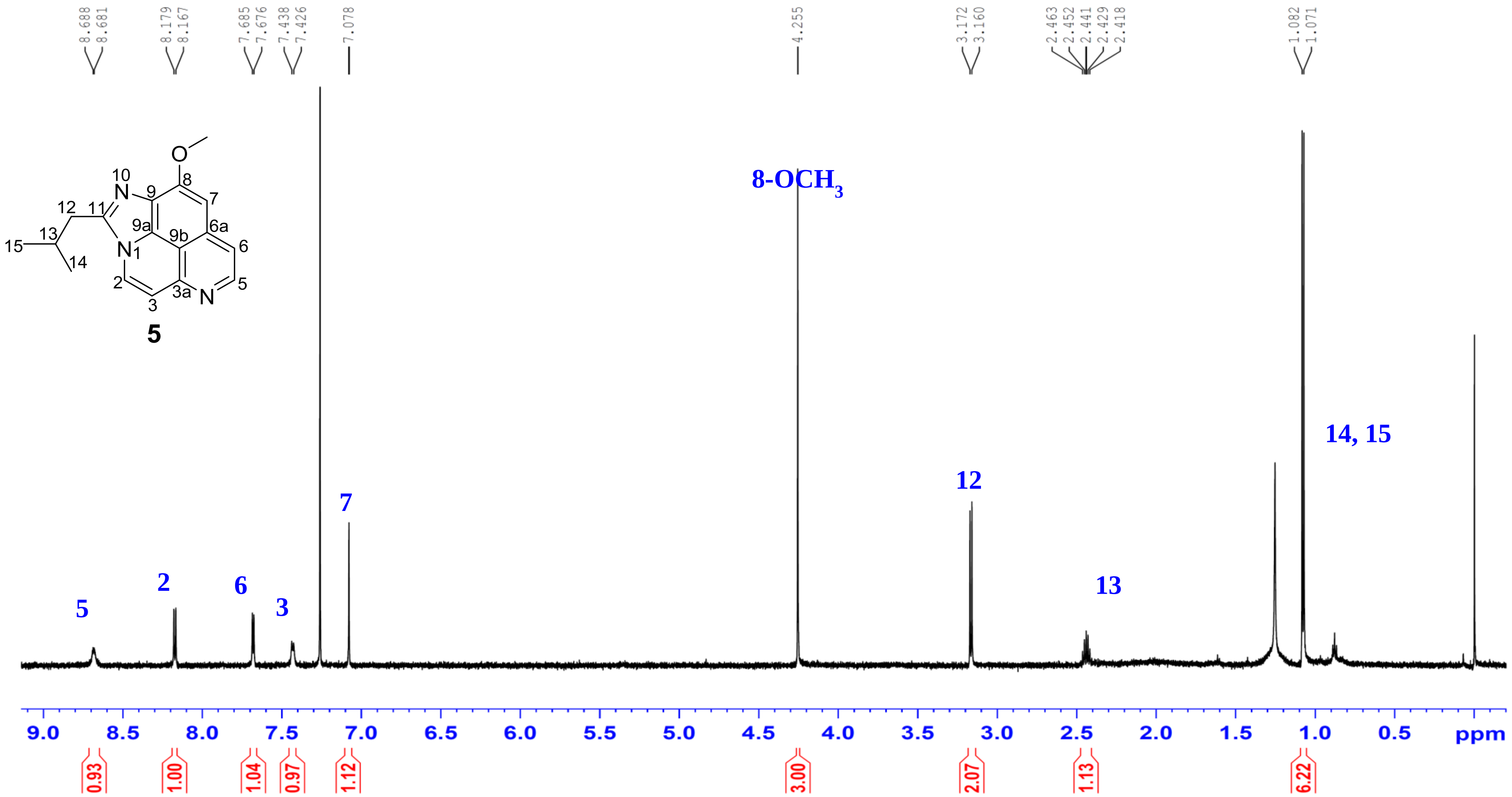

Figure S42. ^{13}C NMR spectrum of compound **5** in $CDCl_3$.

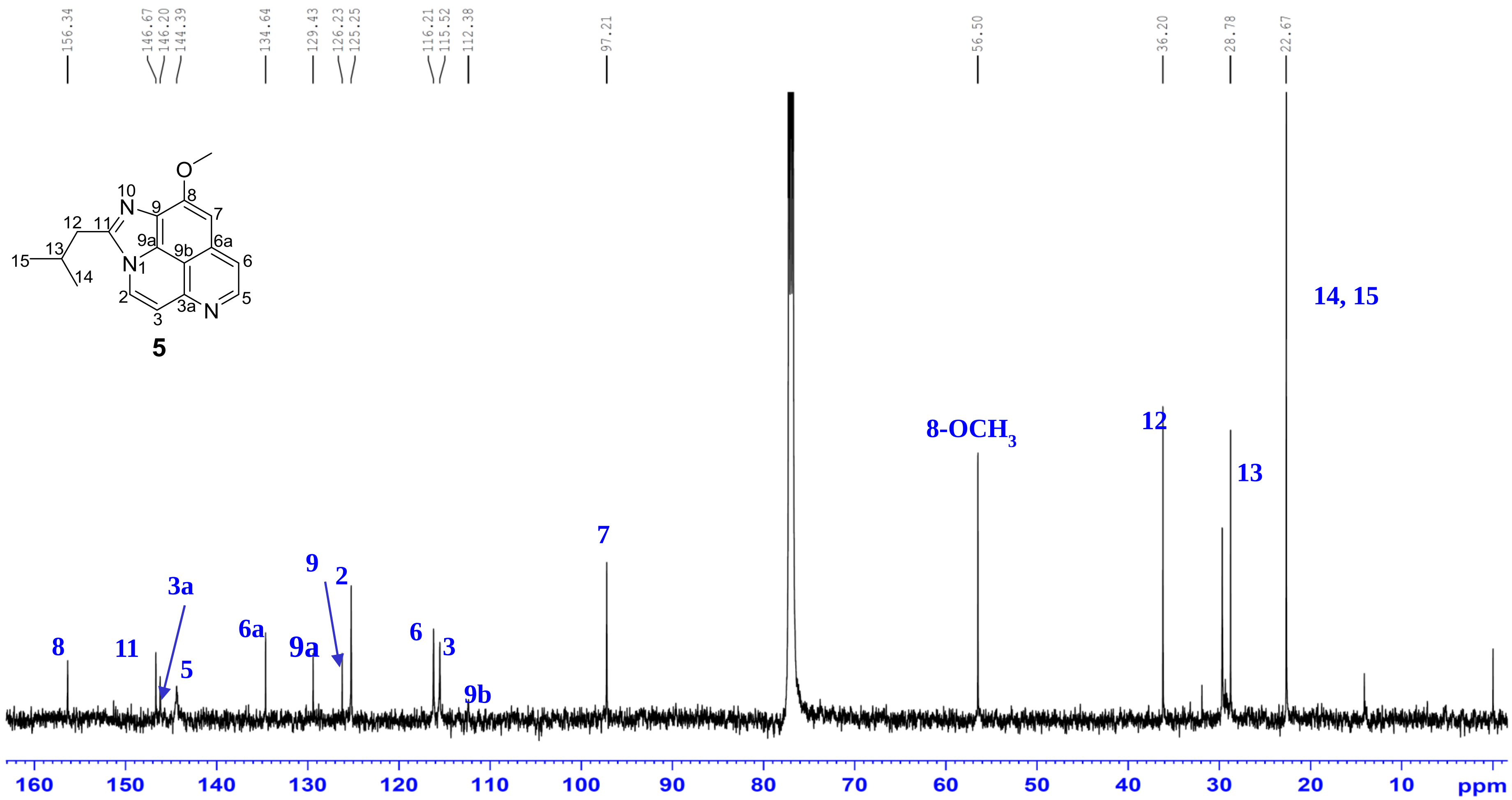

Figure S43. DEPT135 Spectrum of compound **5** in $CDCl_3$.

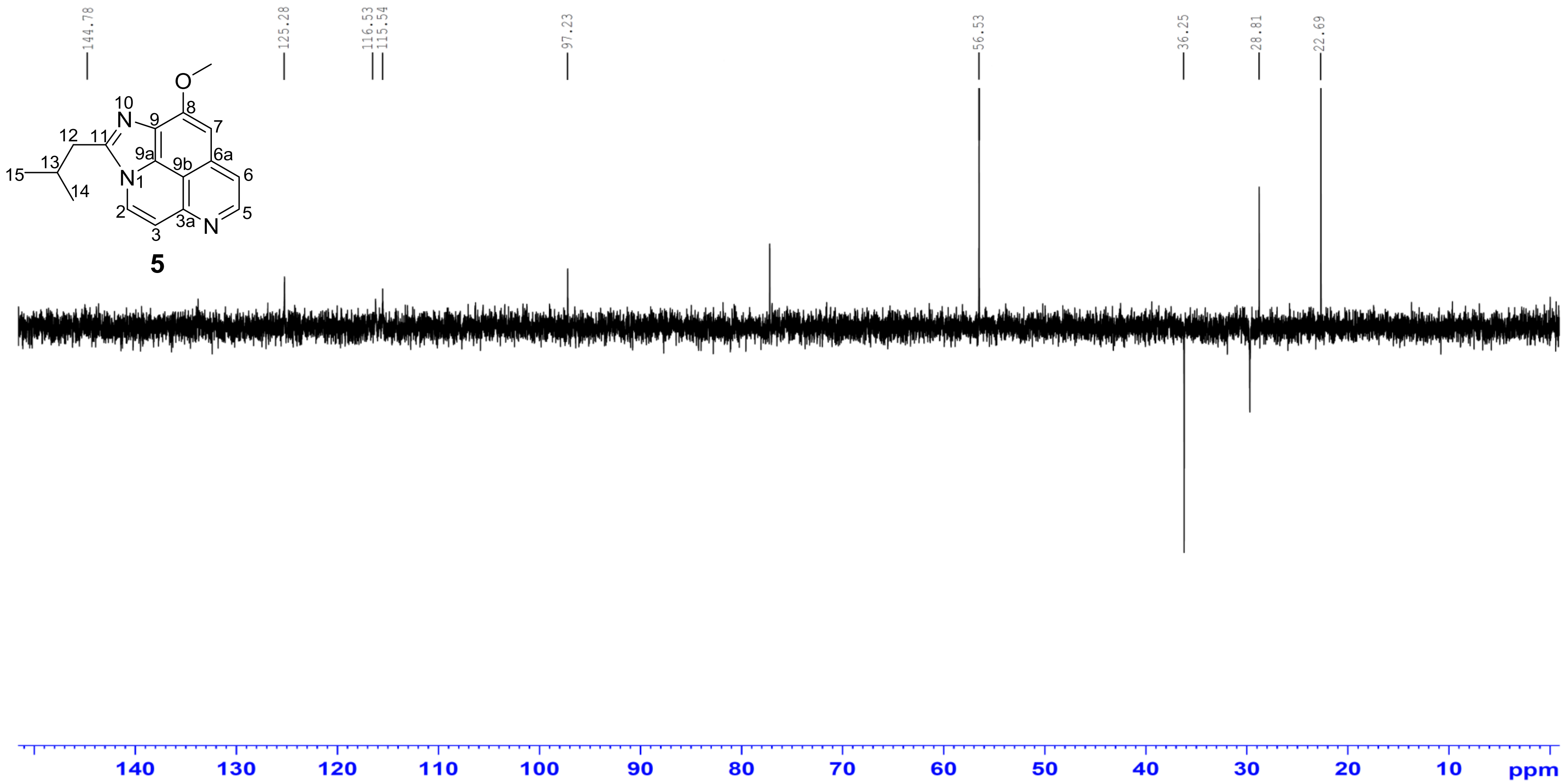

Figure S44. HSQC spectrum of compound **5** in $CDCl_3$.

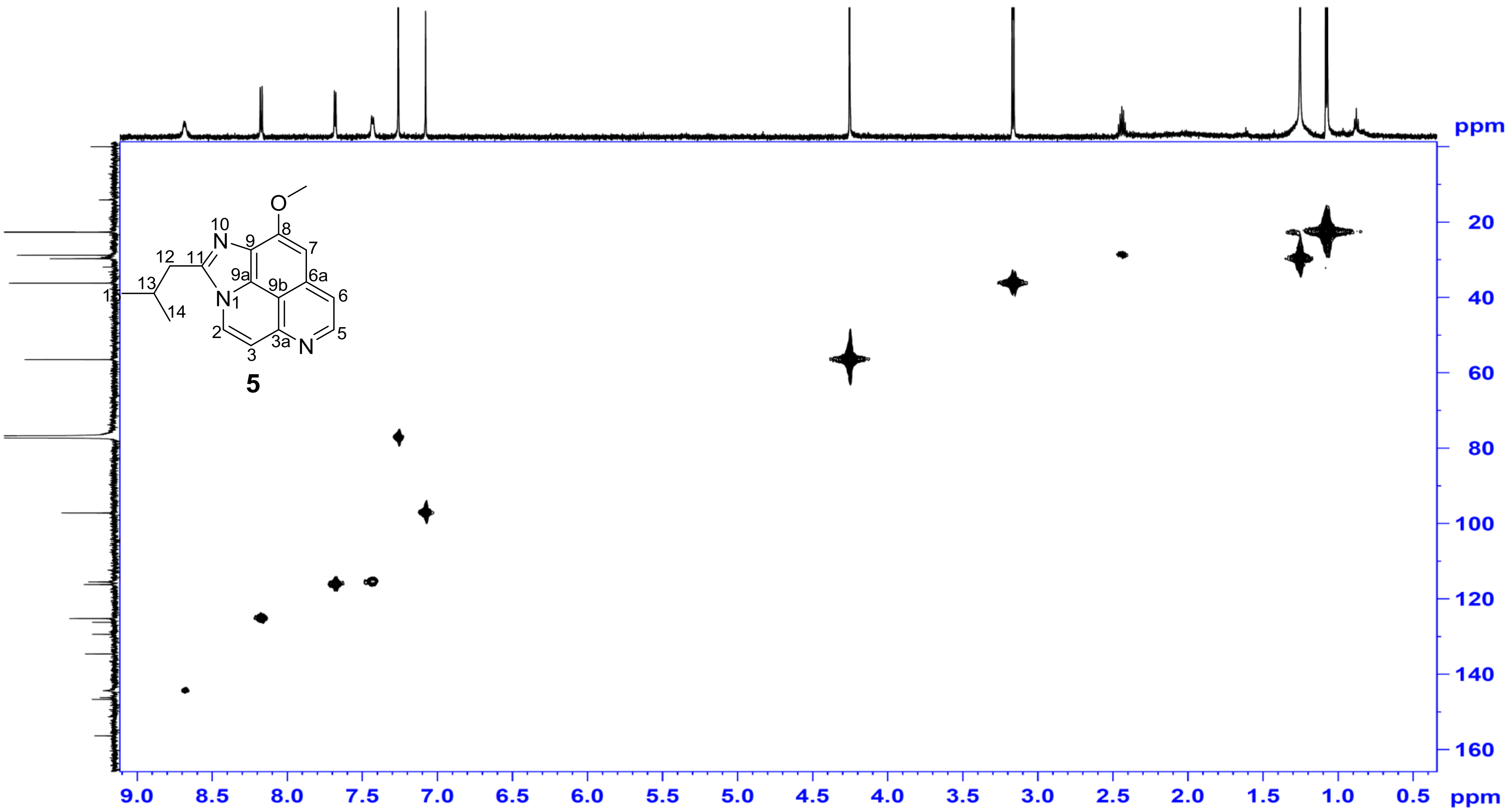

Figure S45. HMBC spectrum of compound **5** in $CDCl_3$.

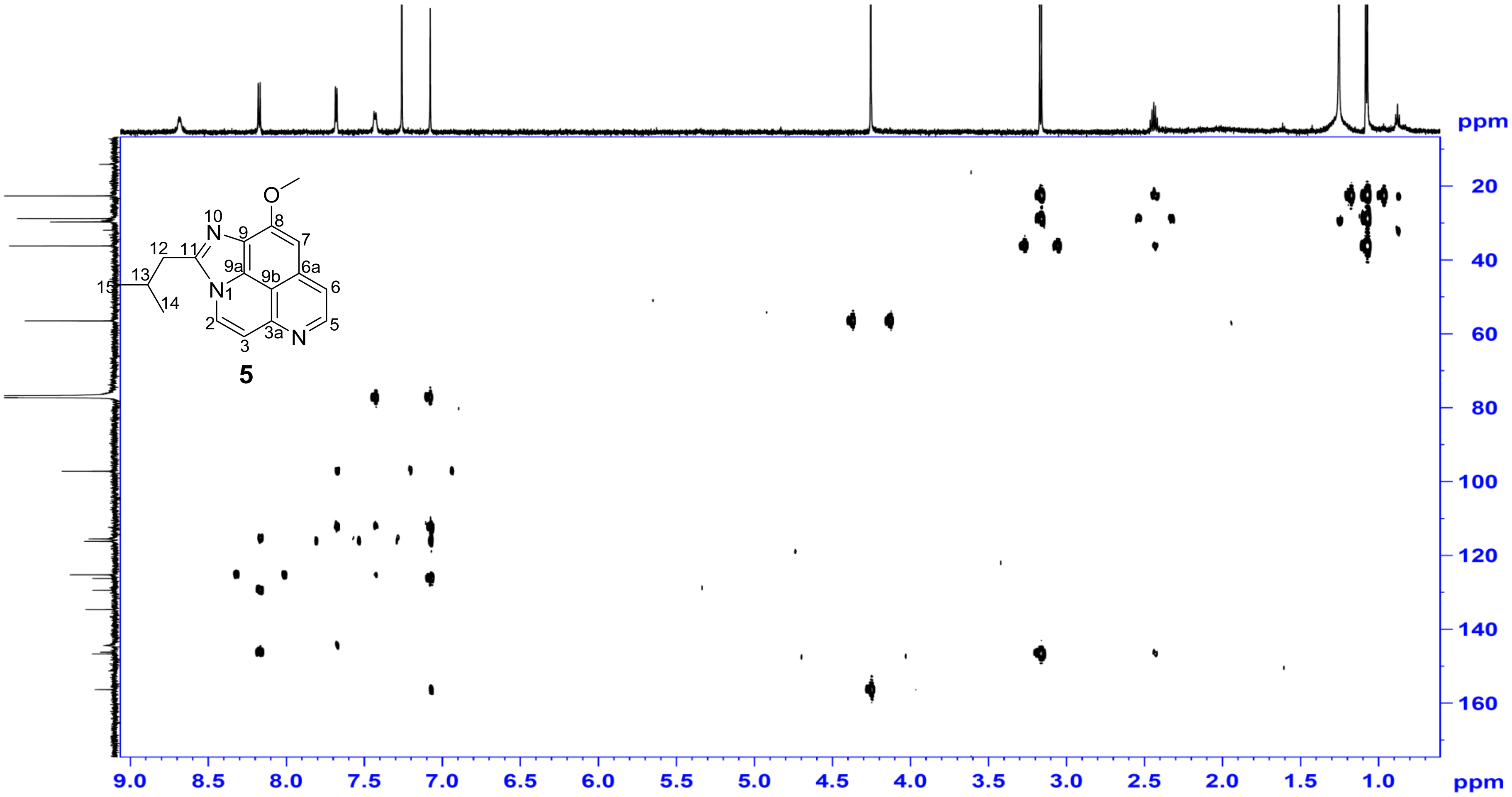

Figure S46. ^{1}H-^{1}H COSY spectrum of compound **5** in $CDCl_3$.

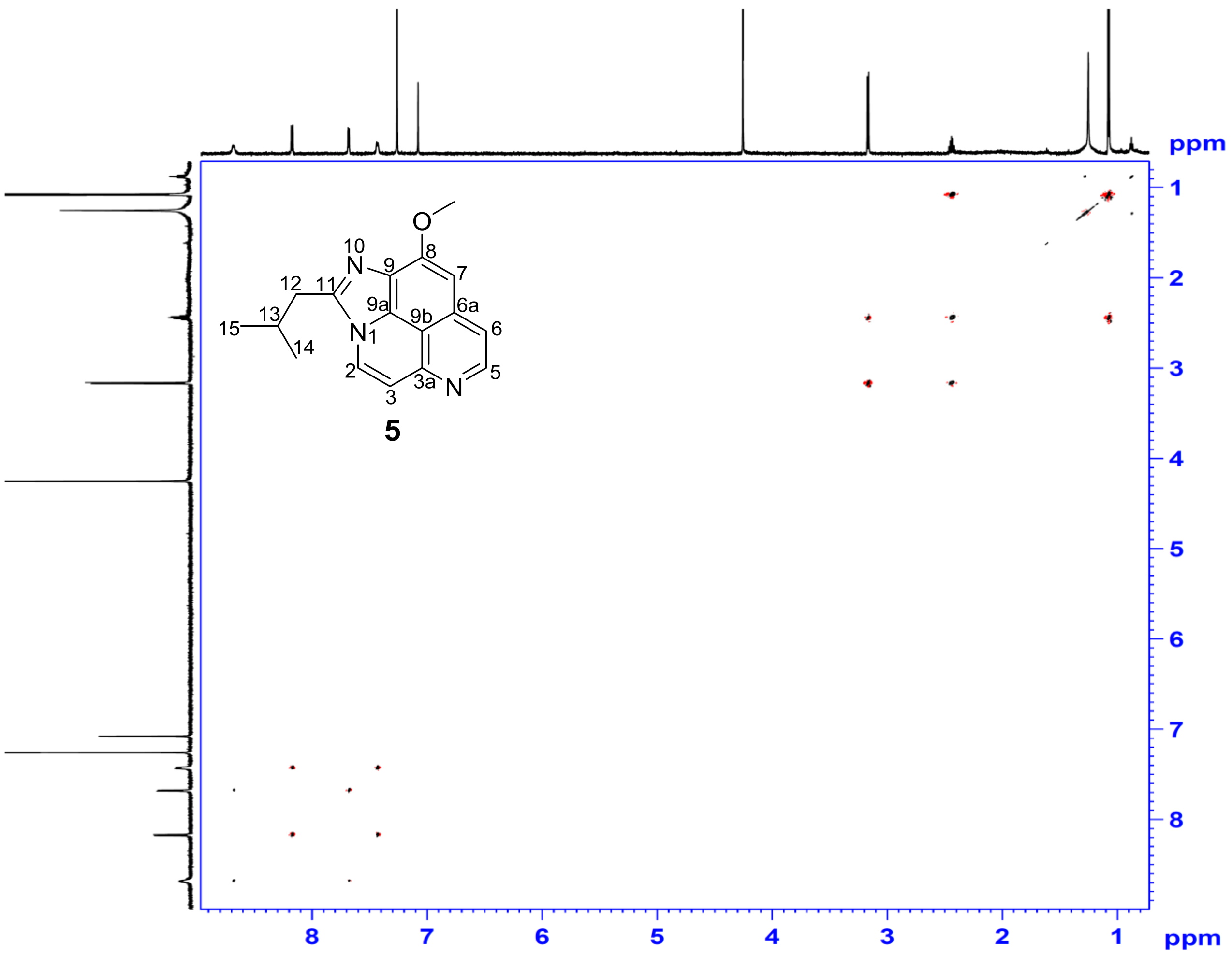

Figure S47. NOESY spectrum of compound **5** in $CDCl_3$.

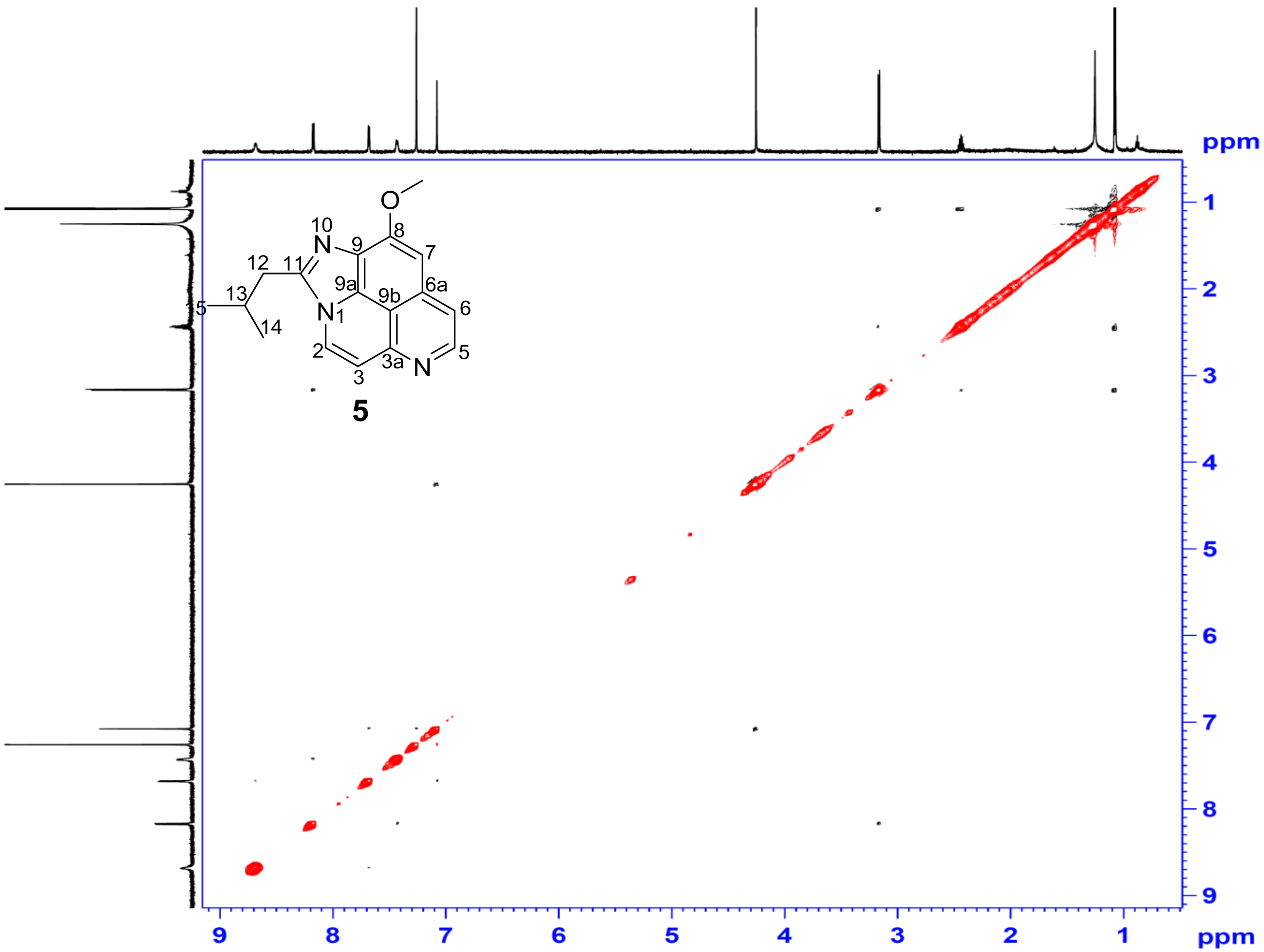

Figure S48. IR spectrum of compound **5**.

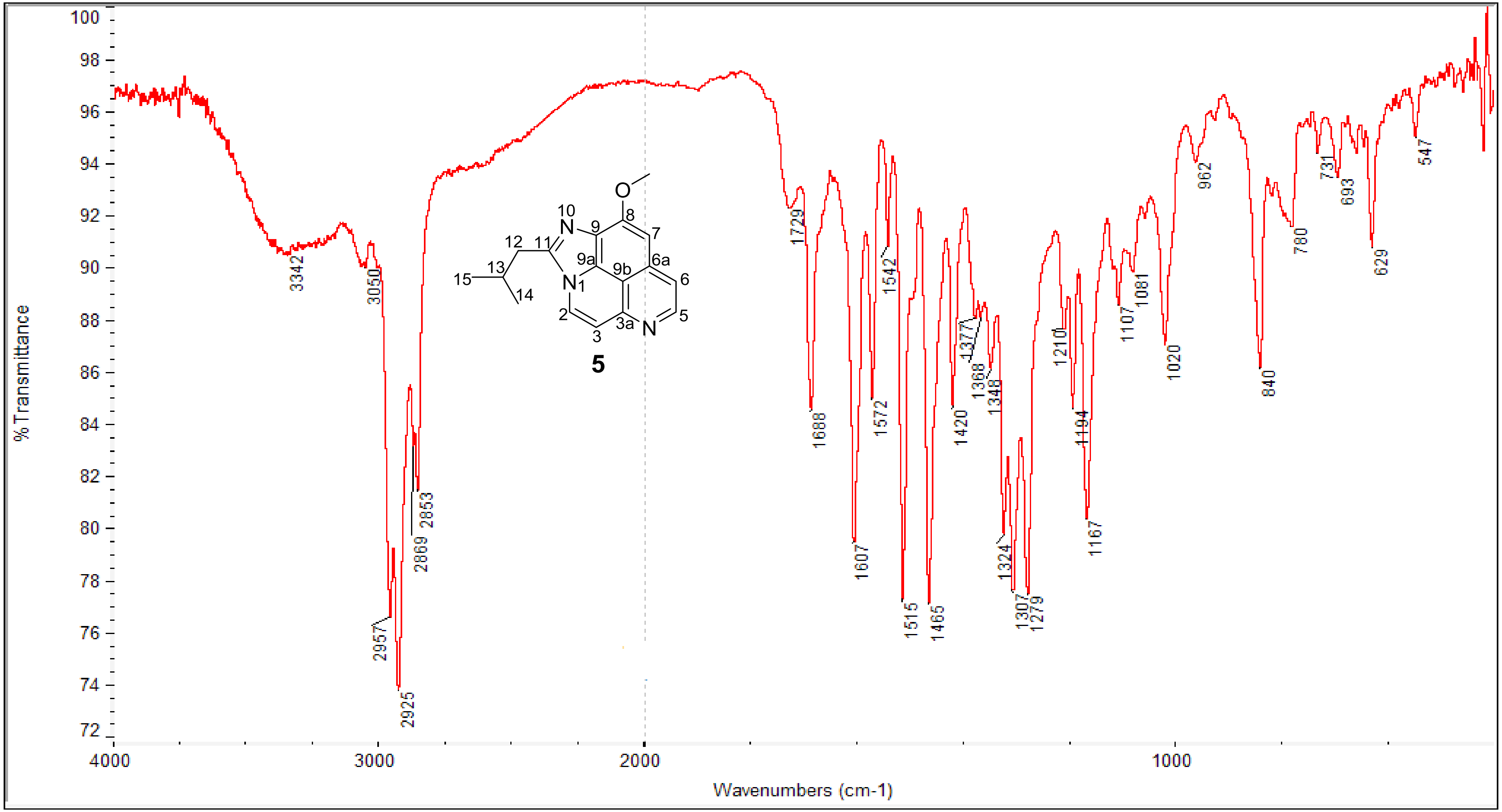

Figure S49. HRESIMS of compound **5**.

Elemental Composition Report

Page 1

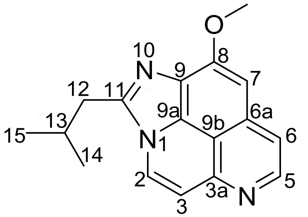

5

Tolerance = 3.0 PPM / DBE: min = -1.5, max = 50.0
Selected filters: None

Monoisotopic Mass, Even Electron Ions
5 formula(e) evaluated with 1 results within limits (up to 50 closest results for each mass)
Elements Used:
C: 5-20 H: 5-20 N: 1-3 O: 1-1

SIPI
M.W=279
Q-Tof micro
YA019
03-Jan-2014,14:41:48
WQ14-004H 23 (0.794) AM (Cen,4, 80.00, Ar,5000.0,268.14,0.70); Sm (Mn, 2x3.00); Cm (23:38)
TOF MS ES+
3.93e4

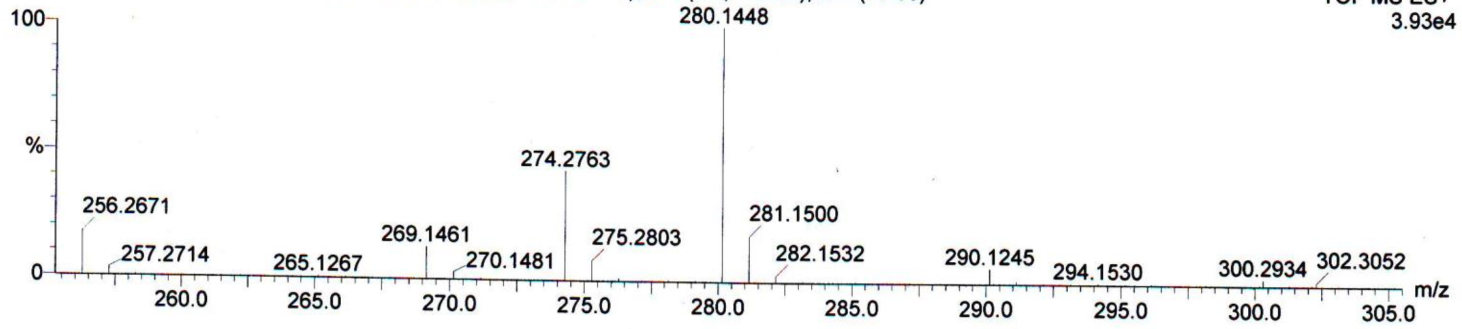

Minimum:	85.00				-1.5		
Maximum:	100.00		5.0	3.0	50.0		
Mass	RA	Calc. Mass	mDa	PPM	DBE	i-FIT	Formula
280.1448	100.00	280.1450	-0.2	-0.7	10.5	64.9	C17 H18 N3 O

Figure S50. UV spectrum of compound **5**.

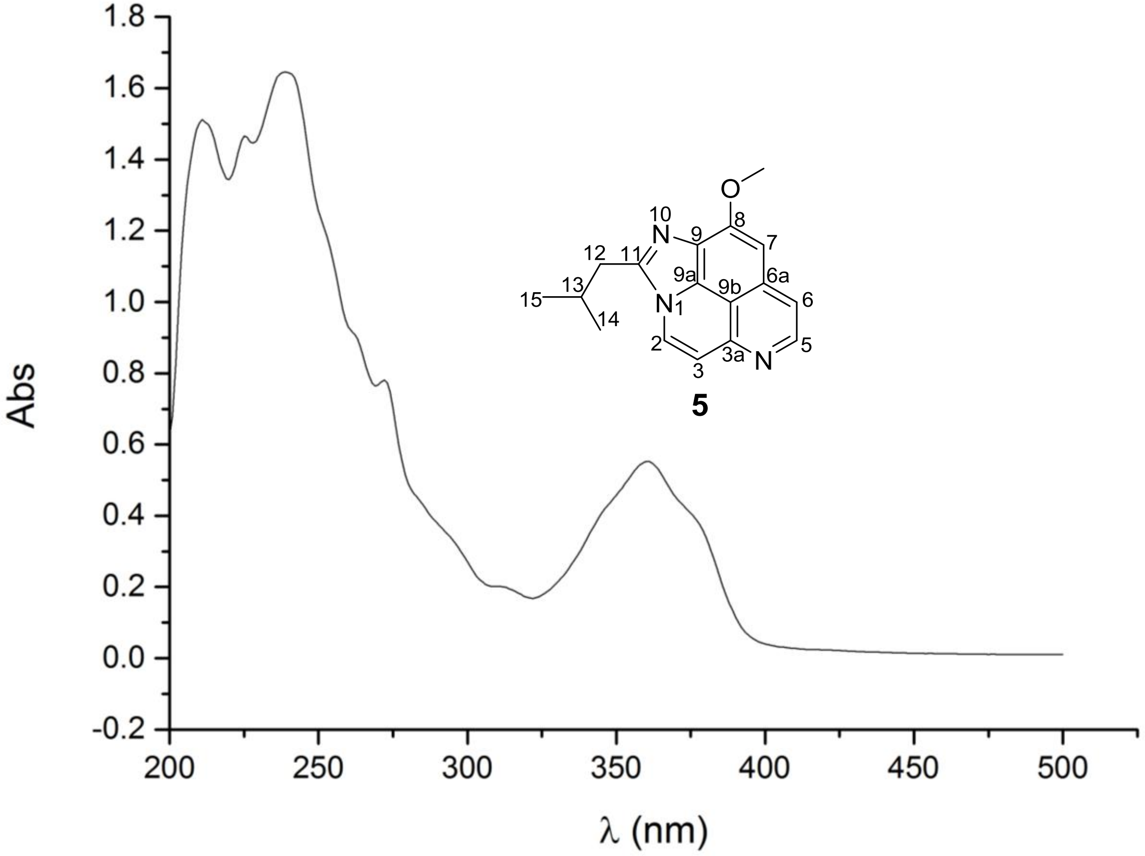

Figure S51. ^{1}H NMR spectrum of compound **6** in $CDCl_3$.

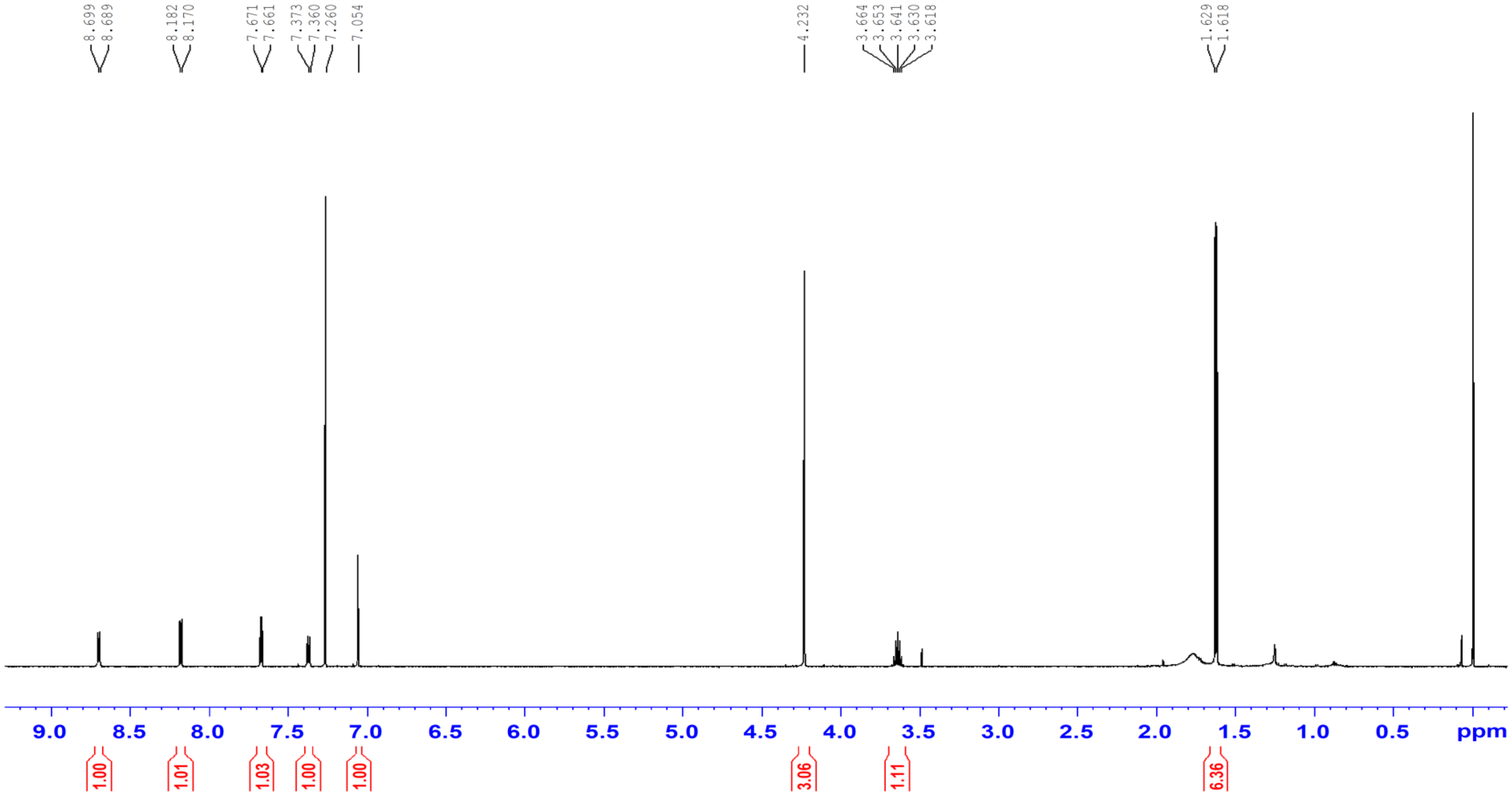

Figure S52. ^{13}C NMR spectrum of compound **6** in $CDCl_3$.

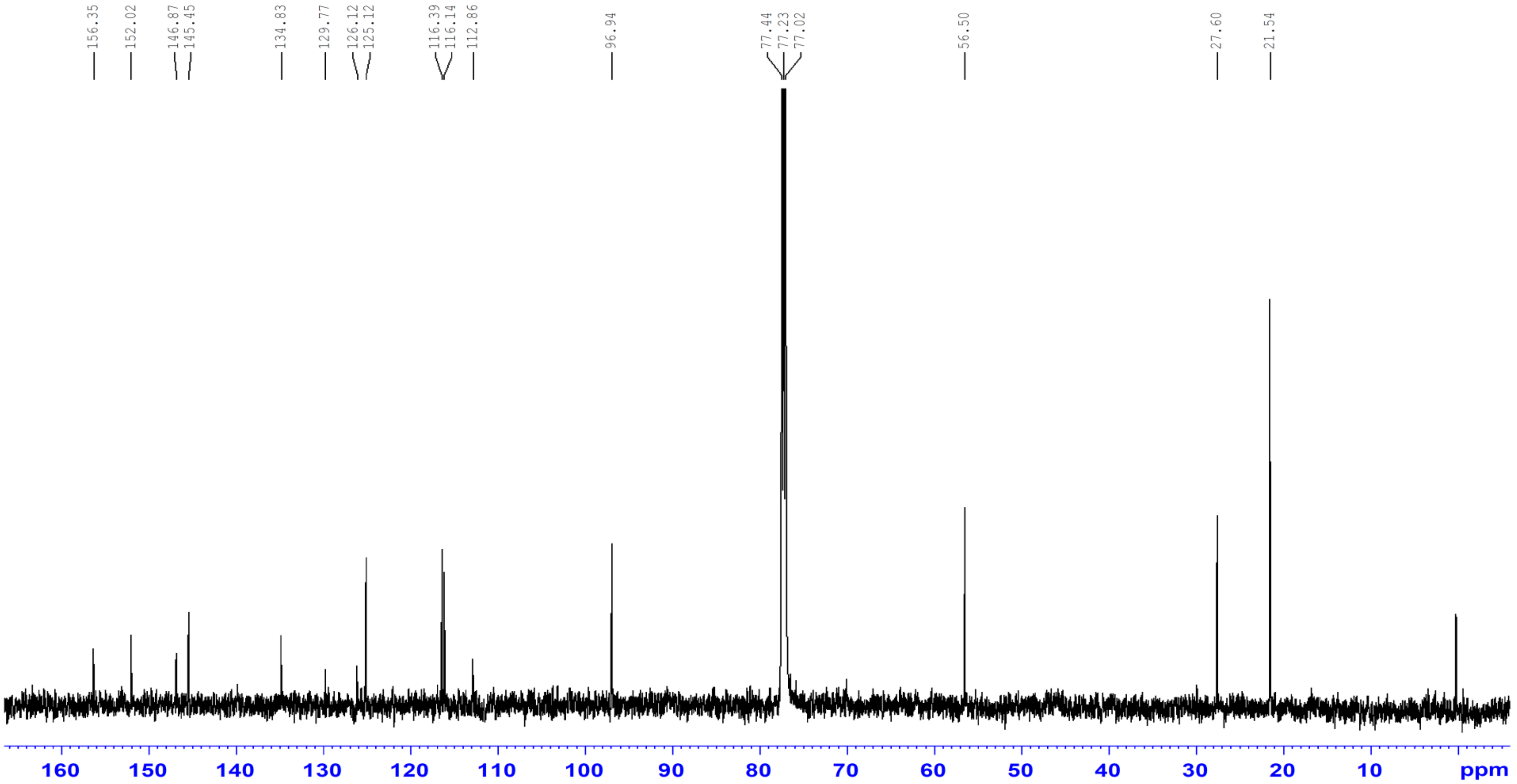

Figure S53. ^{1}H NMR spectrum of compound **7** in $CDCl_3$.

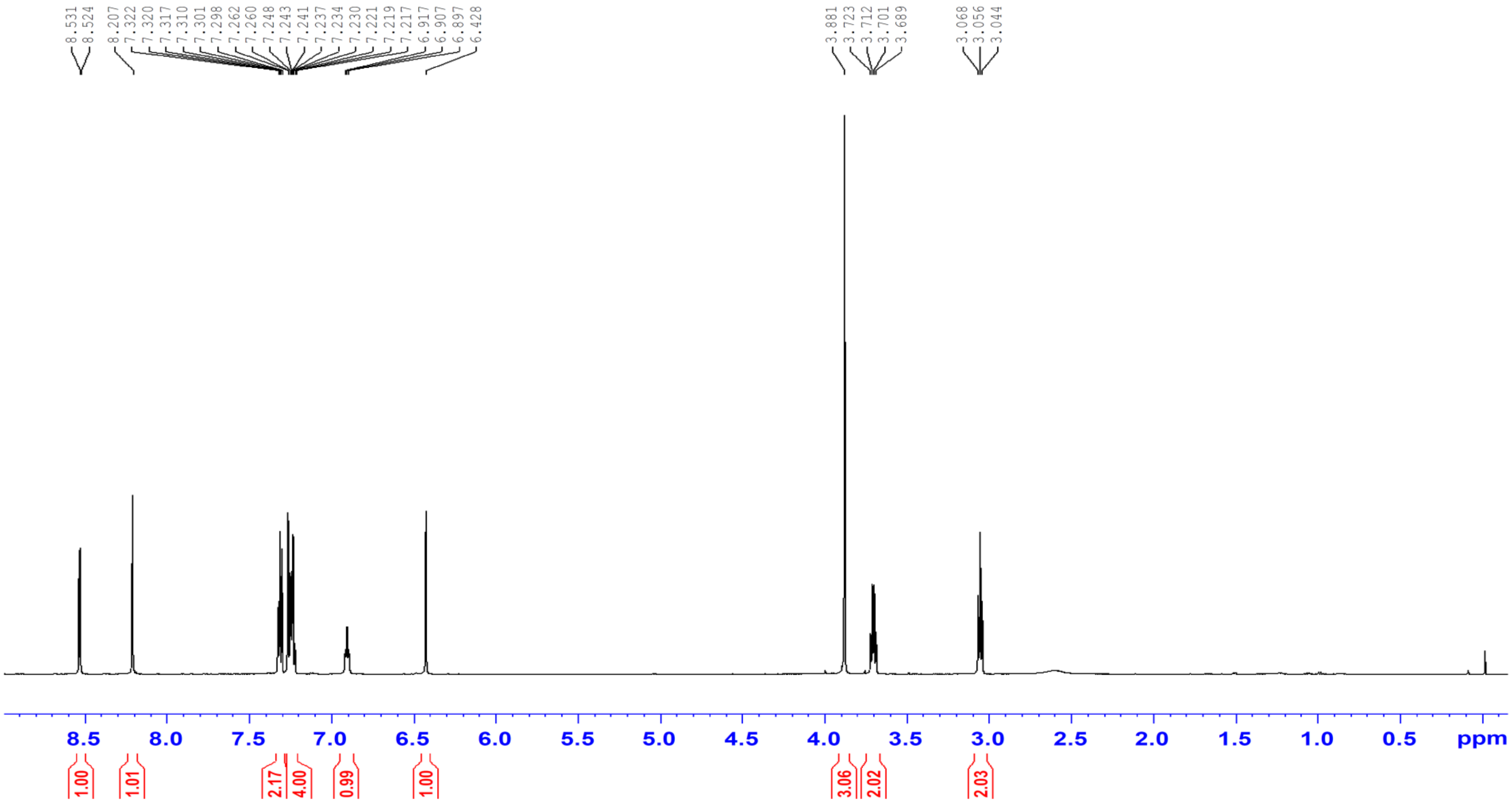

Figure S54. ^{13}C NMR spectrum of compound **7** in $CDCl_3$.

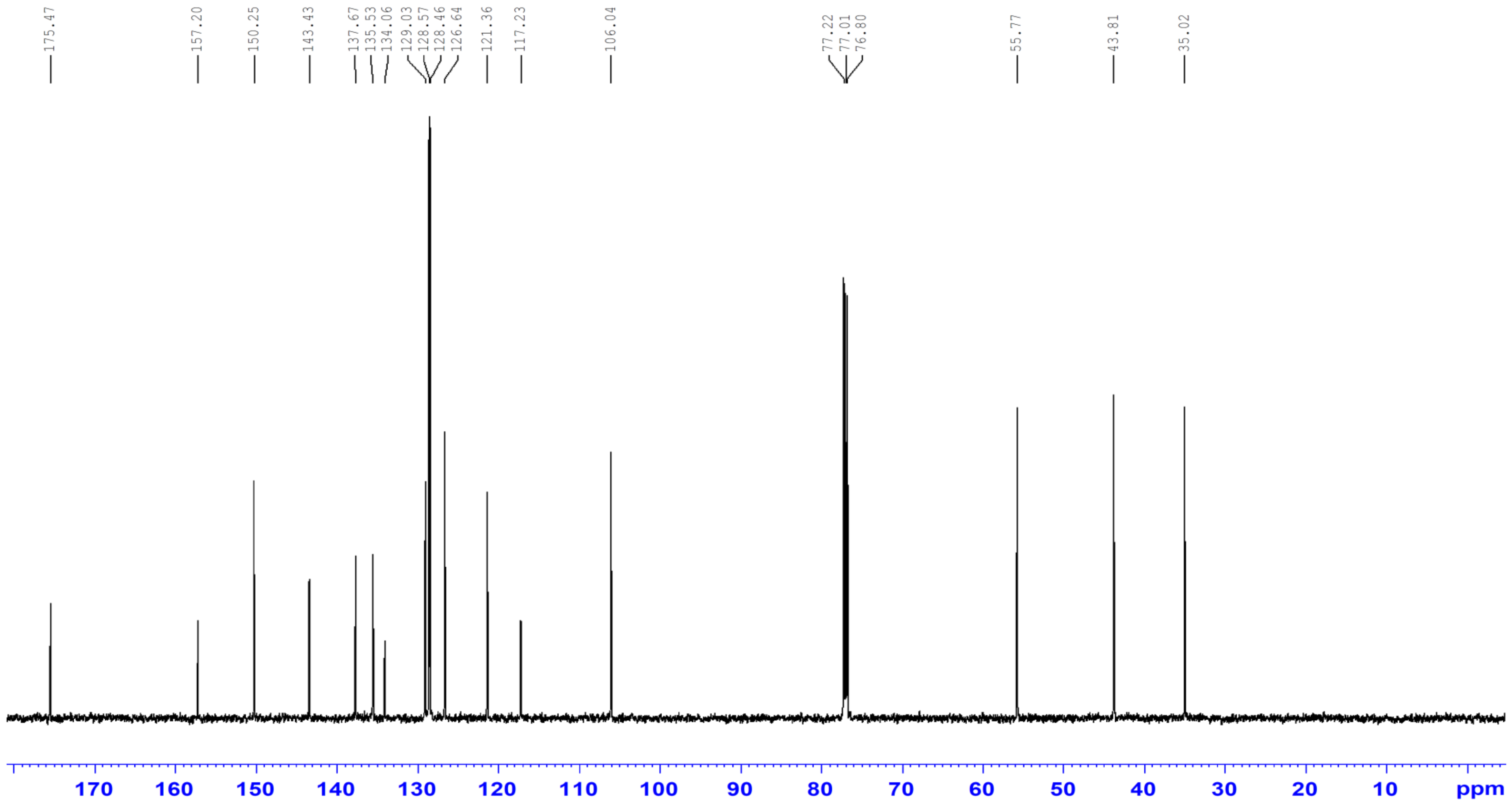

Figure S55. ^{1}H NMR spectrum of compound **8** in $CDCl_3$.

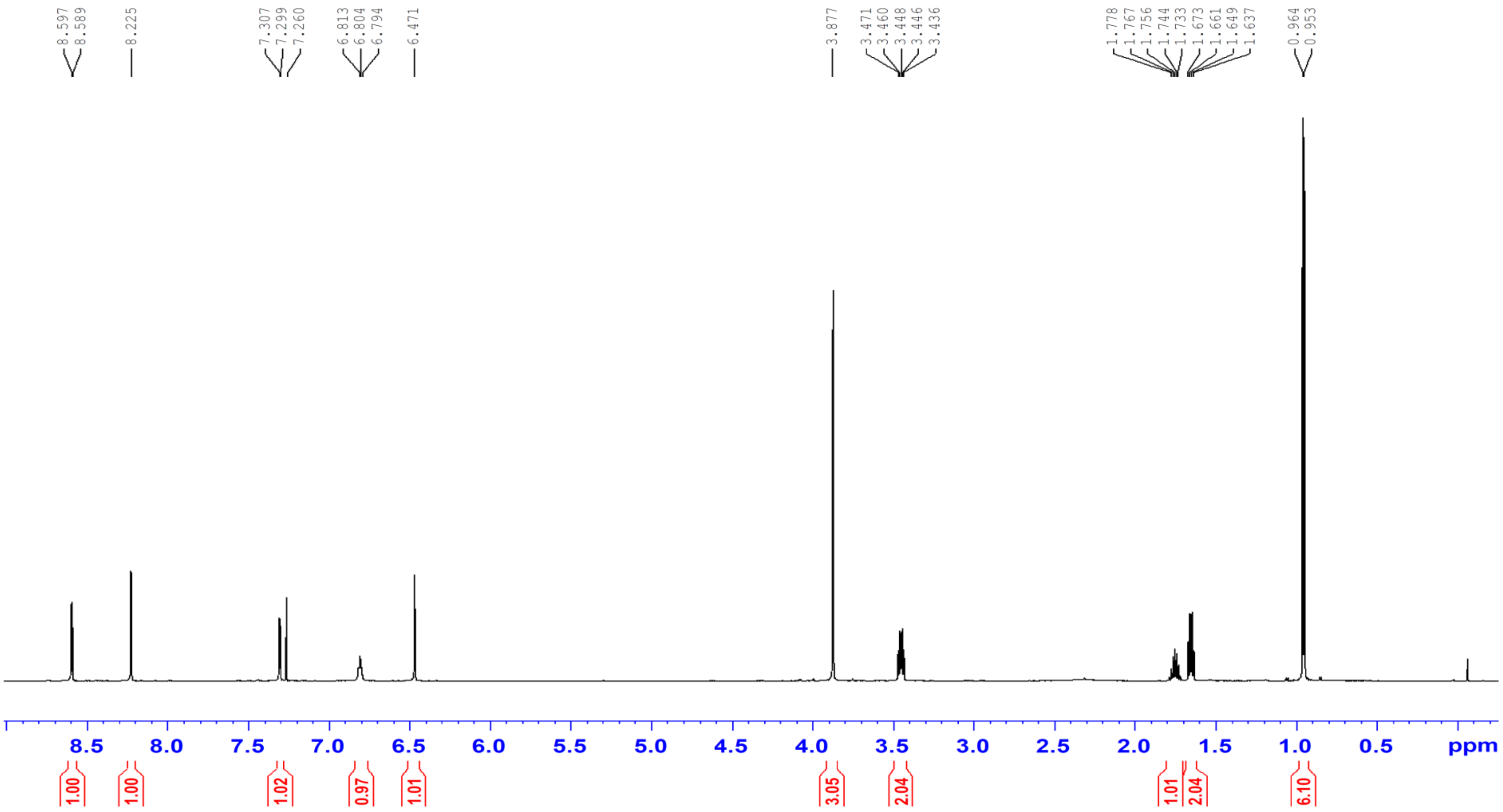

Figure S56. ^{13}C NMR spectrum of compound **8** in $CDCl_3$.

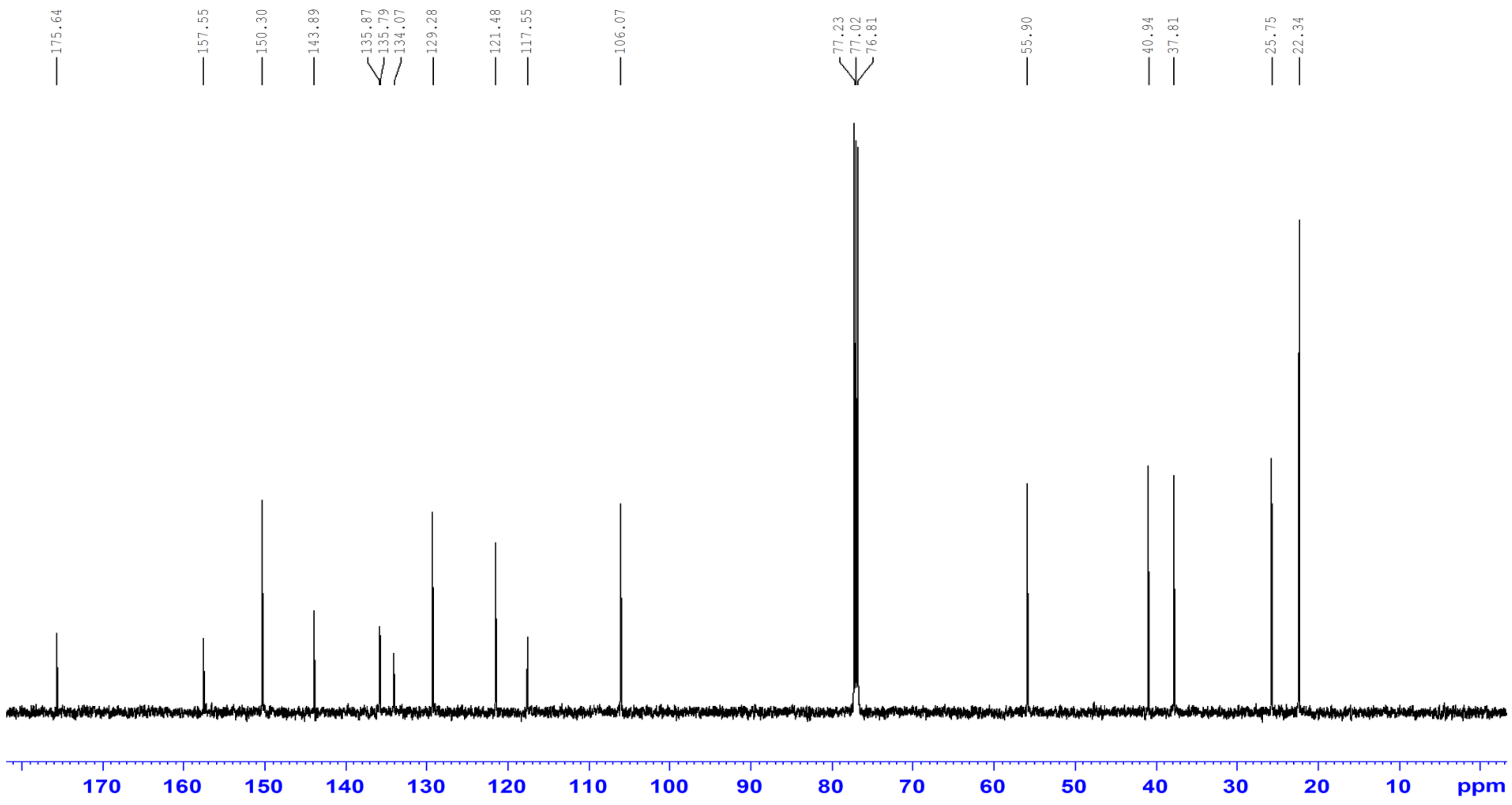

Table S1. NMR Spectroscopic Data for Compounds **6**–**8** in $CDCl_3$.

Position	**6** [a]		**7** [a]		**8** [a]	
	δ_C	δ_H, mult. (J in Hz)	δ_C	δ_H, mult. (J in Hz)	δ_C	δ_H, mult. (J in Hz)
2	125.1, CH	8.69, d, (6.0)	129.0, CH	8.21, s	129.3, CH	8.22, s
3	116.1, CH	7.37, d, (7.2)	143.4, qC		143.9, qC	
3a	146.9, qC		135.5, qC		135.8, qC	
5	145.4, CH	8.17, d, (7.2)	150.3, CH	8.53, d, (4.2)	150.3, CH	8.59, d, (4.2)
6	116.4, CH	7.67, d, (6.0)	121.4, CH	7.32, d, (4.2)	121.5, CH	7.30, d, (4.2)
6a	134.8, qC		135.5, qC		135.8, qC	
7	96.9, CH	7.05, s	106.1, CH	6.43, s	106.1, CH	6.47, s
8	156.4, qC		157.2, qC		157.6, qC	
9	126.1, qC		175.5, qC		175.6, qC	
9a	129.8, qC		134.1, qC		134.1, qC	
9b	112.9, qC		117.2, qC		117.6, qC	
8-OCH_3	56.5, CH_3	4.23, s	55.8, CH_3	3.88, s	55.9, CH_3	3.88, s
11	152.0, qC					
12	27.6, CH	3.64, m				
13	21.5, CH_3	1.62, d, (7.2)				
14	21.5, CH_3	1.62, d, (7.2)				
1'				6.91, t, (6.0)		6.80, t, (6.0)
2'			43.8, CH_2	3.70, q, (13.8, 7.2)	40.9, CH_2	3.45, m
3'			35.0, CH_2	3.06, t, (7.2)	37.8, CH_2	1.65, q, (13.0, 7.2)
4'			137.7, qC		25.8, CH	1.75, m
5'			128.5, CH	7.23, m	22.3, CH_3	0.96, d, (6.6)
6'			128.6, CH	7.30, m	22.3, CH_3	0.96, d, (6.6)
7'			126.6, CH	7.23, m		
8'			128.6, CH	7.30, m		
9'			128.5, CH	7.23, m		

[a] Measured at 600 MHz (^{1}H) and 150 MHz (^{13}C).
